# En Route to Furan-Fused Naphthopyrones: Formal Synthesis of the (+)-Lasionectrin and Its C12-Epimer

**DOI:** 10.1021/acs.joc.3c02231

**Published:** 2023-12-04

**Authors:** Pedro López-Mendoza, Luis F. Porras-Santos, José Alvano Pérez-Bautista, Leticia Quintero, Jocelyn Bautista-Nava, David F. León-Rayo, Alejandro Cordero-Vargas, Fernando Sartillo-Piscil

**Affiliations:** † Centro de Investigación de la Facultad de Ciencias Químicas, Benemérita Universidad Autónoma de Puebla (BUAP), 14 Sur Esq. San Claudio, Col. San Manuel, 72570 Puebla, México; ‡ Instituto de Química, Universidad Nacional Autónoma de México, Circuito Exterior Ciudad Universitaria, 04510 CDMX, México

## Abstract

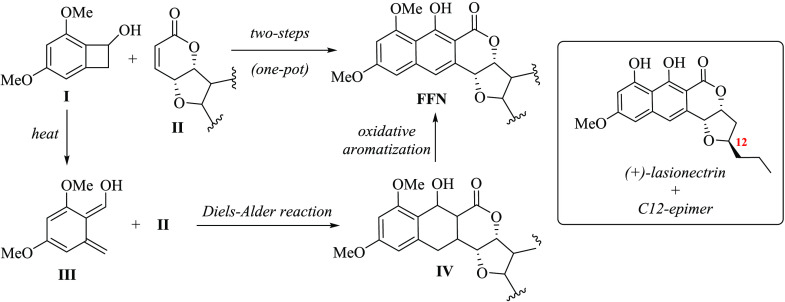

Despite the vast presence of the furan-fused naphthopyrone (FFN) skeleton in many bioactive natural products, such as lasionectrin, at present, a general approach to FFNs has not been developed yet. For that reason, a simple and straightforward synthetic approach consisting of a sequential procedure of a Diels–Alder reaction between 1,3-dimethoxy-benzocyclobutenol **I** and furan-fused-α,β-unsaturated-δ-lactones **II** (via an ο-quinodimethane intermediate **III**) followed by an oxidative aromatization of the corresponding Diels–Alder adduct **IV** is reported. Subsequently, the formal synthesis of the (+)-lasionectrin and its C12-epimer was achieved, the latter in only six steps.

## Introduction

**Figure 1:**
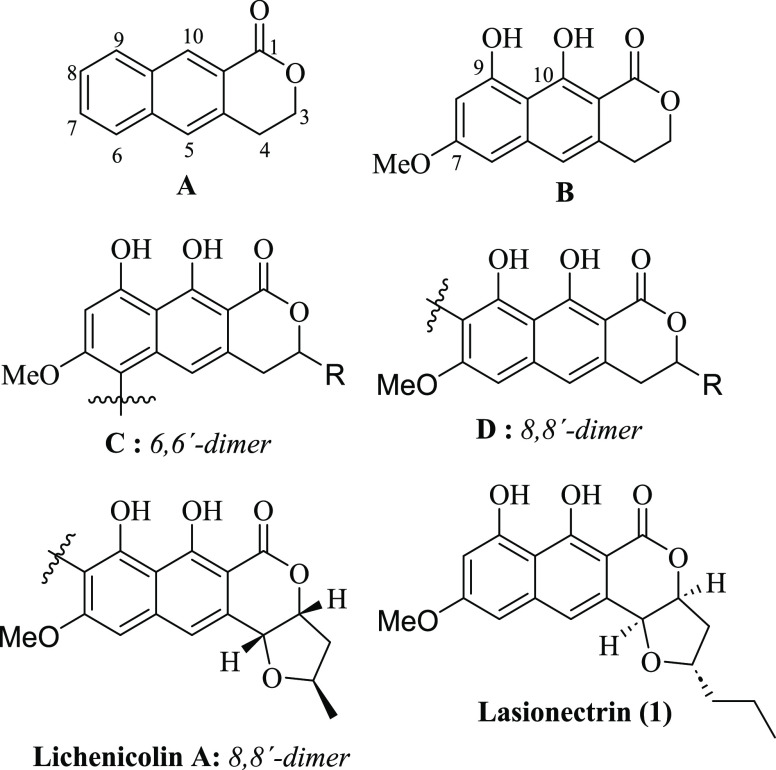
Mono- or dimeric naphthopyrone skeletons in bioactive natural products.

In recent years, the isolation of natural products with a 1*H*-naphtho-[2,3-*c*]pyran-1-one structural skeleton (**A**; also known as naphthopyrone), which is thought to be directly responsible for the high bioactivity against numerous diseases, has increased.^1^ This attractive motif is commonly decorated with hydroxyl groups at C9 and C10 positions and a methoxy group at C7 position (**B**), and it exists in either monomeric or dimeric forms like asymmetric bis-naphthopyrones **C** and **D**.^1b^ Moreover, an emergent naphthopyrone subclass with high bioactivity against infectious microorganisms^2^ includes those structures that contain an additional fused tetrahydrofuran ring, e.g., lasionectrin **1**(2a) or lichenicolin A^2b^ (Figure 1).

**Scheme 1:**
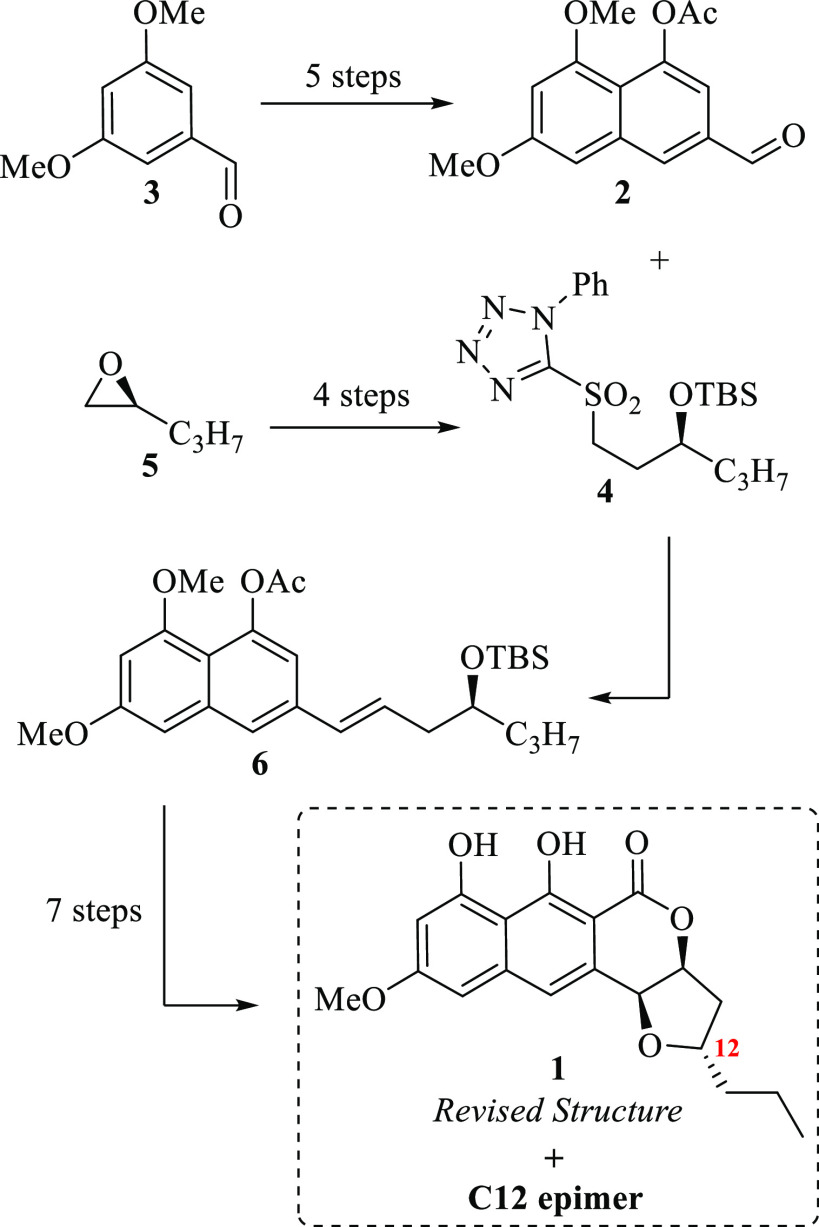
Total Synthesis of Lasionectrin (**1**) and Its C12-Epimer by Brimble and Furkert

Despite the relevance of furan-fused naphthopyrones (FFNs), only lasionectrin **1** has been synthesized^3^ while lichenicolin A remains elusive. Perhaps the main reason for this scarcity is the lack of general synthetic approaches to construct the naphthopyrone skeleton,^4^ especially for FFNs. In this context, Brimble and Furkert achieved the first total synthesis of the antimalarial natural product lasionectrin **1** and revised its absolute configuration. They used Julia-Kocienski olefination as a key step to join naphthaldehyde **2** and enantioenriched sulfone **4**. Additionally, seven steps were required to construct the FFN system from alkene **6** to complete the synthesis of lasionectrin **1** and its C12-epimer in 17 steps from known benzaldehyde **3** and epoxide **5** (Scheme 1).

## Results and Discussion

**Scheme 2:**
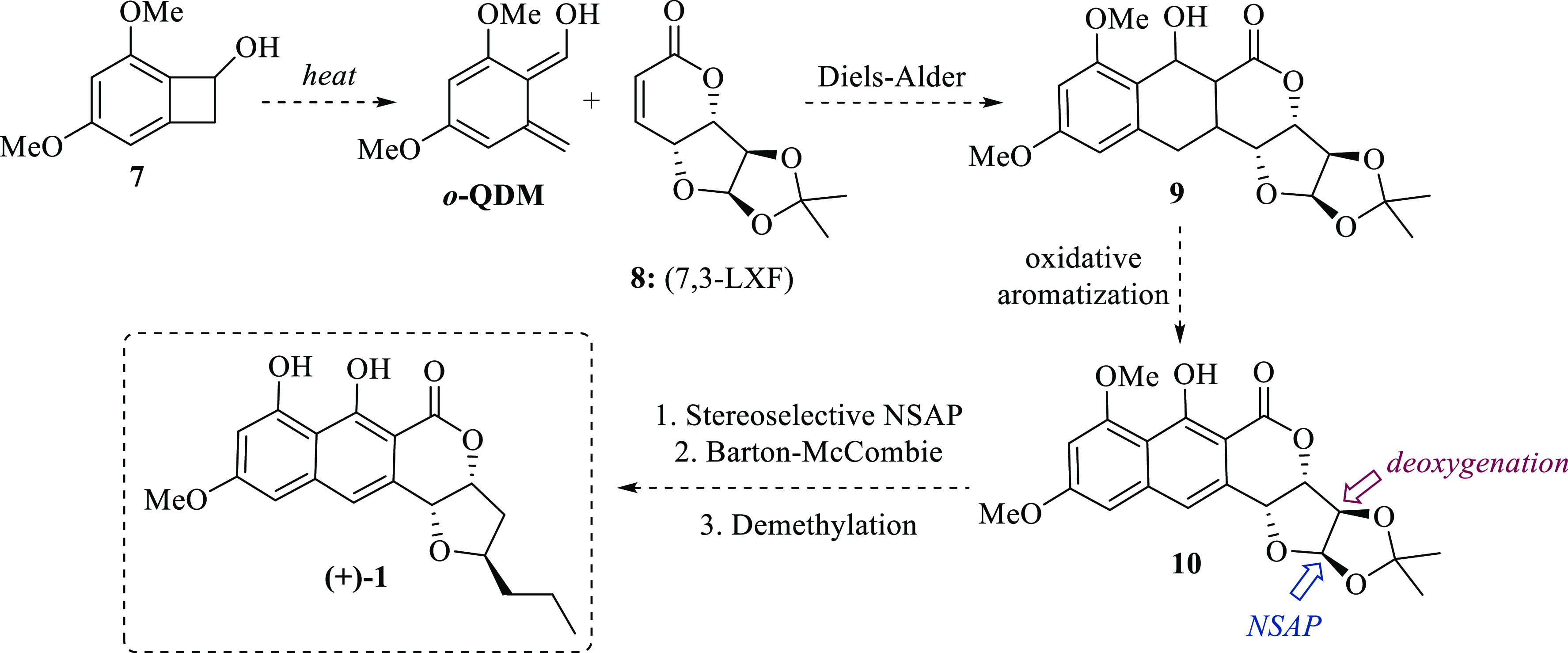
Approach to FFN **10** and Total Synthesis Plan of Lasionectrin (+)-**1**

Motivated by their biological relevance and structural complexity, we launched a research project aimed to develop a concise and modular approach to FFNs in our laboratory. Consequently, it was envisioned the construction of the naphthopyrone core through a [4 + 2] cycloaddition^5^ between an ο-quinodimethane intermediate **(o-QDM)** (generated by heating 1,3-dimethoxy-benzocyclobutenol **7**) and the unsaturated 7,3-δ-lactone-xylofuranose **8** (7,3-LXF),^6^ followed by an oxidative aromatization process of the respective Diels–Alder adduct **9**. On one side, the 1,3-dimethoxy-benzocyclobutenol **7** would provide the required oxygenated functionality within the aromatic system; on the other side, the chiral furan *cis*-fusion would be provided by the lactone **8**. Additionally, it was planned that a stereoselective nucleophilic substitution at the anomeric position (NSAP)^7^ on the furanose ring of **10** followed by a deoxygenation reaction at C11 could enable us to tackle the proposed structure of the (+)-lasionectrin **1**: an antimalarial natural product isolated by Reyes and co-workers^2a^ from the fermentation of the fungus *Lasionectria* (F-176,994) (Scheme 2).

**Scheme 3:**
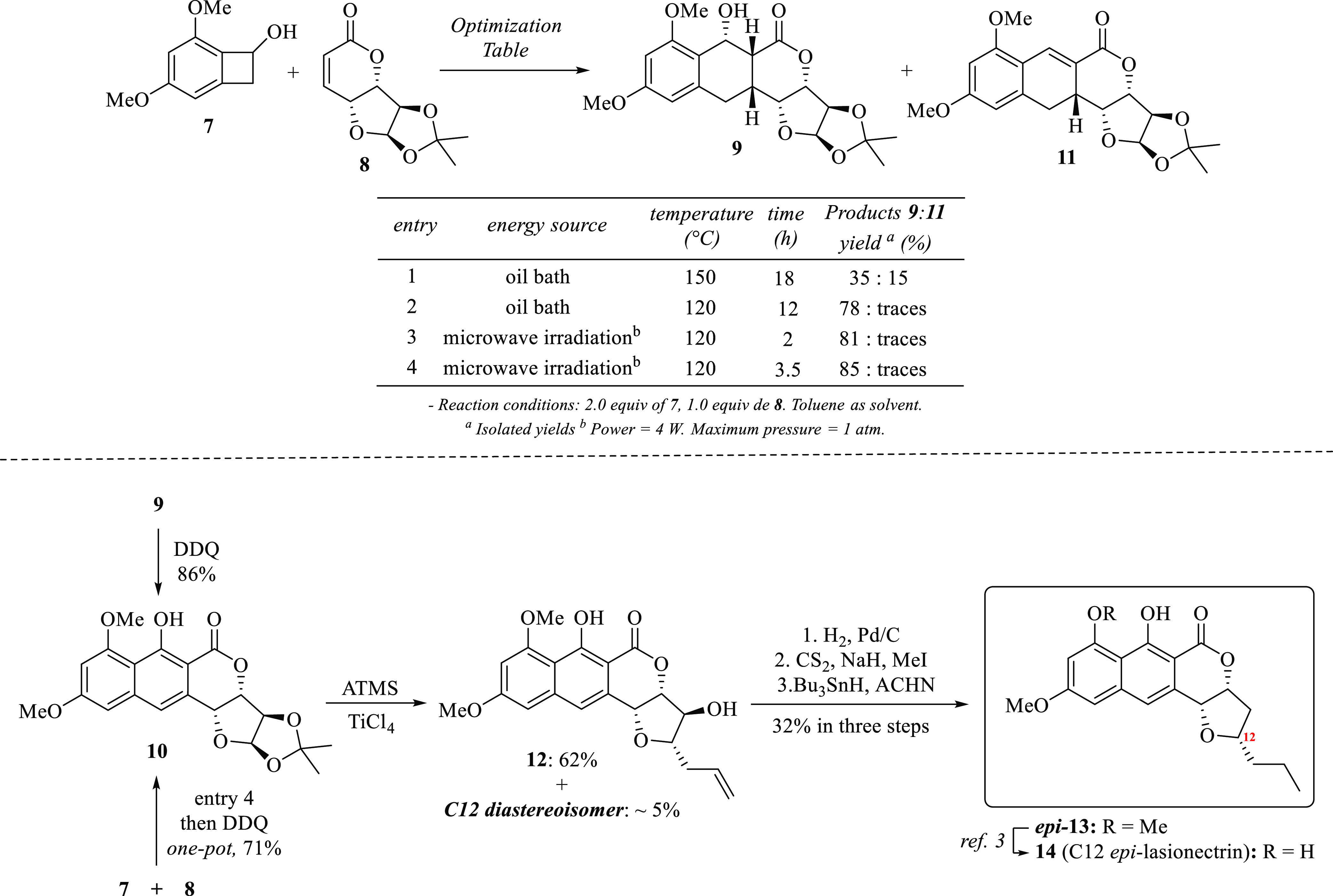
Execution of the Synthesis Plan for Lasionectrin (+)-**1**

The straightforward preparation of benzocyclobutenol **7**(8) and chiral furan-fused α,β-unsaturated δ-lactone **8**(6) from bromo 3,5-dimethoxybenzene and diacetone-d-glucose (DAG), respectively, permitted the rapid execution of the synthesis plan in only six steps (Scheme 3). By refluxing a mixture of two equivalents of **7** with one equivalent of **8** in dry toluene at 150 °C for 18 h in a sealed tube, the Diels–Alder adduct **9** was obtained in 35% yield, along with a dehydrated product **11** in 15% (Scheme 3, entry 1). When the reaction temperature was lowered to 120 °C for 12 h, the chemical yield of **9** was increased to 78% and only traces of **11** were observed (entry 2). Interestingly, when conventional heating was switched to microwave irradiation, the adduct **9** was obtained in 81% yield in only 2 h at 120 °C (entry 3) and 85% yield in 3.5 h (entry 4). Hence, with the optimal Diels–Alder reaction conditions in hand (entry 4), the FFN **10** was obtained in 86% yield after treating adduct **9** with 2,3-dichloro-5,6-dicyano-1,4-benzoquinone (DDQ) in toluene for 8 h at room temperature. Further, when the entire chemical process was carried out as a one-step protocol (**7** + **8** → **9** + DDQ → **10**), the chemical yield was almost the same. Accordingly, a novel synthetic strategy to chiral furan-fused naphthopyrones (e.g., **10**) in only two steps with a 71% overall chemical yield is established (Scheme 3).

**Scheme 4:**
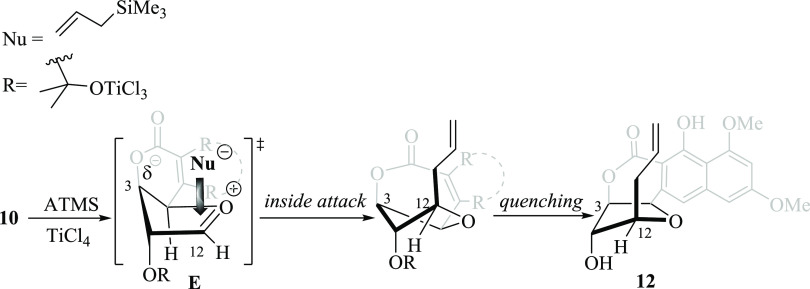
Woerpel’s Model for the Stereoselective NSAP of **10**

Following the synthesis plan, the NSAP reaction on **10** with allyltrimethylsilane (ATMS) and TiCl_4_, as a Lewis acid, was executed. While we expected that the steric factors could direct the nucleophilic attack *anti* to the naphthopyrone moiety, thus placing the allyl substituent at the C12 position with the correct absolute configuration, the major product was the undesired *C*-allylated product **12**. Unfortunately, the expected *C*-glycoside was obtained in an approximately 5% yield. Apparently, the electrostatic interactions imposed by the substituent at C3 position (with respect to oxocarbenium positive ion center of **E** in Scheme 4) mediated the stereoselective substitution *cis* to the naphthopyrone, as the Woerpel’s “inside attack” model states for the NSAP of furanose carbohydrate derivatives.^7,9^ In this regard, the preferred approach of ATMS to the more stable conformation of the oxocarbenium ion intermediate **E**, occurred *syn* to the OR group placed at the C3 position (Scheme 4). Although we made further efforts to revert the stereochemical outcome by changing temperature, solvent, and Lewis acid (BF_3_·OEt_2_ and TMSOTf), none of them were successful. Clearly, the steric hindrance is much less important than the electrostatic and stereoelectronic factors imposed by oxocarbenium ion intermediate **E**. Thereafter, double-bond reduction of **12** with H_2_ and Pd/C followed by a Barton-McCombie deoxygenation reaction gave FFN ***epi***-**13**, the precursor of C12 *epi*-lasionectrin **14**.

**Scheme 5:**
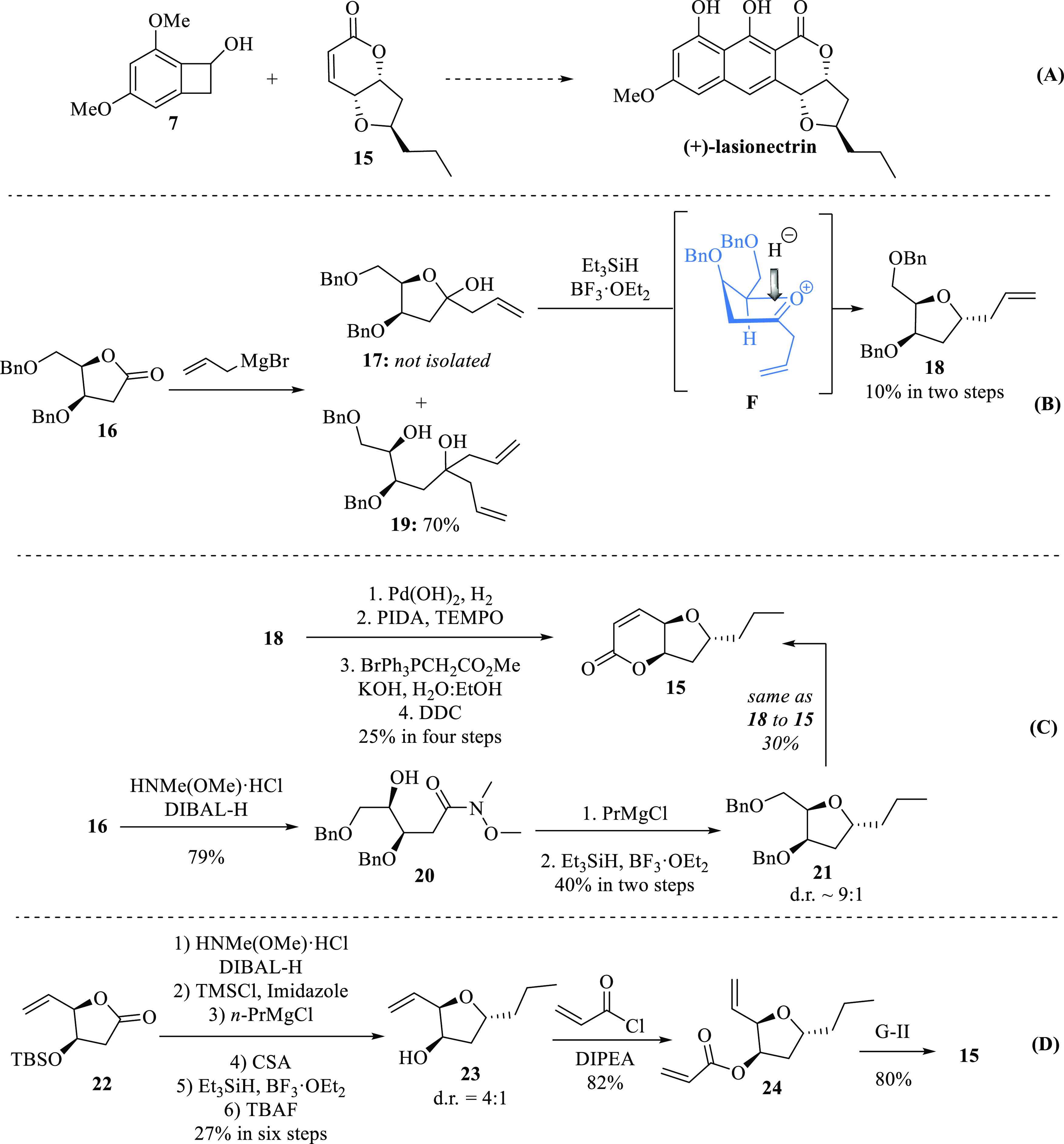
Convergent Approach to (+)-Lasionectrin (A). Synthesis of Lactone Fused Furan **15** (B–D)

At this point, we realized that chiron **8** was suitable for accessing to *epi*-lasionectrin **14** from known lactone **8**, but not to (+)-lasionectrin **1**. We understood that the only way to access (+)-**1** would be through a convergent approach: the application of the Diels–Alder/aromatization protocol to furan-fused α,β-unsaturated δ-lactone **15** (Scheme 5A). Hence, the stereoselective introduction of the propyl substituent at the C12 position of the tetrahydrofuran ring was envisioned by applying a two-step Kishi *C*-glycosylation protocol^10^ over the known γ-lactone **16**,^11^ which involves the addition of an organometallic reagent to generate the lactol intermediate **17**, followed by a stereoselective reduction to obtain **18**, which eventually would be transformed into **15**. Thus, when lactone **16** reacted with allylMgBr at a low temperature, a mixture of lactol **17** and tertiary alcohol **19** was obtained. Even though lactol **17** was the minor product, the reaction mixture was subjected to stereoselective reduction with triethylsilane and BF_3_·OEt_2_, generating 1,3-*anti C*-glycoside **18** in only 10% yield, via the “inside attack” of hydride over oxocarbenium ion **F** (Scheme 5B). Further attempts to improve this low chemical yield with other organometallic reagents such as allylMgCl and allyllithium were useless.

Then, the required lactone **15** was obtained from **18** in a sequential four-step one-column purification procedure with a moderated 25% overall yield. First, debenzylation plus double-bond reduction with Pd(OH)_2_ and H_2_; then, selective oxidation of the primary hydroxyl group with phenyliodine(III)diacetate (PIDA) and catalytic amounts of 2,2,6,6-tetramethylpiperidinyloxy (TEMPO) in dichloromethane (DCM) to the transient aldehyde (not shown),^12^ which was subjected to selective Wittig olefination with BrPh_3_PCH_2_CO_2_Me in aqueous KOH to the respective δ-hydroxy carboxylic acid (not shown),^13^ and final lactonization with dicyclohexylcarbodiimide (DCC) in DCM gave **15** (Scheme 5C). Since the *C*-glycosylation step was problematic due to the double addition of Grignard reagent onto carbonyl of **16** to produce tertiary alcohol **19**, it was then explored a Weinreb amide approach.^14^ Accordingly, the amide **20** was synthesized by treating lactone **16** with an organoaluminum intermediate generated from DIBAL–H and the hydrochloride of *N*-methoxy-*N*-methyl amine (Weinreb amine).^15^ Then, by adding PrMgCl to **20** followed by stereoselective reduction with triethylsilane and BF_3_·OEt_2_, tetrahydrofuran **21** was obtained with moderate yield (44%) and high stereoselectivity (dr ∼9:1). Finally, **15** was obtained in 30% yield by following the same steps as for **18** → **15** (Scheme 5C). Since the sequential protocol for the construction of the ring in **15** was inefficient, a ring-closing metathesis approach was envisioned (Scheme 5D). In consequence, the known TBS-protected γ-vinyl lactone **22**(16) was transformed into tetrahydrofuran **23** in sequential six steps with only one-column purification. First, the application of a modified Weinreb amide protocol, which includes the temporary protection of the hydroxyl group with trimethylsilyl chloride (TMSCl) to prevent conflicts with the Grignard reagent.^17^ Then, selective deprotection of the TMS group with camphor sulfonic acid (CSA) followed by the stereoselective reduction with triethylsilane and BF_3_·OEt_2_ and removal of the TBS group with tetrabutylammonium fluoride (TBAF) afforded the required tetrahydrofuran **23** in 27% overall yield with a diastereomeric ratio ∼4:1 (Scheme 5D). Finally, acylation of **23** to **24** with acryloyl chloride under basic conditions followed by ring-closing metathesis with second-generation Grubbs catalyst^18^ afforded **15** in high chemical yield. Certainly, this is the most efficient route to obtain required dienophile **15** (18% overall yield from **22**).

**Scheme 6:**
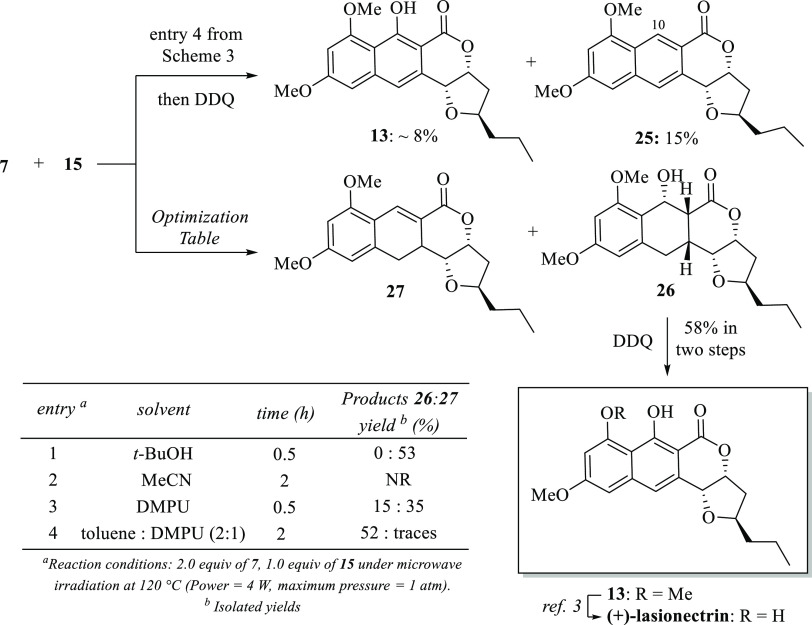
Completion of the Synthesis of (+)-Lasionectrin

Having in hands lactone **15**, the final stage of the synthesis of the lasionectrin (+)-**1** was accomplished (albeit not as easy as we expected). Under the optimized reaction conditions displayed in Scheme 3, most of the lactone **15** remained unchanged, and the desired lasionectrin precursor **13** was obtained in an unexpectedly low chemical yield (∼8%) along with a 10-deoxygenated naphthopyrone **25** as the major product (Scheme 6). We were intrigued by this dramatic difference in the reactivity between unsaturated lactone **8** and **15**. By analyzing both molecular structures, we realized that the high reactivity of lactone **8** might be explained in terms of a dienophile strain caused by the *O*-isopropylidene moiety fused to furan ring resulting in a highly productive Diels–Alder cycloaddition.^19^ Thus, to overcome the lack of reactivity of lactone **15**, the influence of the solvent was explored. Based on reports in which protic polar solvents accelerate the Diels–Alder reactions,^20^ the cycloaddition reaction was carried out with *t*-BuOH. Although the reaction rate was significantly accelerated, the major product was the dehydrated product **27** instead of the expected Diels–Alder adduct **26** (entry 1). Looking for an aprotic polar solvent, the cycloaddition reaction was performed with acetonitrile (MeCN), but the starting material remained unchanged (entry 2). Satisfactorily, we obtained the expected adduct **26** in 15% yield along with **27** in 35% yield when *N*,*N*′-dimethylpropyleneurea (DMPU) was used as solvent (entry 3). The latter experiment encouraged us to use a mixture of solvents. Accordingly, a 2:1 mixture of toluene/DMPU was adequate to improve the chemical yield of the adduct **26** up to 52% yield, and only traces of the dehydrated product **27** were observed (entry 4). Different mixtures of these solvents resulted in a decreased yield. Finally, aromatization of adduct **26** with DDQ afforded (+)-lasionectrin precursor **13** in good yield. NMR data and optical rotation **[α]**_***D***_^**28**^ + 64.0 (c 0.100, CHCl_3_) are consistent with data reported by Brimble **[α]**_***D***_^**28**^ + 120.0 (c 0.50, CHCl_3_), whereupon confirming the Brimble’s structural revision.

## Conclusions

In summary, we have developed a concise synthesis to (+)-lasionectrin and its C12-epimer by featuring a two-step protocol that involves a Diels–Alder reaction and an oxidative aromatization of the respective adduct with DDQ. The current syntheses represent a novel approach that not only enables a new access to the enantiomer of the title natural product and its C12-epimer in a more concise way than the one reported but also might offer an efficient synthetic technology for accessing a larger number of either natural occurring or synthetic furan-fused naphthopyrones. Additionally, with the use of the Weinreb amide approach to the *C*-glycosylation of γ-lactones **16** and **22**, we stand in the position to attach almost any alkyl or aryl group at C12 position to thus establish a general stereoselective strategy for the construction of novel furan-fused naphthopyrones with potential use in medicinal chemistry. Accordingly, we undertake this challenge, and the progress in this project will be disclosed soon.

## Experimental Part

### General Information

All reactions were carried out under an inert nitrogen atmosphere with dry solvents under anhydrous conditions unless otherwise noted. Commercially available reagents were purchased from Sigma-Aldrich and used without further purification unless otherwise noted. *tert*-Butanol, acetonitrille, tetrahydrofuran (THF), dichloromethane (DCM), diethyl ether, toluene, and methanol were used as a reactive grade, dried under standard techniques, and freshly distilled prior to use. Column chromatography (CC) was performed using silica gel 230–400 mesh as stationary phase and a mixture of solvents as mobile phase, as indicated. Reactions were monitored by thin-layer chromatography on 0.25 mm Merk silica gel 60-F254 plates using ultraviolet (UV) light or anisaldehyde or ammonium molybdate stain as visualizing agents. Melting points were measured on a Fisher Scientific 12–144 melting point apparatus and are not corrected. Microwave experiments were carried out in a microwave reactor CEM Discover System (model 908005) in sealed tubes. Specific rotations were measured with a Rudolph Research Analytical Autopol III automatic polarimeter. High-resolution mass spectrometry was carried out in a JEOL DART AccuTOF JMS-T100CC spectrometer. NMR spectra were recorded on a Bruker-500 (500 MHz) using as reference: TMS (0.0 ppm for ^1^H) and residual solvent peak of CDCl_3_ (δ = 7.26 ppm for ^1^H NMR and δ = 77.16 ppm for ^13^C); chemical shifts (δ) are stated in parts per million (ppm) and hertz (Hz) for the coupling constants (*J*). The following abbreviations (or combinations thereof) were used to explain the multiplicities: s = singlet, d = doublet, t = triplet, q = quartet, m = multiplet, and br = broadened.

#### 3,5-Dimethoxybicyclo[4.2.0]octa-1,3,5-trien-7-ol (**7**)^8^

To a flame-dried flask was added anhydrous THF under a nitrogen atmosphere. The solvent was cooled at 0 °C, and *n*-BuLi (2.5 M in hexanes, 8.0 mL, 37.5 mmol) was added dropwise, then warmed to room temperature, and stirred for 16 h to generate the acetaldehyde lithium enolate (CH_2_=CH–OLi). Afterward, the reaction was cooled to −78 °C and a solution of 1-bromo-3,5-dimethoxybenzene (3.2 g, 14.7 mmol) in THF (3.2 mL) was added. Subsequently, freshly prepared LiTMP (1.1 M in THF, 17.7 mmol) was added dropwise, and the reaction was stirred at −78 °C for 16 h. Then, the flask was removed from the cold bath and immediately quenched with a saturated aqueous NH_4_Cl solution (30 mL). The mixture was then warmed to rt and extracted with ethyl acetate (3× 20 mL). The combined organic phase was dried with Na_2_SO_4_, filtered, and concentrated under reduced pressure. The crude was purified by flash column chromatography on silica gel (hexane/ethyl acetate, 8:2) to obtain compound **7** (2.1 g, 80% yield) as a white solid. *R*_f_ = 0.3 (silica gel, hexane/ethyl acetate, 8:2). mp 77–78 °C. ^**1**^**H NMR** (300 MHz, CDCl_3_) δ 6.32 (d, *J* = 1.0 Hz, 1H), 6.25 (d, *J* = 0.9 Hz, 1H), 5.26 (s, 1H), 3.94 (s, 3H), 3.75 (s, 3H), 3.51 (dd, *J* = 14.9, 4.1 Hz, 1H), 2.92 (ddd, *J* = 14.5, 2.6, 0.9 Hz, 1H), 2.40 (s, 1H). ^**13**^**C{**^**1**^**H} NMR** (75 MHz, CDCl_3_) δ: 163.1, 155.8, 144.8, 123.3, 102.0, 100.4, 70.2, 57.2, 55.6, 42.3. High-resolution mass spectrometry (**HRMS**) (DART, TOF) calcd. for C_10_H_13_O_3_ [M + H]^+^ 181.0864, found 181.0862.

#### (3aR,3bS,5aR,6R,11aR,11bR,12aR)-6-Hydroxy-7,9-dimethoxy-2,2-dimethyl-3a,3b,5a,6,11,11a,11b,12a-octahydro-5*H*-[1,3]dioxolo[4′,5′:4,5]furo[3,2-*c*]benzo[*g*]isochromen-5-one (**9**)

In a flame-dried microwave tube, lactone **8** (50 mg, 0.236 mmol, 1.0 equiv) and benzocyclobutenol **7** (85 mg, 0.472 mmol, 2.0 equiv) were dissolved in anhydrous toluene (1.5 mL) under a nitrogen atmosphere, and the mixture was degassed by bubbling nitrogen for 10 min. The tube was sealed, and the reaction was heated at 120 °C for 3.5 h under microwave irradiation. Then, the solvent was evaporated under reduced pressure, and the resulting crude was purified by flash column chromatography on silica gel (hexane/ethyl acetate, 7:3) to obtain compound **9** (78.6 mg, 85% yield) as a white solid. mp 96–98 °C. *R*_f_ = 0.15 (silica gel, hexane/ethyl acetate, 7:3). **[α]**_***D***_^**25**^ –0.13 (c 0.973, CHCl_3_). ^**1**^**H NMR** (500 MHz, CDCl_3_) δ 6.35 (s, 1H), 6.32 (s, 1H), 5.93 (apparent d, *J* = 3.9 Hz, 1H), 5.38 (dd, *J* = 6.1, 2.8 Hz, 2H), 5.14 (apparent d, *J* = 4.7 Hz, 1H), 4.79 (apparent d, *J* = 3.9 Hz, 2H), 4.47 (apparent t, *J* = 5.6 Hz, 2H), 3.83 (s, 3H), 3.80 (s, 3H), 3.112 (d, *J* = 3.7 Hz, 1H), 3.108–3.064 (m, 1H), 2.97 (apparent t, *J* = 6.4 Hz, 1H), 2.86 (dd, *J* = 16.3, 5.9 Hz, 1H), 2.42 (apparent p, *J* = 6.0 Hz, 1H), 1.44 (s, 3H), 1.33 (s, 3H). ^**13**^**C{**^**1**^**H} NMR** (125 MHz, CDCl_3_) δ: 172.4, 160.5, 158.1, 136.2, 118.0, 112.3, 104.8, 104.7, 96.9, 85.1, 83.1, 77.4, 62.4, 55.5, 55.3, 43.1, 32.6, 31.0, 27.0, 26.5. **LRMS** (DART, TOF): *m*/*z* 378[M + H]^+^ (100).

#### (3aR,3bS,11aR,11bR,12aR)-7,9-Dimethoxy-2,2-dimethyl-3a,3b,11,11a,11b,12a-hexahydro-5H-[1,3]dioxolo[4′,5′:4,5]furo[3,2-*c*]benzo[*g*]isochromen-5-one (**11**)

Compound **11** was purified by flash column chromatography on silica gel (hexane/ethyl acetate, 8:2) to obtain a white solid (13.2 mg, 15% yield, Scheme 3, entry 1). mp 187–188 °C. *R*_f_ = 0.30 (silica gel, hexane/ethyl acetate, 7:3). **[α]**_***D***_^**24**^ –138.5 (c 0.687, CHCl_3_). ^**1**^**H NMR** (500 MHz, CDCl_3_) δ 7.97 (s, 1H), 6.35 (s, 1H), 6.30 (s, 1H), 5.94 (d, *J* = 2.9 Hz, 1H), 4.82 (apparent d, *J* = 3.7 Hz, 1H), 4.65 (apparent s, 1H), 4.24 (apparent s, 1H), 3.83 (s, 3H), 3.82 (s, 3H), 3.06 (dd, *J* = 14.7, 3.1 Hz, 1H), 2.93 (dd, *J* = 15.4, 6.0 Hz, 1H), 2.81–2.74 (m, 1H), 1.54 (s, 3H), 1.35 (s, 3H). ^**13**^**C{**^**1**^**H} NMR** (125 MHz, CDCl_3_) δ: 166.2, 162.8, 158.9, 139.3, 134.9, 118.1, 115.0, 112.4, 105.2, 104.4, 96.6, 83.6, 79.7, 77.0, 55.7, 55.6, 34.9, 32.6, 26.6, 26.2. **HRMS** (DART, TOF) calcd for C_20_H_23_O_7_ [M + H]^+^ 375.1443, found 375.1447.

#### (3aR,3bS,11bR,12aR)-6-Hydroxy-7,9-dimethoxy-2,2-dimethyl-3a,3b,11b,12a-tetrahydro-5H-[1,3]dioxolo[4′,5′:4,5]furo[3,2-*c*]benzo[*g*]isochromen-5-one (**10**)

In a flame-dried microwave tube, lactone **8** (150 mg, 0.708 mmol, 1.0 equiv) and benzocyclobutenol **7** (258.0 mg, 1.41 mmol, 2.0 equiv) were dissolved in anhydrous toluene (4.5 mL) under a nitrogen atmosphere, and the mixture was degassed by bubbling nitrogen for 10 min. The tube was sealed, and the reaction was heated at 120 °C for 3.5 h under microwave irradiation. Afterward, the mixture was allowed to cool to room temperature and additional anhydrous toluene (4.5 mL) was added followed by DDQ. The reaction was stirred at room temperature for 8 h. Afterward, the solids were filtered over Celite and washed with toluene. The solvent was evaporated in a high-vacuum pump, and the crude was redissolved in ethyl acetate (10 mL) and washed with an aqueous saturated NaHCO_3_ solution (5 × 5 mL). The organic phase was dried with Na_2_SO_4_, filtered, and concentrated under reduced pressure. The resulting crude product was purified by flash column chromatography on silica gel (hexane/ethyl acetate, 8:2) to obtain compound **10** (194.9 mg, 71% yield) as a white solid. mp 114–116 °C. *R*_f_ = 0.15 (silica gel, hexane/ethyl acetate, 8:2). **[α]**_***D***_^**28**^ + 71.4 (c 0.327, CHCl_3_). ^**1**^**H NMR** (500 MHz, CDCl_3_) δ 13.02 (s, 1H), 7.19 (s, 1H), 6.64 (d, *J* = 2.3 Hz, 1H), 6.53 (d, *J* = 2.3 Hz, 1H), 6.04 (d, *J* = 3.8 Hz, 1H), 5.15 (d, *J* = 2.4 Hz, 1H), 4.95 (d, *J* = 2.5 Hz, 1H), 4.94 (d, *J* = 3.8 Hz, 1H), 3.98 (s, 3H), 3.91 (s, 3H), 1.62 (s, 3H), 1.39 (s, 3H). ^**13**^**C{**^**1**^**H} NMR** (125 MHz, CDCl_3_) δ: 168.5, 164.6, 162.2, 160.7, 141.5, 128.4, 119.4, 112.7, 111.9, 105.1, 99.9, 99.6, 98.8, 84.0, 82.3, 72.9, 56.4, 55.7, 26.8, 26.3. **HRMS** (DART, TOF): *m*/*z* [M + H]^+^ calcd for C_20_H_21_O_8_ 389.1236; found 389.1224.

#### (2S,3S,3aR,11bR)-2-Allyl-3,6-dihydroxy-7,9-dimethoxy-2,3,3a,11b-tetrahydro-5H-benzo[*g*]furo[3,2-*c*]isochromen-5-one (**12**)

In a flame-dried flask were added compound **10** (190.1 mg, 0.49 mmol, 1.0 equiv), allylTMS (0.38 mL, 2.45 mmol, 5.0 equiv), and DCM (4.0 mL) under a nitrogen atmosphere. The mixture was cooled at −20 °C, and TiCl_4_ (2.45 mL, 1.0 M in DCM, 2.45 mmol, 5.0 equiv) was added dropwise over 10 min. The mixture was stirred for 12 h at −20 °C and then quenched with an aqueous NaH_2_PO_4_ solution (10% m/v). The solids formed were filtered over Celite and washed several times with ethyl acetate. The solvent was removed in a high-vacuum pump, and the resulting crude was purified by flash column chromatography on silica gel (hexane/ethyl acetate, 4:6) to obtain compound **12** (113. 2 mg, 62% yield) as a pale yellow oil. *R*_f_ = 0.10 (silica gel, hexane/ethyl acetate, 7:3). **[α]**_***D***_^**24**^ + 104.7 (c 0.587, CHCl_3_). ^**1**^**H NMR** (500 MHz, CDCl_3_) δ 12.97 (s, 1H), 7.15 (s, 1H), 6.63 (d, *J* = 2.1 Hz, 1H), 6.51 (d, *J* = 2.2 Hz, 1H), 5.88–5.76 (m, 1H), 5.21–5.07 (m, 2H), 4.96 (apparent d, *J* = 2.5 Hz, 1H), 4.85 (apparent d, *J* = 2.7 Hz, 1H), 4.49 (apparent s, 1H), 4.05–3.98 (m, 1H), 3.97 (s, 3H), 3.90 (s, 3H), 3.33 (s, 1H), 2.56–2.40 (m, 2H). ^**13**^**C{**^**1**^**H} NMR** (125 MHz, CDCl_3_) δ: 169.2, 164.5, 162.2, 160.7, 141.7, 133.6, 129.9, 119.2, 118.2, 111.8, 99.9, 99.5, 98.9, 86.7, 86.0, 80.1, 73.5, 56.4, 55.7, 37.8. **HRMS** (DART, TOF) calcd for C_20_H_23_O_8_ [M + H_3_O]^+^ 391.1392, found 391.1387.

#### (2S,3aR,11bR)-6-Hydroxy-7,9-dimethoxy-2-propyl-2,3,3a,11b-tetrahydro-5*H*-benzo[*g*]furo[3,2-*c*]isochromen-5-one (***epi*****-13**)

To a solution of compound **12** (7 mg, 0.019 mmol, 1.0 equiv) in ethyl acetate (2.0 mL) was added Pd/C (0.7 mg, 10 wt %), and the mixture was stirred at room temperature for 2 h under hydrogen atmosphere in a high-pressure autoclave reactor. Afterward, the reaction was filtered over Celite and washed several times with ethyl acetate. The solvent was removed under reduced pressure, and the obtained crude was used in the next reaction without further purification.

The crude of the previous reaction was dissolved in anhydrous THF (2.0 mL) and cooled to 0 °C under a nitrogen atmosphere. Then, sodium hydride (60% in mineral oil, 1.1 mg, 0.028 mmol, 1.5 equiv) was added, and the mixture was stirred for 10 min. Afterward, CS_2_ (2.0 μL, 0.038 mmol, 2.0 equiv) was added dropwise and the mixture was allowed to warm to room temperature and stirred for an additional 10 min. Finally, MeI (2.3 μL, 00.038 mmol, 2.0 equiv) was added dropwise, and the reaction was stirred for 12 h at room temperature. Then, the reaction was quenched with water (2 mL) and extracted with ethyl acetate (3 × 3 mL). The combined organic phase was dried with Na_2_SO4, filtered, and concentrated under reduced pressure. The obtained crude product was used in the next reaction without further purification.

A solution of the crude of the previous reaction and 1,1′-azobis(cyclohexenecarbonatrile) (ACHN) (0.9 mg, 0.003 mmol, 0.15 equiv) in benzene (1.0 mL) was degassed by bubbling nitrogen. Then, Bu_3_SnH (10.1 mg, 0.135 mmol, 7.0 equiv) was added and the reaction was heated at reflux with a heating mantle for 2 h. Next, the solvent was evaporated under reduced pressure, and the resulting crude was purified by flash column chromatography on silica gel (hexane/ethyl acetate, 7:3) to obtain compound ***epi*****-13** (2.2 mg, 32% yield) as a pale oil. *R*_f_ = 0.5 (silica gel, hexane/ethyl acetate, 5:5). **[α]**_***D***_^**25**^ + 93.0 (*c* = 0.056, CHCl_3_). ^**1**^**H NMR** (500 MHz, CDCl_3_) δ 13.30 (s, 1H), 7.15 (s, 1H), 6.65 (d, *J* = 2.2 Hz, 1H), 6.53 (d, *J* = 2.2 Hz, 1H), 5.07 (dd, *J* = 5.7, 2.8 Hz, 1H), 4.65 (d, *J* = 2.7 Hz, 1H), 4.17 (dq, *J* = 8.5, 6.2 Hz, 1H), 3.99 (s, 3H), 3.91 (s, 3H), 2.59 (ddd, *J* = 14.4, 8.7, 5.8 Hz, 1H), 2.18 (dd, *J* = 14.4, 5.53, 1H), 1.78–1.66 (m, 1H), 1.55 (s, 1H), 1.49–1.38 (m, 1H), 1.39–1.31 (m, 1H), 0.91 (t, *J* = 7.4 Hz, 3H). ^**13**^**C NMR** (125 MHz, CDCl_3_) δ: 169.5, 164.5, 162.0, 160.8, 141.6, 130.7, 118.8, 112.0, 99.7, 99.4, 99.1, 81.3, 78.8, 75.3, 56.4, 55.6, 39.3, 38.3, 19.3, 14.1. Spectroscopic data agree with those reported by Brimble.^3^

### Synthesis of α,β-Unsaturated δ-Lactone **15** from γ-Lactone **16**

#### (2R,3R,5R)-5-Allyl-3-(benzyloxy)-2-((benzyloxy)methyl)tetrahydrofuran (**18**)

To a flame-dried flask were added γ-lactone **16** (2.5 g, 8.0 mmol, 1.0 equiv) and diethyl ether (150 mL) under a nitrogen atmosphere. The mixture was cooled at −78 °C and allylmagnesium bromide (12 mL, 1.0 M in diethyl ether, 12.0 mmol, 1.5 equiv) was added dropwise. The mixture was stirred for 2 h, and then the reaction was quenched with a saturated aqueous NH_4_Cl solution (50 mL) and extracted with ethyl acetate (3 × 50 mL). The combined organic phase was dried with Na_2_SO_4_, filtered, and concentrated under reduced pressure. The resulting crude was purified by flash column chromatography on silica gel (hexane/ethyl acetate, 95:5) to obtain compound **18** (270.5 mg, 10% yield) as a colorless oil. *R*_f_ = 0.15 (silica gel, hexane/ethyl acetate, 95:5). **[α]**_***D***_^**28**^ - 45.5 (c 0.406, CHCl_3_). ^**1**^**H NMR** (500 MHz, CDCl_3_) δ 7.37–7.21 (m, 10H), 5.81 (ddt, *J* = 17.2, 10.3, 7.0 Hz, 1H), 5.13–5.01 (m, 2H), 4.62 (d, *J* = 12.0 Hz, 1H), 4.58 (d, *J* = 12.2 Hz, 1H), 4.52 (d, *J* = 12.1 Hz, 1H), 4.43 (d, *J* = 12.1 Hz, 1H), 4.28 (dq, *J* = 9.6, 5.9 Hz, 1H), 4.19 (ddd, *J* = 6.7, 5.4, 4.0 Hz, 1H), 4.13 (td, *J* = 4.6, 1.4 Hz, 1H), 3.75 (dd, *J* = 9.9, 5.3 Hz, 1H), 3.69 (dd, *J* = 9.8, 6.6 Hz, 1H), 2.41 (dddt, *J* = 14.0, 7.0, 5.6, 1.3 Hz, 1H), 2.27 (dtt, *J* = 13.9, 6.9, 1.3 Hz, 1H), 2.18 (ddd, *J* = 13.3, 5.7, 1.5 Hz, 1H), 1.63 (ddd, *J* = 13.8, 9.6, 4.8 Hz, 1H). ^**13**^**C{**^**1**^**H} NMR** (125 MHz, CDCl_3_) δ: 138.5, 134.6, 128.48, 128.45, 128.0, 127.67, 127.66, 127.5, 117.3, 80.9, 79.6, 77.2, 73.6, 71.5, 69.1, 40.2, 37.3. **HRMS** (DART, TOF): *m*/*z* [M + H]^+^ calcd for C_22_H_27_O_3_ 339.1960; found 339.1973.

#### (2R,3R)-5-Allyl-1,3-bis(benzyloxy)oct-7-ene-2,5-diol (**19**)

Compound **19** was purified by flash column chromatography on silica gel (hexane/ethyl acetate, 8:2) to obtain a colorless oil (2.22 g, 70% yield). *R*_f_ = 0.2 (silica gel, hexane/ethyl acetate, 8:2). **[α]**_***D***_^**26**^ + 18.2 (c 0.307, CHCl_3_). ^**1**^**H NMR** (500 MHz, CDCl_3_) δ 7.40–7.24 (m, 10H), 5.88–5.72 (m, 2H), 5.13–5.02 (m, 4H ), 4.68 (d, *J* = 11.1 Hz, 1H), 4.59 (d, *J* = 11.1 Hz, 1H), 4.56 (d, *J* = 11.8 Hz, 1H), 4.53 (d, *J* = 12.0 Hz, 1H), 4.06–3.95 (m, 2H), 3.64 (dd, *J* = 9.7, 4.0 Hz, 1H), 3.57 (dd, *J* = 9.6, 6.6 Hz, 1H), 3.43 (s, 1H), 2.84 (d, *J* = 5.5 Hz, 1H), 2.34–2.16 (m, 4H), 1.77 (dd, *J* = 14.9, 4.8 Hz, 1H), 1.72 (dd, *J* = 15.0, 8.2 Hz, 1H). ^**13**^**C{**^**1**^**H} NMR** (125 MHz, CDCl_3_) δ: 137.8, 137.7, 134.0, 133.9, 128.71, 128.65, 128.3, 128.2, 128.1, 118.6, 118.4, 77.0, 73.7, 73.2, 72.7, 71.7, 70.9, 44.4, 44.3, 38.4. **HRMS** (DART, TOF): *m*/*z* [M + H]^+^ calcd. for C_25_H_33_O_4_ 397.2378; found 397.2368.

#### (2R,3aR,7aR)-2-Propyl-2,3,3a,7a-tetrahydro-5*H*-furo[3,2-*b*]pyran-5-one (**15**)

To a solution of compound **18** (250.0 mg, 0.73 mmol, 1.0 equiv) in ethyl acetate (5 mL) was added Pd(OH)_2_ (100 mg), and the mixture was stirred at room temperature overnight under hydrogen atmosphere in a high-pressure autoclave reactor. Afterward, the reaction was filtered over Celite and washed several times with ethyl acetate. The solvent was removed under reduced pressure, and the obtained crude was used in the next reaction without further purification.

To a solution of the crude product of the previous reaction in DCM (5.0 mL) was added (diacetoxyiodo)benzene (354.6 mg, 1.09 mmol, 1.5 equiv), followed by TEMPO (34.3 mg, 0.21 mmol, 0.3 equiv). The mixture was stirred at room temperature for 8 h, and then quenched with an aqueous Na_2_S_2_O_3_ solution (10% m/v) and extracted with DCM (3 × 5 mL). The combined organic phase was washed with a saturated aqueous NaHCO_3_ solution, dried with Na_2_SO_4_, filtered, and concentrated under reduced pressure. The obtained crude was used in the next reaction without further purification.

To a solution of the crude of the previous reaction in a mixture of EtOH/H_2_O (40:60, 10.0 mL) was added (2-methoxy-2-oxoethyl)triphenylphosphonium bromide (363.2 mg, 0.87 mmol, 1.2 equiv) followed by the portionwise addition of KOH until the reaction reached a pH of 12. The reaction was stirred at room temperature overnight and then extracted with DCM (5 × 10 mL) to remove the formed PPh_3_O. The aqueous phase was acidified with an aqueous HCl solution (10% v/v) until pH = 3 and extracted with ethyl acetate (3 × 5 mL). The combined organic phase was dried with Na_2_SO_4_, filtered, and concentrated under reduced pressure. The obtained crude was used in the next reaction without further purification.

The crude of the previous reaction and DCC (179.1 mg, 0.87 mmol, 1.2 equiv) were dissolved in anhydrous DCM under a nitrogen atmosphere, and the mixture was stirred at room temperature for 6 h. Afterward, the reaction was cooled to 0 °C, filtered over Celite, and washed with cold DCM. The solvent was evaporated under reduced pressure, and the resulting crude was purified by flash column chromatography on silica gel (hexane/ethyl acetate, 8:2) to obtain compound **15** (33.2 mg, 25% yield, four steps) as a colorless oil. *R*_f_ = 0.20 (silica gel, hexane/ethyl acetate, 8:2). **[α]**_***D***_^**28**^ – 102.7 (c 0.320, CHCl_3_). ^**1**^**H NMR** (500 MHz, CDCl_3_) δ 6.84 (dd, *J* = 9.9, 5.0 Hz, 1H), 6.11 (dd, *J* = 9.9, 1.0 Hz, 1H), 5.10 (apparent t, *J* = 4.9 Hz, 1H), 4.43 (apparent t, *J* = 4.8 Hz, 1H), 4.21 (ddd, *J* = 10.2, 6.8, 5.0 Hz, 1H), 2.41 (dd, *J* = 13.7, 5.2 Hz, 1H), 1.91 (ddd, *J* = 13.5, 9.9, 5.3 Hz, 1H), 1.62 (dddd, *J* = 11.9, 9.1, 7.0, 5.1 Hz, 1H), 1.54–1.27 (m, 3H), 0.93 (t, *J* = 7.2 Hz, 3H). ^**13**^**C{**^**1**^**H} NMR** (125 MHz, CDCl_3_) δ: 162.1, 141.2, 122.8, 81.0, 78.8, 68.9, 40.3, 37.6, 19.3, 14.2. **HRMS** (DART, TOF): *m*/*z* [M + H]^+^ calcd for C_10_H_15_O_3_ 183.1021; found 183.1021.

#### (3*R*,4*R*)-3,5-Bis(benzyloxy)-4-hydroxy-*N*-methoxy-*N*-methylpentanamide (**20**)

To a flame-dried flask were added amine hydrochloride (0.9363 g, 9.60 mmol, 3.0 equiv) and THF (4.0 mL) under a nitrogen atmosphere. The resulting suspension was cooled at 0 °C, and DIBAL (1 M in hexane, 8.96 mL, 8.96 mmol, 2.8 equiv) was added dropwise. The reaction was stirred at 0 °C for 10 min, then allowed to warm to room temperature, and stirred for an additional 2 h to generate the DIBAL-H-NMe(OMe) HCl complex. **Note:** Water was removed from *N*,*O*-dimethylhydroxylamine hydrochloride by azeotropic distillation with toluene. To a solution of lactone **16** (1.00 g, 3.20 mmol, 1.0 equiv) in THF under a nitrogen atmosphere was added dropwise the previously formed DIBAL-H-NMe(OMe) HCl complex. The mixture was stirred at room temperature for 4 h and then cooled at 0 °C, quenched with an aqueous Rochelle’s salt solution (10% m/v), and stirred at room temperature until two phases were observed. The crude was then extracted with ethyl acetate (3 × 20 mL), and the combined organic phase was dried with Na_2_SO_4_, filtered, and concentrated under reduced pressure. The resulting crude was purified by flash column chromatography on silica gel (hexane/ethyl acetate, 6:4) to obtain compound **20** (944.2 mg, 79% yield) as a pale yellow oil. *R*_f_ = 0.4 (silica gel, hexane/ethyl acetate, 5:5). **[α]**_***D***_^**28**^ –18.7 (c 1.873, CHCl_3_). ^**1**^**H NMR** (500 MHz, CDCl_3_) δ 7.30–7.16 (m, 10H), 4.59 (d, *J* = 11.5 Hz, 1H), 4.50–4.41 (m, 3H), 4.10–4.06 (m, 1H), 3.79 (br, 1H), 3.56 (s, 3H), 3.53–3.45 (m, 2H), 3.10 (s, 3H), 2.79 (dd, *J* = 16.3, 6.7 Hz, 1H), 2.74–2.64 (m, 1H). ^**13**^**C{**^**1**^**H} NMR** (126 MHz, CDCl_3_) δ: 172.3, 138.3, 138.1, 128.4, 128.4, 128.1, 127.9, 127.8, 127.8, 75.9, 73.5, 73.2, 72.0, 71.4, 61.4, 33.9, 32.2. **HRMS** (DART, TOF): *m*/*z* [M + H]^+^ calcd for C_21_H_28_NO_5_ 374.1967; found 374.1972.

#### (2R,3R,5R)-3-(Benzyloxy)-2-((benzyloxy)methyl)-5-propyltetrahydrofuran (**21**)

To a flame-dried flask were added Weinreb amide **20** (920.0 mg, 2.45 mmol, 1.0 equiv) and THF (16.0 mL) under a nitrogen atmosphere. The mixture was cooled to 0 °C, and propylmagnesium chloride (4.30 mL, 8.63 mmol, 3.5 equiv) was added dropwise. The mixture was stirred overnight, and then, the reaction was quenched with a saturated aqueous NH_4_Cl solution (15 mL) and extracted with ethyl acetate (3 × 15 mL). The combined organic extract was dried with Na_2_SO_4_, filtered, and concentrated under reduced pressure. The obtained crude was used in the next reaction without further purification.

To a solution of the crude of the previous reaction and Et_3_SiH (1.96 mL, 12.30 mmol, 5.0 equiv) in anhydrous DCM (16.0 mL) under nitrogen atmosphere at −40 °C was added dropwise BF_3_·OEt_2_ (1.51 mL, 12.30 mmol, 5.0 equiv). The reaction was stirred for 12 h at this temperature, and then quenched with an aqueous saturated NaHCO_3_ solution and extracted with DCM (3 × 10 mL). The combined organic phase was dried with Na_2_SO_4_, filtered, and concentrated under reduced pressure. The resulting crude product was purified by flash column chromatography on silica gel (hexane/ethyl acetate, 95:5) to obtain compound **21** (333.5 mg, 40% yield, two steps) as a colorless oil. *R*_f_ = 0.6 (silica gel, hexane/ethyl acetate, 9:1). **[α]**_***D***_^**28**^ –46.3 (c 1.053, CHCl_3_). ^**1**^**H NMR** (500 MHz, CDCl_3_) δ 7.29–7.13 (m, 10H), 4.54 (d, *J* = 12.1 Hz, 1H), 4.51 (d, *J* = 12.1 Hz, 1H), 4.45 (d, *J* = 12.1 Hz, 1H), 4.36 (d, *J* = 12.1 Hz, 1H), 4.15–4.02 (m, 3H), 3.68 (dd, *J* = 9.8, 5.4 Hz, 1H), 3.60 (dd, *J* = 9.8, 6.5 Hz, 1H), 2.11 (ddd, *J* = 13.2, 5.6, 1.5 Hz, 1H), 1.60–1.50 (m, 1H), 1.48 (ddd, *J* = 13.7, 9.5, 4.7 Hz, 1H), 1.41–1.20 (m, 3H), 0.84 (t, *J* = 7.1 Hz, 3H). ^**13**^**C{**^**1**^**H} NMR** (125 MHz, CDCl_3_) δ: 138.6 (2C), 128.43, 128.40, 127.9, 127.60, 127.58, 127.5, 80.5, 79.7, 77.7, 73.5, 71.5, 69.1, 38.2, 38.0, 19.3, 14.3. **HRMS** (DART, TOF): *m*/*z* [M + H]^+^ calcd for C_22_H_29_O_3_ 341.2116; found 341.2101.

#### (2R,3aR,7aR)-2-Propyl-2,3,3a,7a-tetrahydro-5*H*-furo[3,2-*b*]pyran-5-one (**15**)

Lactone **15** was prepared from **21** (280.0 mg, 0.82 mmol, 1.0 equiv), following the same procedure as from **18** to **15**, to give 45 mg (30% yield) of compound **15** as a colorless oil after purification by flash column chromatography on silica gel (hexane/ethyl acetate, 8:2) *R*_f_ = 0.20 (silica gel, hexane/ethyl acetate, 8:2). Spectroscopic data are identical with those obtained previously (**18** to **15**).

### Preparation of Compound **15** from Lactone **22**

#### (2R,3R,5R)-5-Propyl-2-vinyltetrahydrofuran-3-ol (**23**)

To a flame-dried flask were added *N*,*O*-dimethylhydroxylamine hydrochloride (661.92 mg, 6.78 mmol, 3.0 equiv) and THF (3 mL) under a nitrogen atmosphere. The resulting suspension was cooled at 0 °C, and DIBAL-H (1 M in hexane, 6.33 mL, 6.33 mmol, 2.8 equiv) was added dropwise. The reaction was stirred at 0 °C for 10 min, then allowed to warm to room temperature, and stirred for an additional 2 h to generate the DIBAL-H-NMe(OMe) HCl complex. To a solution of lactone **22** (548.3 mg, 2.26 mmol, 1.0 equiv) in THF (8 mL) was added dropwise the previously formed DIBAL-H-NMe(OMe) HCl complex. The mixture was stirred at room temperature for 4 h and then cooled at 0 °C, quenched with an aqueous Rochelle’s salt solution (10% m/v), and stirred at room temperature until two phases were observed. The crude was then extracted with ethyl acetate (3 × 15 mL), and the combined organic phase was dried with Na_2_SO_4_, filtered, and concentrated under reduced pressure. The obtained crude was used in the next reaction without further purification. **Note:** Water was removed from *N,O*-dimethylhydroxylamine hydrochloride by azeotropic distillation with toluene.

The crude of the previous reaction was dissolved in DCM (11.3 mL) under a nitrogen atmosphere, and the mixture was cooled at −20 °C. Then, imidazole (308 mg, 4.52 mL, 2.0 equiv) was added to the solution followed, after 15 min, by the addition of TMSCl (0.57 mL, 4.52 mL, 2.0 equiv). The reaction was allowed to stir for 5 h and then quenched with an aqueous saturated NaHCO_3_ solution (8 mL) and extracted with DCM (3 × 10 mL). The combined organic phase was dried with Na_2_SO_4_, filtered, and concentrated under reduced pressure. The obtained crude was used in the next reaction without further purification.

To a solution of the crude of the previous reaction in THF (15 mL) under a nitrogen atmosphere at −20 °C was added dropwise propylmagnesium chloride (1.69 mL, 3.39 mmol, 1.5 equiv). After the mixture was stirred for 2 h, another portion of propylmagnesium chloride (1.69 mL, 3.39 mmol, 1.5 equiv) was added dropwise, and the reaction mixture was allowed to warm to 0 °C and stirred for an additional 1 h. Afterward, the reaction was quenched with a saturated aqueous NH_4_Cl solution (20 mL) and extracted with ethyl acetate (3 × 30 mL). The combined organic phase was dried with Na_2_SO_4_, filtered, and concentrated under reduced pressure. The obtained crude was used in the next reaction without further purification.

To a solution of the crude of the previous reaction in a mixture of MeOH/DCM (1:1, 28 mL) at 0 °C was added CSA (105 mg, 0.45 mmol, 0.2 equiv). The reaction was quenched with Et_3_N (0.5 mL) after stirring for 20 min, and then the solvents were removed by evaporation. The crude was then redissolved in ethyl acetate, washed with water, and extracted with ethyl acetate (3 × 15 mL). The combined organic phase was dried with Na_2_SO_4_, filtered, and concentrated under reduced pressure. The obtained crude was used in the next reaction without further purification.

To a solution of the crude of the previous reaction and Et_3_SiH (1.80 mL, 11.31 mmol, 5.0 equiv) in DCM (14.7 mL) under a nitrogen atmosphere at −65 °C was added dropwise BF_3_·OEt_2_ (1.14 mL, 11.31 mmol, 5.0 equiv). The reaction was stirred overnight and then quenched with an aqueous saturated NaHCO_3_ solution (10 mL) and extracted with DCM (3 × 10 mL). The combined organic extract was dried with Na_2_SO_4_, filtered, and concentrated under reduced pressure. The obtained crude was used in the next reaction without further purification.

To a solution of the crude of the previous reaction in THF (15 mL) at 0 °C was added dropwise TBAF (1.0 M in THF, 3.39 mL, 1.5 equiv), and the mixture was stirred for 10 min. Then, the reaction was allowed to warm to room temperature and stirred for an additional 5 h. Upon completion, the reaction was quenched with an aqueous saturated NaHCO_3_ solution (10 mL) and extracted with ethyl acetate (3 × 15 mL). The combined organic phase was dried with Na_2_SO_4_, filtered, and concentrated under reduced pressure. The resulting crude was purified by flash column chromatography on silica gel (hexane/ethyl acetate = 9:1) to obtain the compound **23** (95.4 mg, 27% yield, six steps) as an inseparable diastereomeric mixture (4:1 *d.r.*). *R*_f_ = 0.3 (silica gel, hexane/ethyl acetate = 8:2). **[α]**_***D***_^**26**^ –34.6 (c 0.553, CHCl_3_). NMR data are reported for the major diastereomer: ^**1**^**H NMR** (500 MHz, CDCl_3_) δ 5.91 (ddd, *J* = 17.6, 10.6, 5.7 Hz, 1H), 5.45 (dd, *J* = 17.4, 1.7 Hz, 1H), 5.35 (dd, *J* = 10.6, 1.6 Hz, 1H), 4.47–4.40 (m, 1H), 4.33–4.21 (m, 2H), 2.14 (ddd, *J* = 13.1, 5.8, 1.2 Hz, 1H), 1.72 (ddd, *J* = 13.0, 9.7, 4.5 Hz, 1H), 1.67–1.58 (m, 2H), 1.51–1.40 (m, 2H), 0.93 (t, *J* = 7.2 Hz, 3H). ^**13**^**C{**^**1**^**H} NMR** (125 MHz, CDCl_3_) δ: 134.0, 118.6, 83.0, 78.0, 74.0, 41.4, 38.4, 19.3, 14.8. **HRMS** (DART, TOF): *m*/*z* [M + H]^+^ calcd for C_9_H_17_O_2_ 157.1228; found 157.1234.

#### (2R,3R,5R)-5-Propyl-2-vinyltetrahydrofuran-3-yl acrylate (**24**)

To a flame-dried flask were added acrylic acid (0.12 mL, 1.83 mmol, 3.0 equiv), DCM (2.5 mL), and DMF (5 drops) under a nitrogen atmosphere. Then, oxalyl chloride (0.15 mL, 1.71 mmol, 2.8 equiv) was added dropwise, and the reaction was stirred at room temperature for 3 h. Separately, to a flame-dried flask were added the alcohol **23** (95.4 mg, 0.61 mmol, 1.0 equiv), DIPEA (0.64 mL, 3.66 mmol, 6 equiv), and DCM (2.5 mL) under a nitrogen atmosphere. The previous solution of the acyl chloride was added dropwise to the solution of the alcohol at 0 °C. After 20 min, the reaction mixture was allowed to warm to rt and stirred overnight. Then, the reaction was quenched with an aqueous saturated NaHCO_3_ solution (10 mL), stirred for 20 min, and extracted with DCM (3 × 10 mL). The combined organic phase was dried with Na_2_SO_4_, filtered, and evaporated under reduced pressure. The residue was purified by flash column chromatography on silica gel (hexane/ethyl acetate, 95:5) to obtain compound **24** (105.3 mg, 82%) as an inseparable diastereomeric mixture (3:1 *d.r.*). **[α]**_***D***_^**27**^ – 14.6 (c 0.467, CHCl_3_). *R*_f_ = 0.6 (TLC, hexane/ethyl acetate = 9:1). NMR data are reported for the major diastereomer: ^**1**^**H NMR** (500 MHz, CDCl_3_) δ 6.41 (apparent d, *J* = 17.3 Hz, 1H), 6.20–6.04 (m, 1H), 5.88–5.76 (m, 2H), 5.48–5.42 (m, 1H), 5.34 (apparent d, *J* = 17.2 Hz, 1H), 5.20 (apparent d, *J* = 10.9 Hz, 1H), 4.52 (apparent t, *J* = 5.4 Hz, 1H), 4.31–4.20 (m, 1H), 2.18 (dd, *J* = 13.7, 5.8 Hz, 1H), 1.92–1.84 (m, 1H), 1.72–1.59 (m, 2H), 1.51–1.41 (m, 2H), 0.94 (t, *J* = 7.1 Hz, 3H). ^**13**^**C{**^**1**^**H} NMR** (125 MHz, CDCl_3_) δ: 165.6, 133.3, 131.3, 128.4, 118.3, 81.9, 78.0, 76.5, 39.2, 38.2, 19.3, 14.3. **HRMS** (DART, TOF): *m*/*z* [M + H]^+^ calcd for C_12_H_19_O_3_ 211.1334; found 211.1332.

#### (2R,3aR,7aR)-2-Propyl-2,3,3a,7a-tetrahydro-5*H*-furo[3,2-*b*]pyran-5-one (**15**)

To a flame-dried flask were added compound **24** (105.3 mg, 0.50 mmol, 1.0 equiv), Grubbs second-generation catalyst (21.2 mg, 0.05 equiv), and DCM (0.025 M) under a nitrogen atmosphere, and the mixture was stirred overnight at rt. Then, the solvent was evaporated under reduced pressure, and the resulting crude product was purified by flash column chromatography on silica gel (hexane/ethyl acetate = 9:1) to obtain compound **15** as a colorless oil (72.0 mg, 80% yield). *R*_f_ = 0.2 (silica gel, hexane/ethyl acetate = 8:2). Spectroscopic data are identical to those obtained previously (**18** to **15**).

#### (2R,3aR,11bR)-7,9-Dimethoxy-2-propyl-2,3,3a,11b-tetrahydro-5*H*-benzo[*g*]furo[3,2-*c*]isochromen-5-one (**25**)

Compound **25** was purified by flash column chromatography on silica gel (hexane/ethyl acetate, 8:2) to obtain a white solid. (3.6 mg, 15%). mp 124–126 °C *R*_f_ = 0.35 (silica gel, hexane:ethyl acetate, 7:3). **[α]**_***D***_^**25**^ – 99.1 (c 0.280, CHCl_3_). ^**1**^**H NMR** (500 MHz, CDCl_3_) δ 9.07 (s, 1H), 7.71 (s, 1H), 6.72 (d, *J* = 2.2 Hz, 1H), 6.51 (d, *J* = 2.1 Hz, 1H), 5.18 (apparent t, *J* = 3.4 Hz, 1H), 4.97 (apparent d, *J* = 2.7 Hz, 1H), 4.43 (dq, *J* = 9.5, 6.1 Hz, 1H), 3.97 (s, 3H), 3.92 (s, 3H), 2.63 (ddd, *J* = 13.7, 6.0, 1.0 Hz, 1H), 2.03 (ddd, *J* = 13.8, 9.6, 4.2 Hz, 1H), 1.78–1.68 (m, 1H), 1.64–1.51 (m, 1H), 1.53–1.31 (m, 2H), 0.95 (t, *J* = 7.3 Hz, 3H). ^**13**^**C{**^**1**^**H} NMR** (125 MHz, CDCl_3_) δ: 164.6, 161.4, 158.0, 138.3, 132.4, 127.5, 127.2, 122.1, 117.7, 98.9, 97.9, 81.4, 79.4, 73.8, 55.8, 55.7, 40.5, 38.5, 19.3, 14.2. **HRMS** (DART, TOF): *m*/*z* [M + H]^+^ calcd for C_20_H_23_O_5_ 343.1545; found 343.1544.

#### (2R,3aR,5aR,6R,11aR,11bR)-6-Hydroxy-7,9-dimethoxy-2-propyl-2,3,3a,5a,6,11,11a,11b-octahydro-5*H*-benzo[*g*]furo[3,2-*c*]isochromen-5-one (**26**)

In a flame-dried microwave tube, lactone **15** (35.7 mg, 0.19 mmol, 1.0 equiv) and benzocyclobutenol **7** (68.4 mg, 0.38 mmol, 2.0 equiv) were dissolved in a mixture of toluene/DMPU (2:1, 0.38 mL, 0.5 M) under a nitrogen atmosphere, and the mixture was degassed by bubbling nitrogen for 10 min. The tube was sealed, and the reaction was heated at 120 °C for 1 h under microwave irradiation. Then, the reaction was allowed to cool to room temperature, another portion of benzocyclobutenol **7** was added (68.4 mg, 0.38 mmol, 2.0 equiv), and the reaction was heated at 120 °C under microwave irradiation for an additional 1 h. Afterward, the reaction was diluted with ethyl acetate (5 mL) and washed with a saturated aqueous CuSO_4_ solution (5 × 3 mL). The organic phase was dried with Na_2_SO_4_, filtered, and concentrated under reduced pressure. The resulting crude was purified by flash column chromatography on silica gel (hexane/ethyl acetate, 8:2) to obtain compound **26** (35.7 mg, 52% yield) as a colorless oil. *R*_f_ = 0.25 (silica gel, hexane/ethyl acetate, 7:3). **[α]**_**D**_^**25**^ + 8.36 (c 0.293, CHCl_3_). ^**1**^**H NMR** (500 MHz, CDCl_3_) δ 6.34 (d, *J* = 2.3 Hz, 1H), 6.30 (d, *J* = 2.3 Hz, 1H), 5.28 (dd, *J* = 6.2, 2.5 Hz, 1H), 5.24 (apparent t, *J* = 6.8 Hz, 1H), 4.31 (dd, *J* = 9.7, 6.4 Hz, 1H), 4.06 (dq, *J* = 10.9, 5.7 Hz, 1H), 3.84 (s, 3H), 3.78 (s, 3H), 3.10 (dd, *J* = 17.2, 1.8 Hz, 1H), 3.02 (apparent t, *J* = 5.6 Hz, 1H), 2.94 (br, 1H), 2.87 (dd, *J* = 17.2, 5.7 Hz, 1H), 2.38–2.30 (m, 1H), 2.25 (dd, *J* = 13.6, 4.7 Hz, 1H), 1.76 (ddd, *J* = 13.5, 10.1, 7.2 Hz, 1H), 1.69–1.58 (m, 2H), 1.54–1.40 (m, 2H), 1.37–1.32 (m, 1H), 0.93 (t, *J* = 7.0 Hz, 3H). ^**13**^**C{**^**1**^**H} NMR** (125 MHz, CDCl_3_) δ: 174.0, 160.6, 158.7, 135.4, 118.1, 105.1, 97.1, 82.4, 76.5, 76.4, 62.1, 55.6, 55.5, 44.1, 40.2, 36.6, 31.7, 30.9, 19.4, 14.3. **HRMS** (DART, TOF): *m*/*z* [M – OH]^+^ calcd for C_20_H_25_O_5_ 345.1702; found 345.1707.

#### (2R,3aR,11aR,11bR)-7,9-Dimethoxy-2-propyl-2,3,3a,11,11a,11b-hexahydro-5*H*-benzo[*g*]furo[3,2-*c*]isochromen-5-one (**27**)

Compound **27** was purified by flash column chromatography on silica gel (hexane/ethyl acetate, 8:2) to obtain a white solid. (1.2 mg, 4.2%). mp 116–119 °C *R*_f_ = 0.30 (silica gel, hexane/ethyl acetate, 7:3). **[α]**_***D***_^**24**^ – 97.5 (c 0.667, CHCl_3_). ^**1**^**H NMR** (500 MHz, CDCl_3_) δ 7.97 (d, *J* = 2.5 Hz, 1H), 6.33 (d, *J* = 2.2 Hz, 1H), 6.28 (d, *J* = 2.3 Hz, 1H), 4.83 (ddd, *J* = 5.6, 3.6, 2.1 Hz, 1H), 4.26 (dq, *J* = 8.7, 6.3 Hz, 1H), 4.00 (apparent t, *J* = 3.5 Hz, 1H), 3.82 (s, 3H), 3.81 (s, 3H), 3.05–2.92 (m, 2H), 2.75 (apparent t, *J* = 14.1 Hz, 1H), 2.41 (ddd, *J* = 13.6, 6.3, 2.2 Hz, 1H), 1.87 (ddd, *J* = 13.7, 8.7, 5.3 Hz, 1H), 1.62 (dddt, *J* = 11.7, 9.2, 6.6, 5.1 Hz, 1H), 1.53–1.27 (m, 3H), 0.94 (t, *J* = 7.2 Hz, 3H). ^**13**^**C{**^**1**^**H} NMR** (125 MHz, CDCl_3_) δ: 167.4, 162.6, 158.7, 139.1, 134.0, 118.9, 114.9, 105.3, 96.5, 79.1, 79.1, 77.3, 55.7, 55.6, 39.4, 38.1, 34.6, 34.5, 19.3, 14.2. **HRMS** (DART, TOF): *m*/*z* [M + H]^+^ calcd for C_20_H_25_O_5_ 345.1702; found 345.1702.

#### (2R,3aR,11bR)-6-Hydroxy-7,9-dimethoxy-2-propyl-2,3,3a,11b-tetrahydro-5*H*-benzo[*g*]furo[3,2-*c*]isochromen-5-one (**13**)

To a solution of Diels–Alder adduct **26** (30.1 mg, 0.083 mmol, 1.0 equiv) in toluene (0.5 mL) was added DDQ (50 mg, 0.236 mmol, 1.0 equiv) dropwise dissolved in toluene (0.5 mL) for 2 h at 0 °C. Then, the reaction was allowed to warm to room temperature and stirred for an additional 12 h. Afterward, the reaction was filtered over Celite, and the filtrate was washed several times with toluene. The solvent was evaporated in a high-vacuum pump, the crude was redissolved in ethyl acetate (10 mL) and washed with an aqueous saturated NaHCO_3_ solution (5 × 5 mL). The organic phase was dried with Na_2_SO_4_, filtered, and concentrated under reduced pressure. The resulting crude product was purified by flash column chromatography on silica gel (hexane/ethyl acetate, 8:2) to obtain compound **13** (17.1 mg, 58% yield) as a white solid. mp 124–126 °C. *R*_f_ = 0.4 (silica gel, hexane/ethyl acetate, 7:3). **[α]**_***D***_^**28**^ + 64.0 (c 0.100, CHCl_3_). ^**1**^**H NMR** (500 MHz, CDCl_3_) δ 13.28 (s, 1H), 7.14 (s, 1H), 6.64 (d, *J* = 1.9 Hz, 1H), 6.52 (d, *J* = 1.8 Hz, 2H), 5.15 (apparent t, *J* = 3.8 Hz, 1H), 4.88 (apparent t, *J* = 2.0 Hz, 1H), 4.42 (dq, *J* = 12.5, 6.4 Hz, 1H), 3.98 (s, 3H), 3.91 (d, *J* = 1.4 Hz, 3H), 2.63 (dd, *J* = 13.9, 6.0 Hz, 1H), 2.01 (ddd, *J* = 13.7, 9.4, 4.1 Hz, 1H), 1.71–1.65 (m, 1H), 1.62–1.32 (m, 4H), 0.95 (t, *J* = 7.3 Hz, 3H). ^**13**^**C{**^**1**^**H} NMR** (125 MHz, CDCl_3_) δ: 169.8, 164.5, 162.0, 160.7, 141.7, 131.2, 118.7, 111.9, 99.7, 99.3, 98.6, 81.9, 79.1, 74.0, 56.4, 55.6, 40.3, 38.5, 19.3, 14.2. **HRMS** (DART, TOF): *m*/*z* [M + H]^+^calcd for C_20_H_23_O_6_ 359.1494: found 359.1492. Spectroscopic data agree with those reported by Brimble.^3^

## Data Availability

The data underlying this study are available in the published article and its Supporting Information.

## Supporting Information Available

^1^H and ^13^C and 2D NMR spectra for new and relevant compounds (PDF)
