# Supplementary material for: En Route to Furan-Fused Naphthopyrones: Formal Synthesis of the (+)-Lasionectrin and Its C12-Epimer
Source: J Org Chem. 2023 Dec 4;88(24):17409–19. doi: 10.1021/acs.joc.3c02231 (PMC10729057; doi:10.1021/acs.joc.3c02231)

Supporting Information

En Route to Furan-Fused Naphthopyrones: Formal Synthesis of the (+)-Lasionectrin and its C12-Epimer

Pedro López-Mendoza,[1] Luis F. Porras-Santos,[1] *Alvano Pérez-Bautista,* [1] *Leticia Quintero,*[1]*Jocelyn Bautista-Nava,*[1] *David, F. León-Rayo*[2], *Alejandro Cordero-Vargas,*[2*] *and Fernando Sartillo-Piscil* [1,*]

[1]Centro de Investigación de la Facultad de Ciencias Químicas, Benemérita Universidad Autónoma de Puebla (BUAP), 14 Sur Esq. San Claudio, Col. San Manuel, 72570, Puebla, México.

[2]Instituto de Química, Universidad Nacional Autónoma de México, Circuito Exterior Ciudad Universitaria, 04510, CDMX, México.

fernando.sartillo@correo.buap.mx. Fax: +52222 2454972; Tel: +52 222 2955500 ext. 7391.

acordero@unam.mx

Content

^{1}H NMR spectrum of compound **7** (300 MHz, $CDCl_3$): S4
$^{13}C\{^{1}H\}$ NMR of compound **7** (75MHz, $CDCl_3$): S5
^{1}H NMR spectrum of compound **9** (500 MHz, $CDCl_3$): S6
$^{13}C\{^{1}H\}$ NMR spectrum of compound **9** (125 MHz, $CDCl_3$): S7
COSY NMR spectrum of compound **9** (500 MHz, $CDCl_3$): S8
HSQC NMR spectrum of compound **9** (500 MHz, $CDCl_3$): S9
NOESY NMR spectrum of compound **9** (500 MHz, $CDCl_3$): S10
^{1}H NMR spectrum of compound **11** (500 MHz, $CDCl_3$): S11
$^{13}C\{^{1}H\}$ NMR spectrum of compound **11** (125 MHz, $CDCl_3$): S12
^{1}H NMR spectrum of compound **10** (500 MHz, $CDCl_3$): S13
$^{13}C\{^{1}H\}$ NMR spectrum of compound **10** (125 MHz, $CDCl_3$): S14
^{1}H NMR spectrum of compound **12** (500 MHz): S15
$^{13}C\{^{1}H\}$ NMR spectrum of compound **12** (125 MHz, $CDCl_3$): S16
COSY NMR spectrum of compound **12** (500 MHz, $CDCl_3$): S17
HSQC NMR spectrum of compound **12** (500 MHz, $CDCl_3$): S18
HMBC NMR spectrum of compound **12** (500 MHz, $CDCl_3$): S19
NOESY NMR spectrum of compound **12** (500 MHz, $CDCl_3$): S20
^{1}H NMR spectrum of compound ***epi*-13** (500 MHz, $CDCl_3$): S21
$^{13}C\{^{1}H\}$ NMR spectrum of compound ***epi*-13** (125 MHz, $CDCl_3$): S22
^{1}H NMR spectrum of compound **16** (500 MHz, $CDCl_3$): S23
$^{13}C\{^{1}H\}$ NMR spectrum of compound **16** (125 MHz, $CDCl_3$): S24
^{1}H NMR spectrum of compound **18** (500 MHz, $CDCl_3$): S25
$^{13}C\{^{1}H\}$ NMR spectrum of compound **18** (125 MHz, $CDCl_3$): S26
^{1}H NMR spectrum of compound **19** (500 MHz, $CDCl_3$): S27
$^{13}C\{^{1}H\}$ NMR spectrum of compound **19** (125 MHz, $CDCl_3$): S28
^{1}H NMR spectrum of compound **20** (500 MHz, $CDCl_3$): S29
$^{13}C\{^{1}H\}$ NMR spectrum of compound **20** (125 MHz, $CDCl_3$): S30
^{1}H NMR spectrum of compound **21** (500 MHz, $CDCl_3$): S31
$^{13}C\{^{1}H\}$ NMR spectrum of compound **21** (125 MHz, $CDCl_3$): S32
^{1}H NMR spectra of compound **15** (500 MHz, $CDCl_3$): S33
$^{13}C\{^{1}H\}$ NMR spectrum of compound **15** (125 MHz, $CDCl_3$): S34

^{1}H NMR spectrum of compound **23** (500 MHz, $CDCl_3$): S35
$^{13}C\{^{1}H\}$ NMR spectrum of compound **23** (125 MHz, $CDCl_3$): S36
^{1}H NMR spectrum of compound **24** (500 MHz, $CDCl_3$): S37
$^{13}C\{^{1}H\}$ NMR spectrum of compound **24** (125 MHz, $CDCl_3$): S38
^{1}H NMR spectrum of compound **25** (500 MHz, $CDCl_3$): S39
$^{13}C\{^{1}H\}$ NMR spectrum of **25** (125 MHz, $CDCl_3$): S40
^{1}H NMR spectrum of compound **26** (500 MHz, $CDCl_3$): S41
$^{13}C\{^{1}H\}$ NMR spectrum of compound **26** (125 MHz, $CDCl_3$): S42
^{1}H NMR spectrum of compound **27** (500 MHz, $CDCl_3$): S43
$^{13}C\{^{1}H\}$ NMR spectrum of compound **27** (125 MHz, $CDCl_3$): S44
^{1}H NMR spectrum of compound **13** (500 MHz, $CDCl_3$): S45
$^{13}C\{^{1}H\}$ NMR spectrum of compound **13** (125 MHz, $CDCl_3$): S46

^{1}H NMR spectrum of compound 7 (300 MHz, $CDCl_3$):

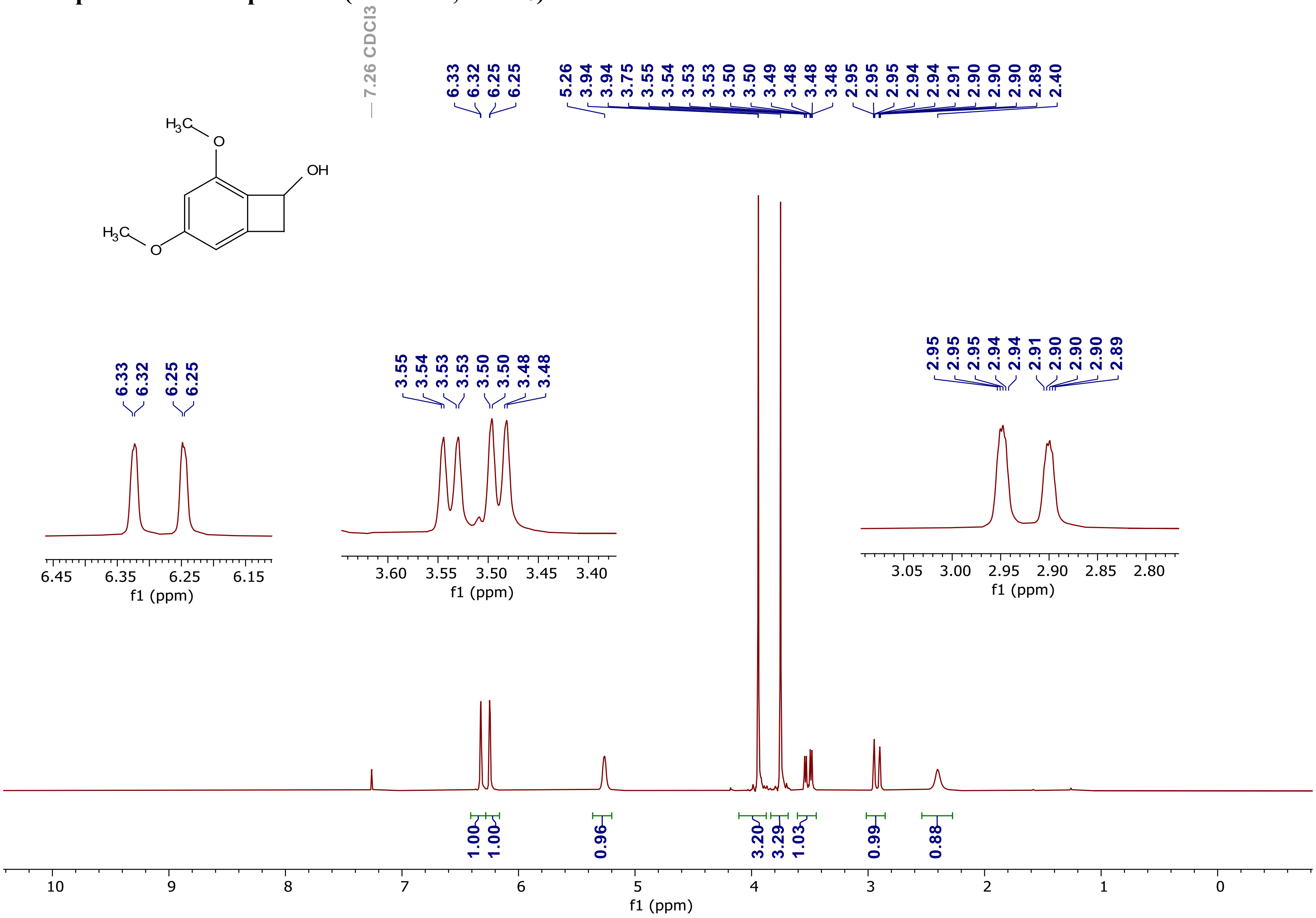

^{13}C{^{1}H} NMR of compound 7 (75MHz, CDCl3):

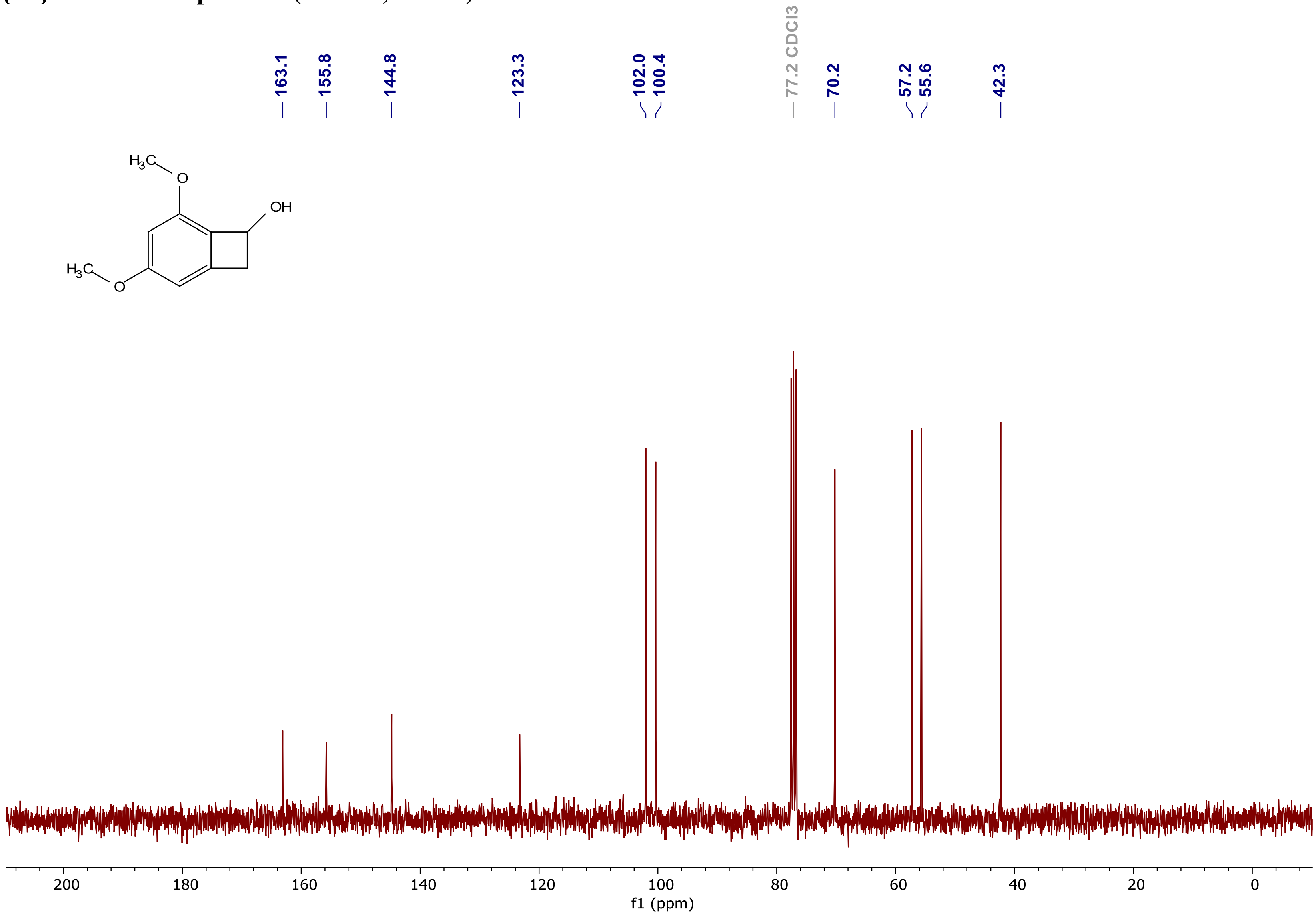

^{1}H NMR spectrum of compound 9 (500 MHz, CDCl3):

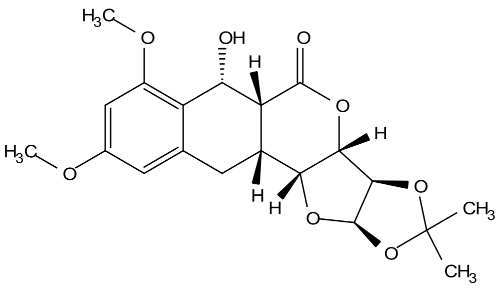

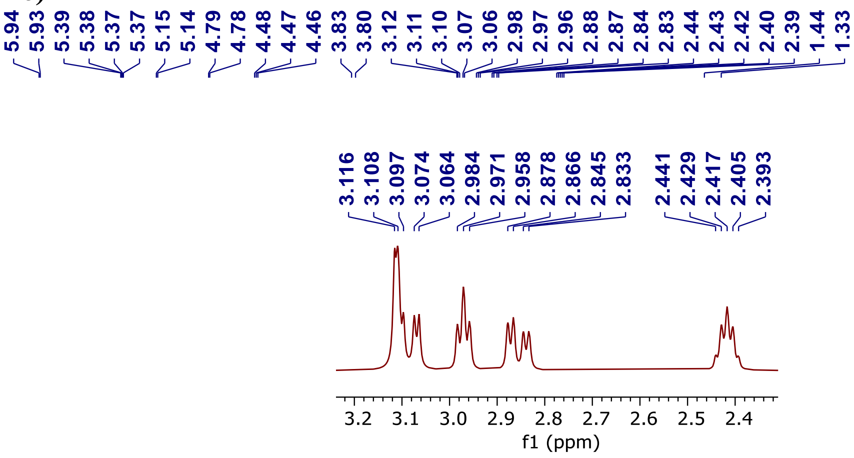

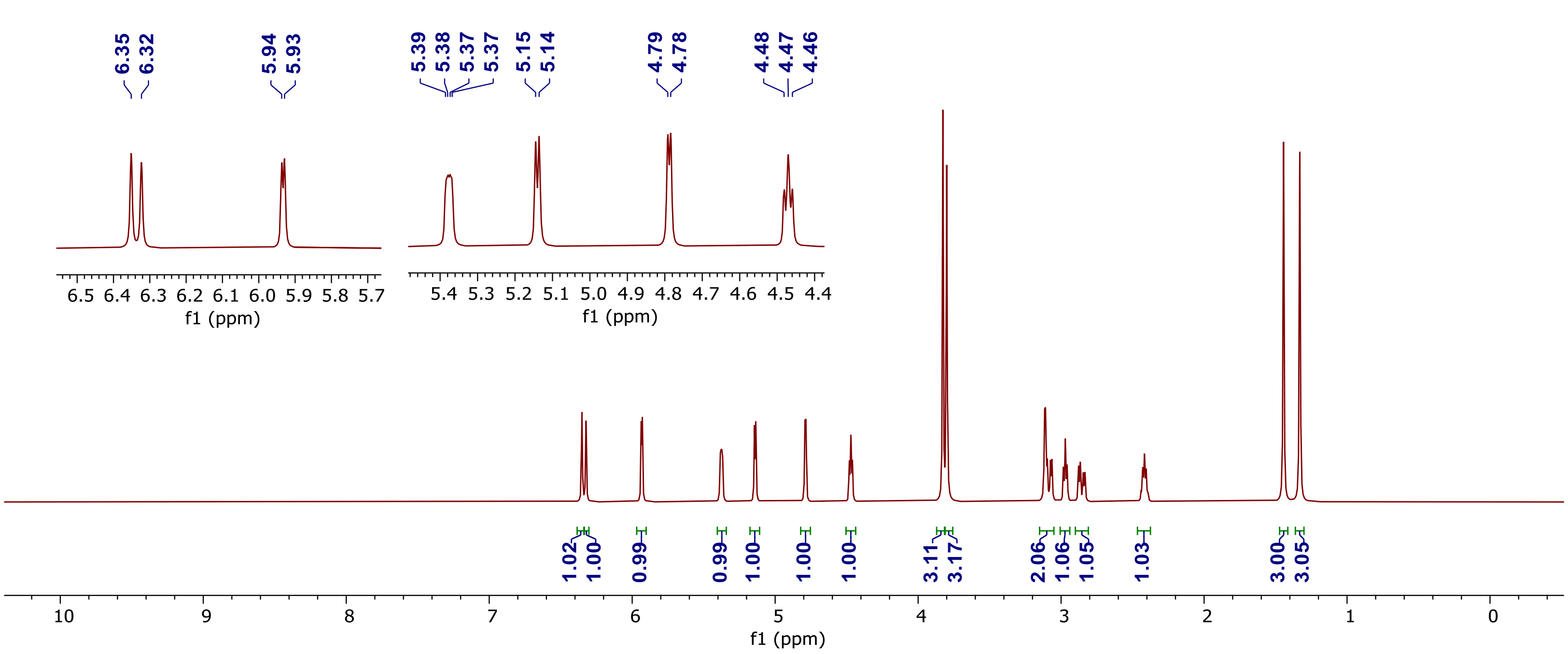

$^{13}C\{^{1}H\}$ NMR spectrum of compound 9 (125 MHz, $CDCl_3$):

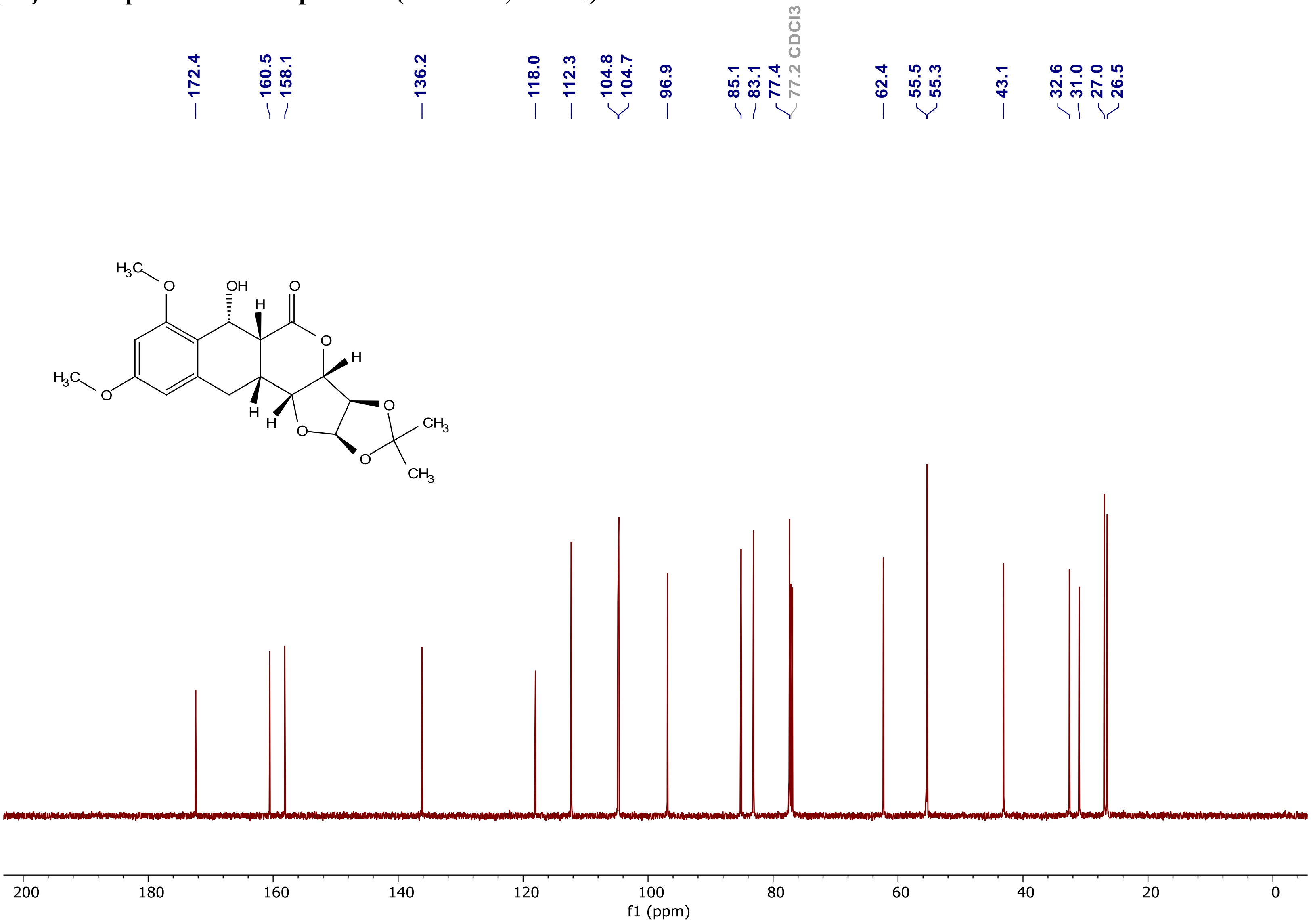

COSY NMR spectrum of compound 9 (500 MHz, $CDCl_3$):

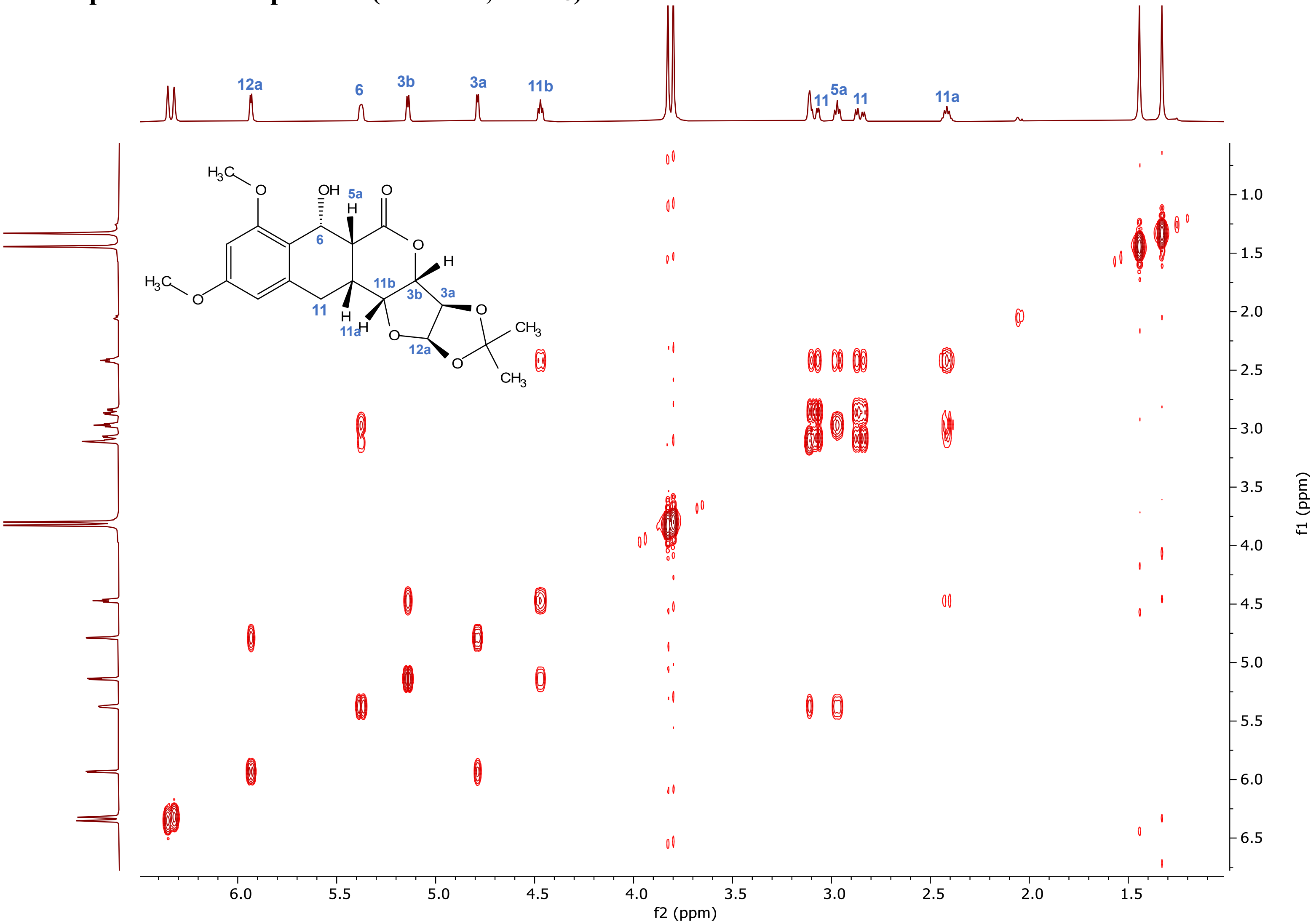

HSQC NMR spectrum of compound 9 (500 MHz, $CDCl_3$):

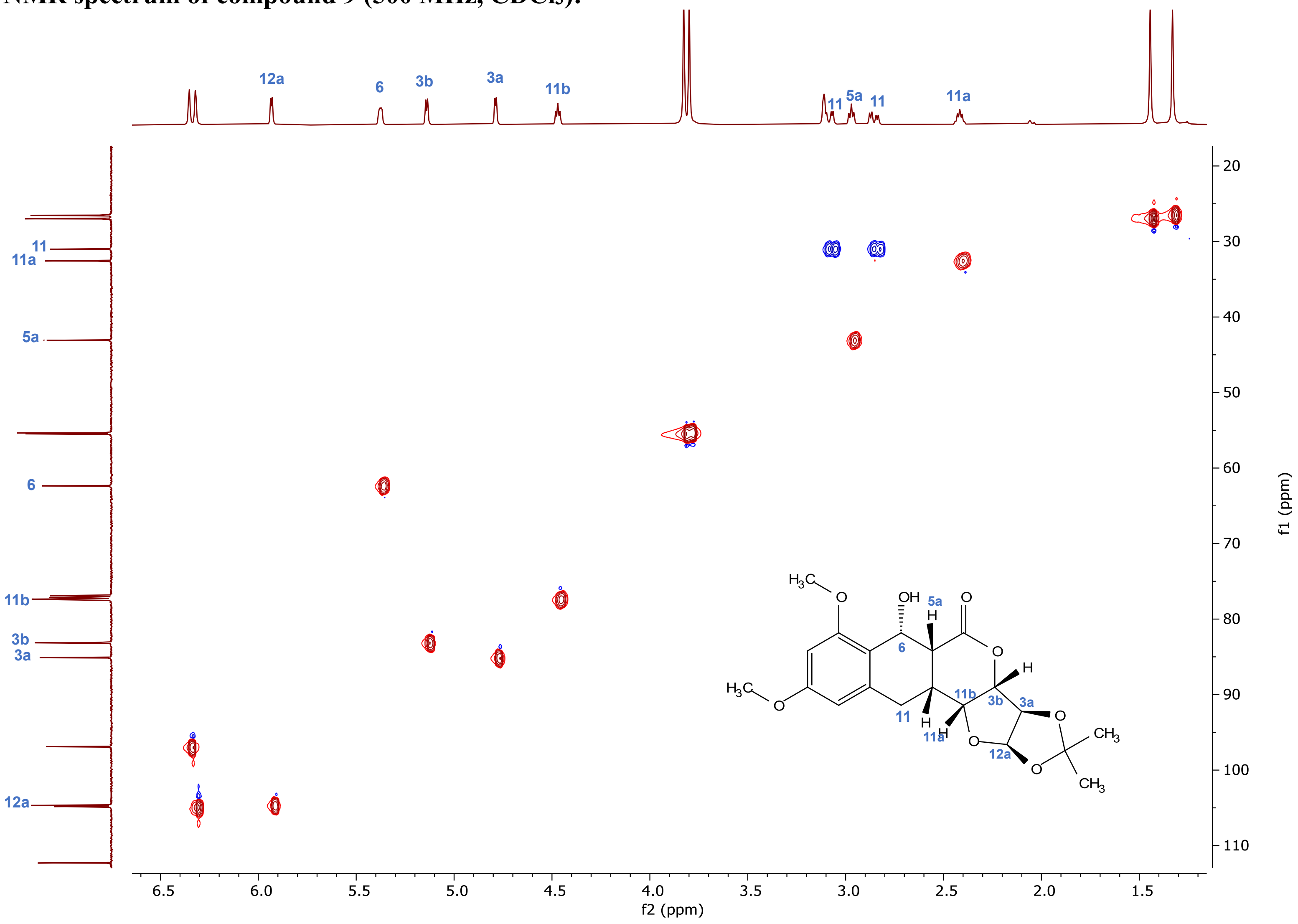

NOESY NMR spectrum of compound 9 (500 MHz, CDCl₃):

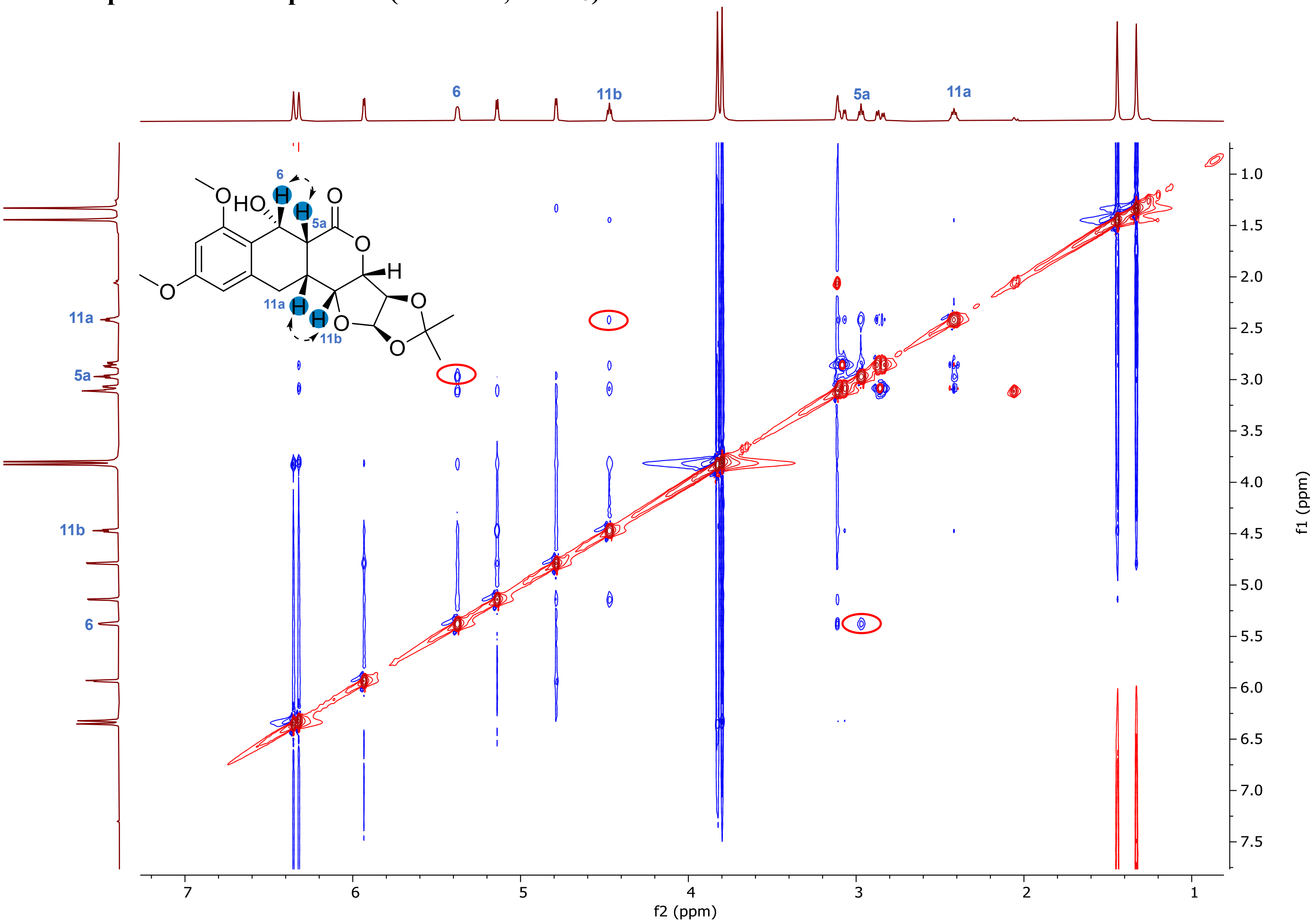

^{1}H NMR spectrum of compound 11 (500 MHz, $CDCl_3$):

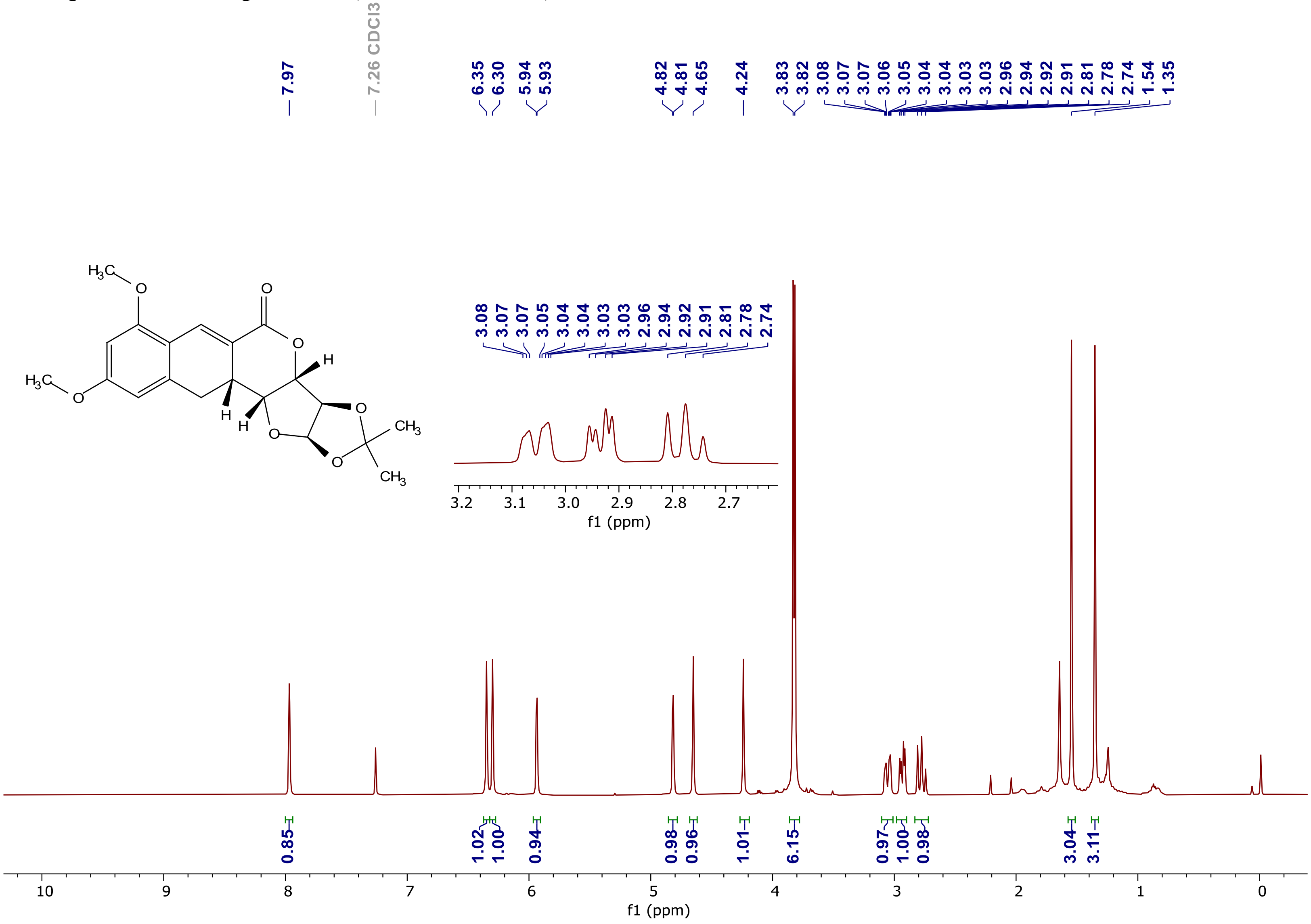

$^{13}C\{^{1}H\}$ NMR spectrum of compound 11 (125 MHz, $CDCl_3$):

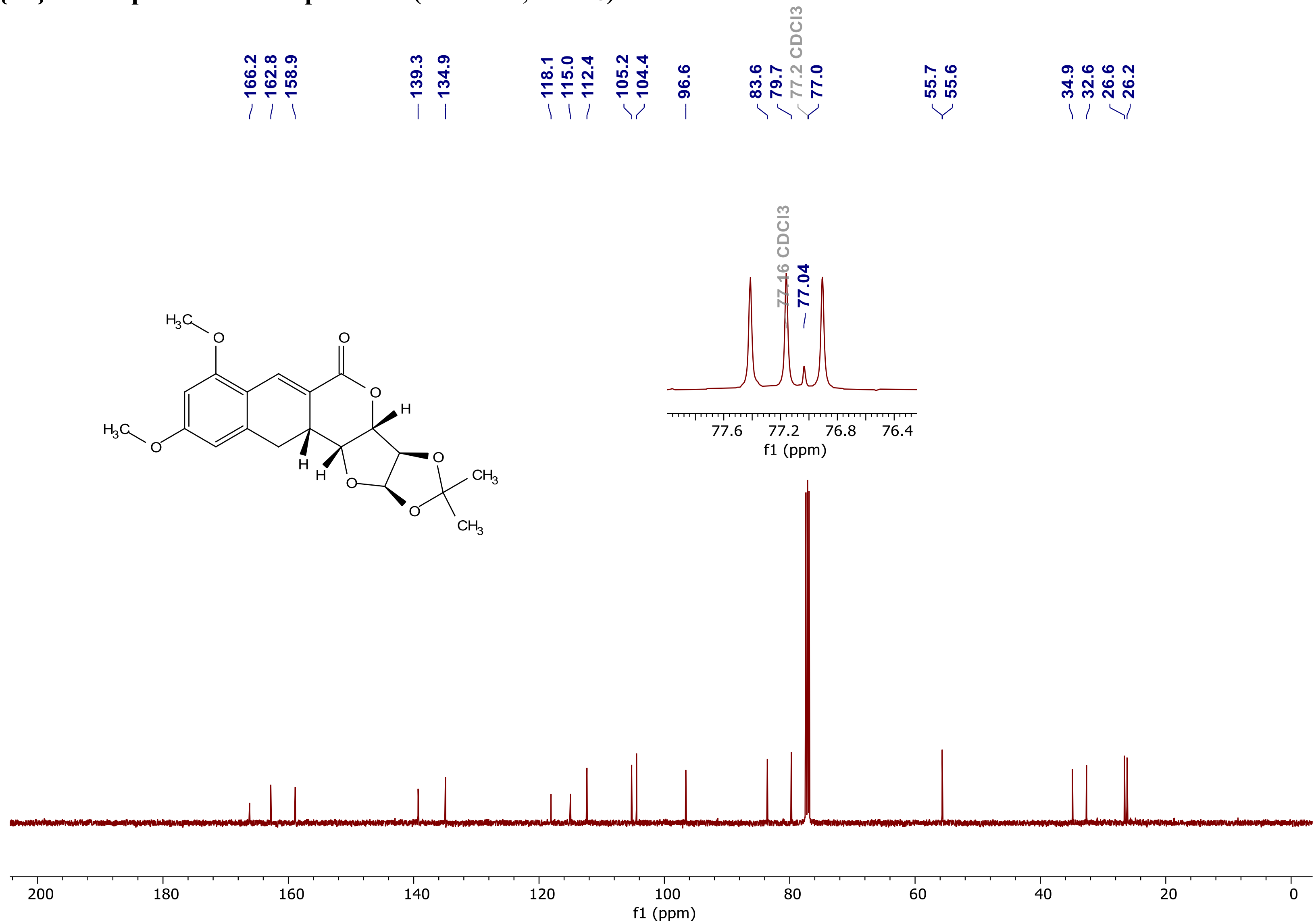

^{1}H NMR spectrum of compound 10 (500 MHz, CDCl3):

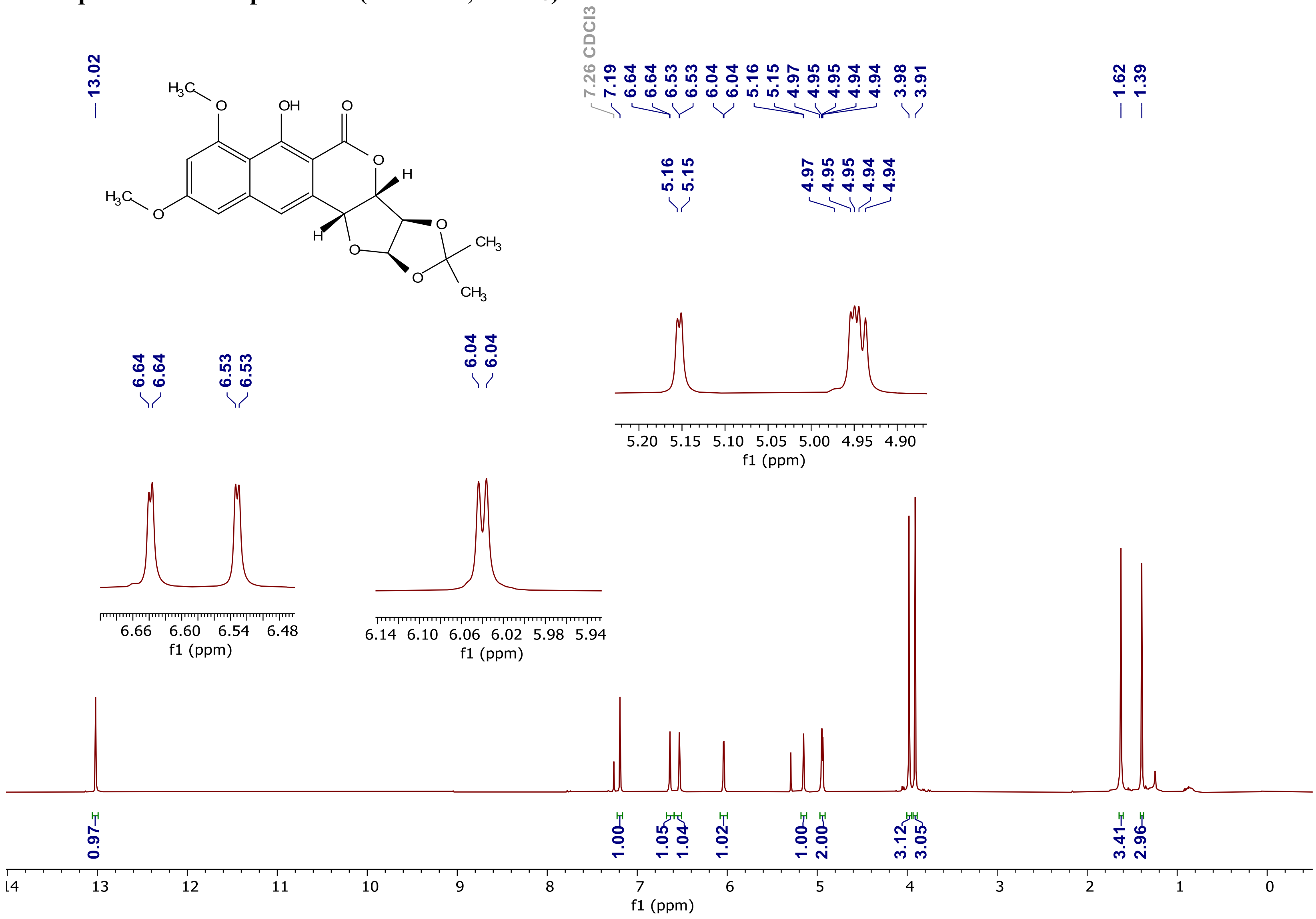

$^{13}C\{^{1}H\}$ NMR spectrum of compound 10 (125 MHz, $CDCl_3$):

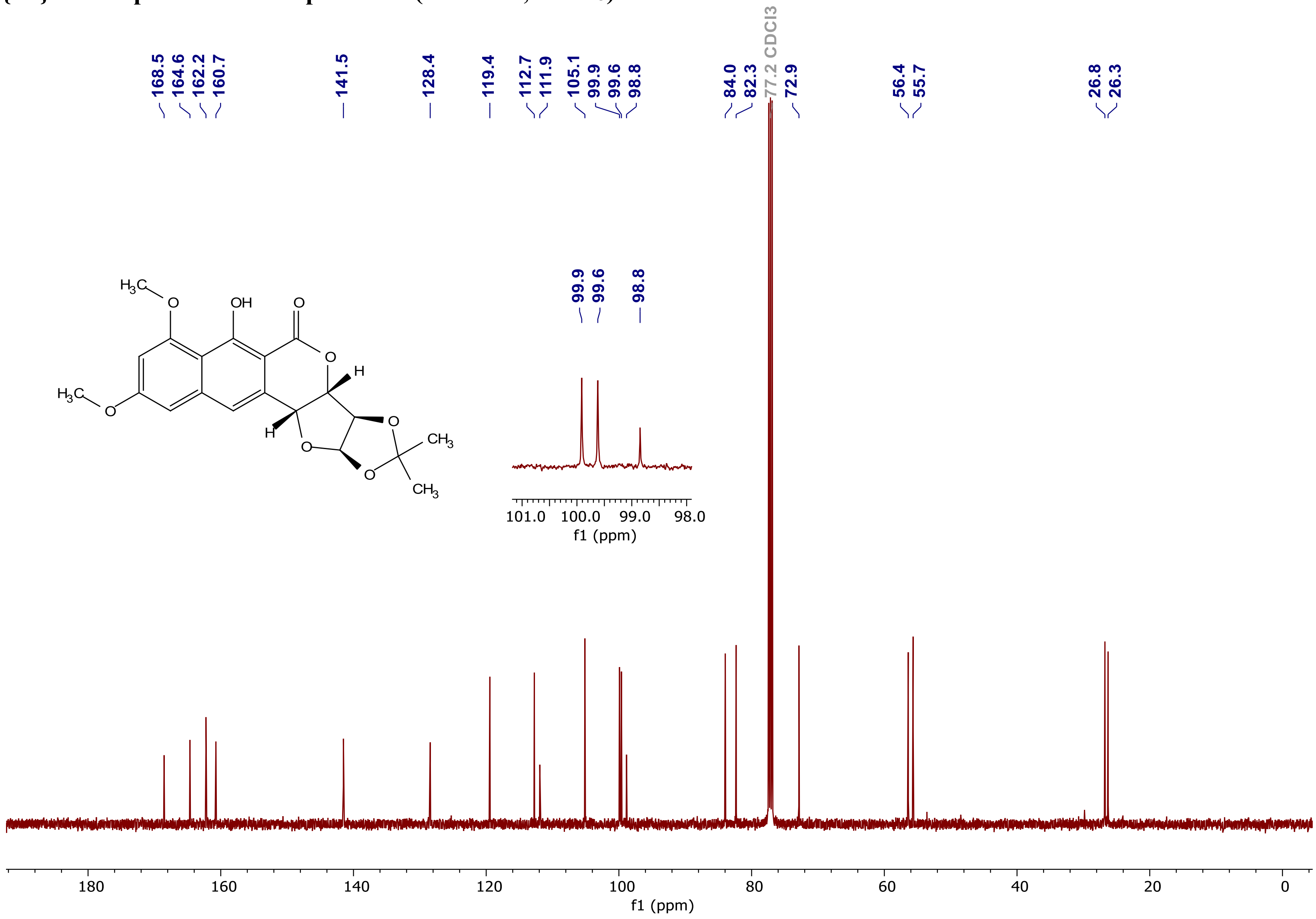

^{1}H NMR spectrum of compound 12 (500 MHz):

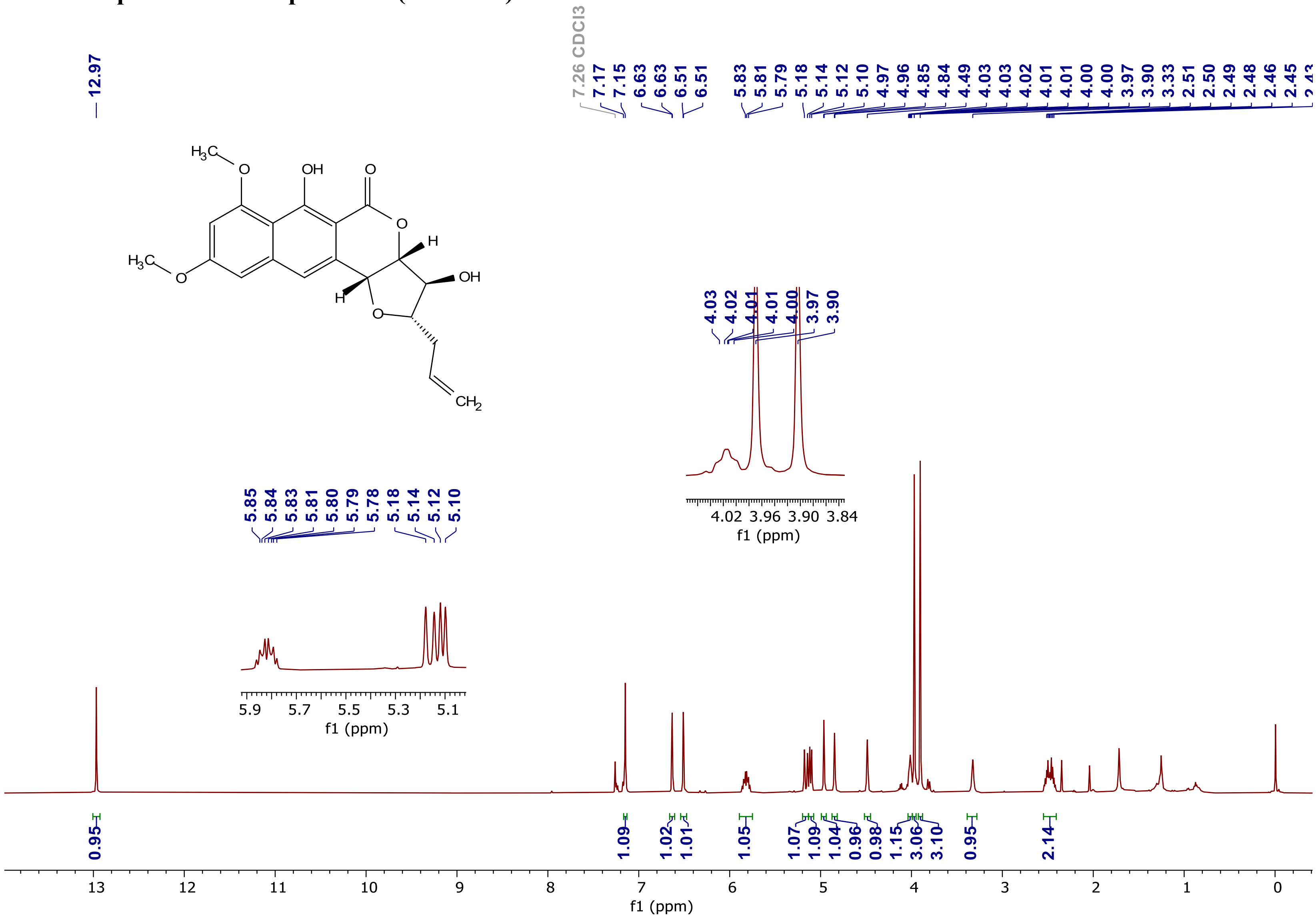

$^{13}C\{^{1}H\}$ NMR spectrum of compound 12 (125 MHz, $CDCl_3$):

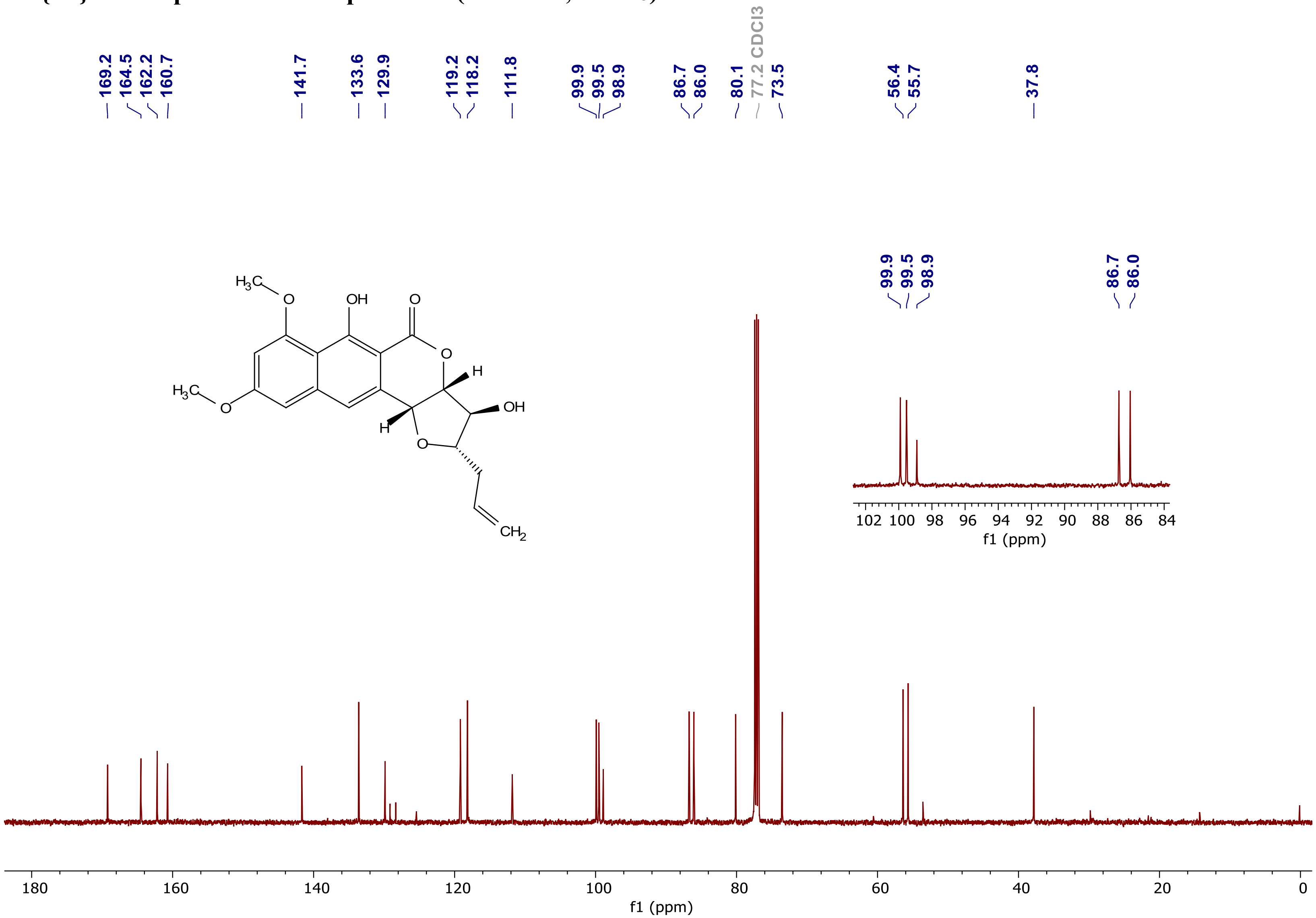

COSY NMR spectrum of compound 12 (500 MHz, $CDCl_3$):

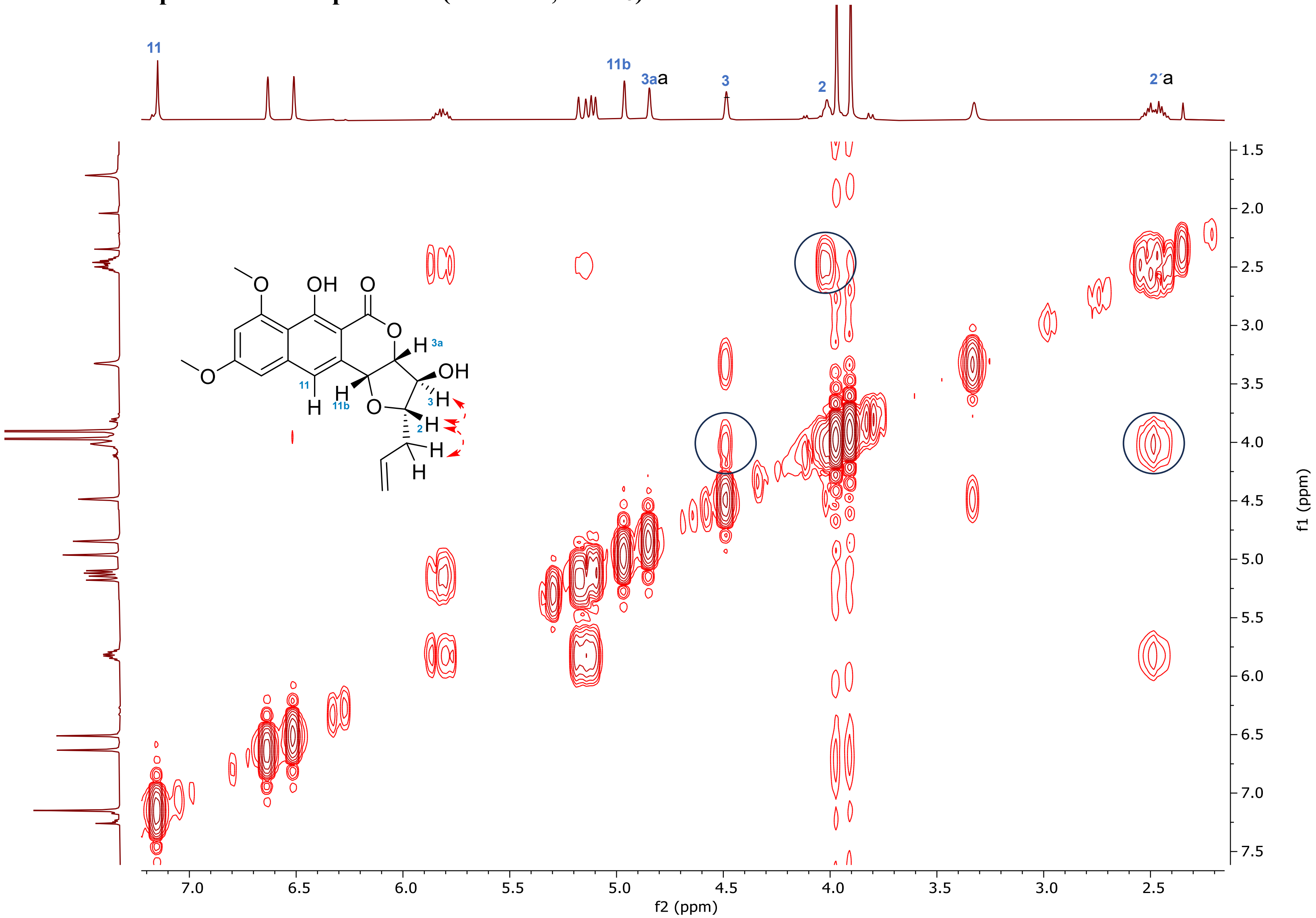

HSQC NMR spectrum of compound 12 (500 MHz, CDCl₃):

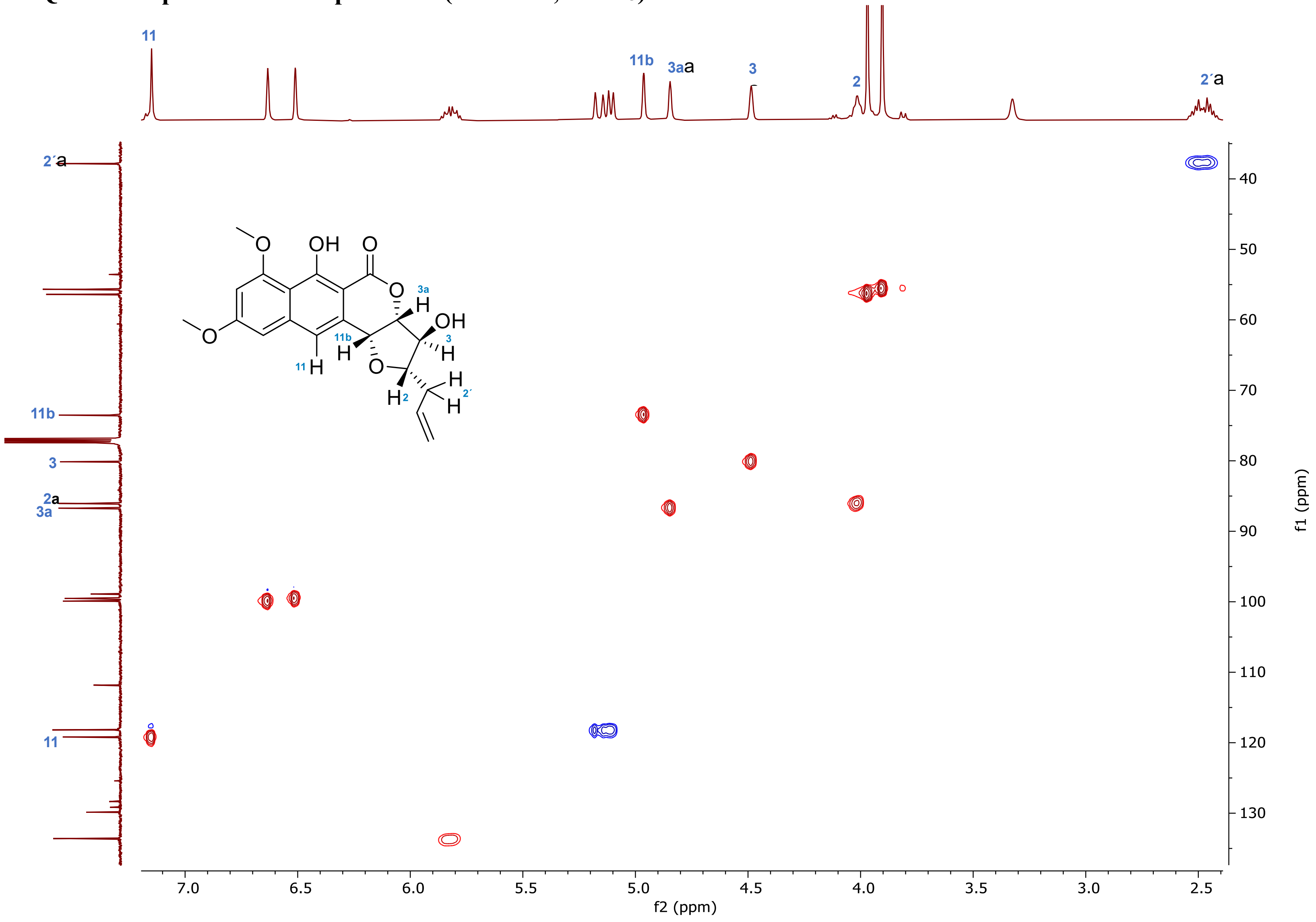

HMBC NMR spectrum of compound 12 (500 MHz, $CDCl_3$):

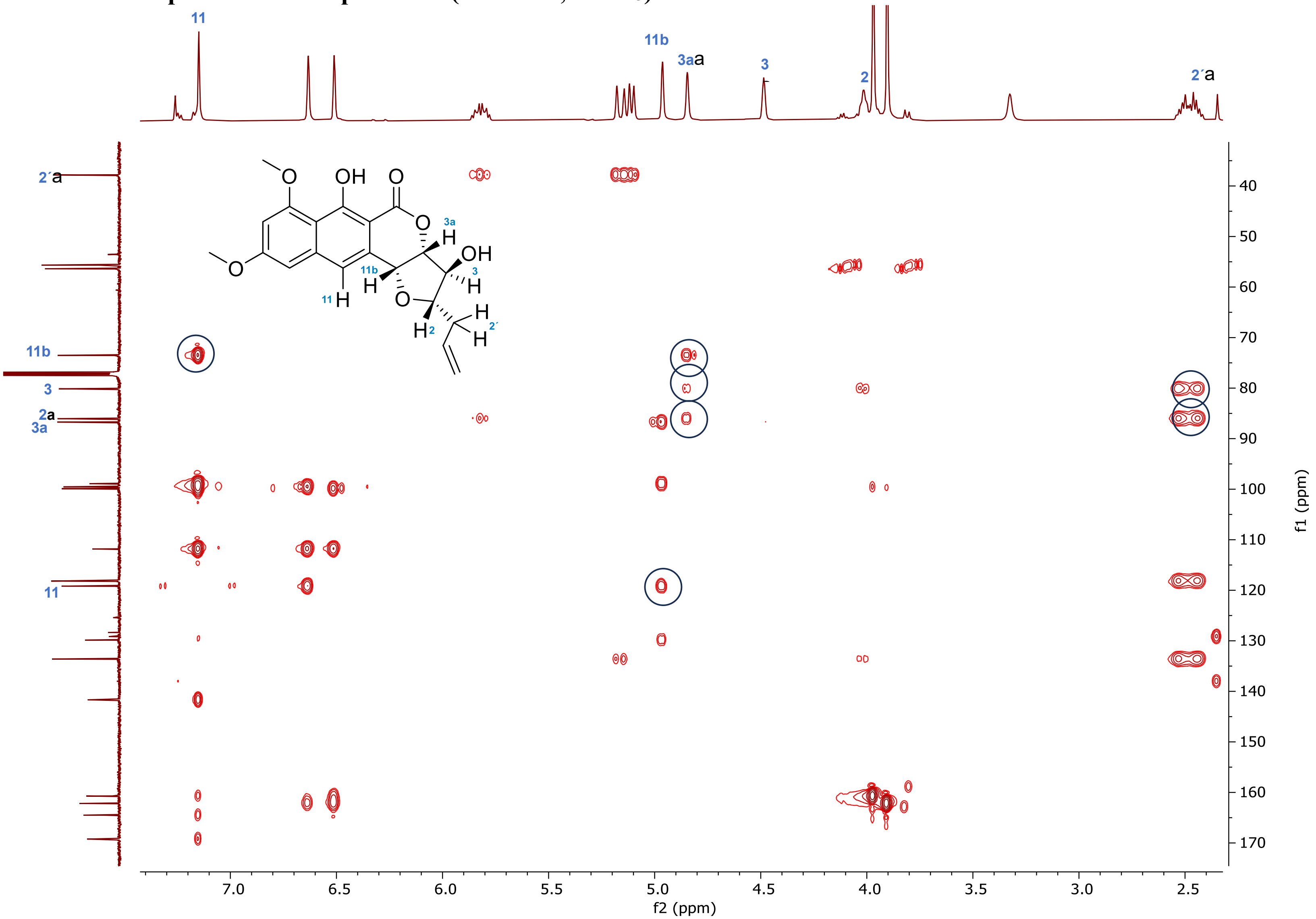

NOESY NMR spectrum of compound 12 (500 MHz, $CDCl_3$):

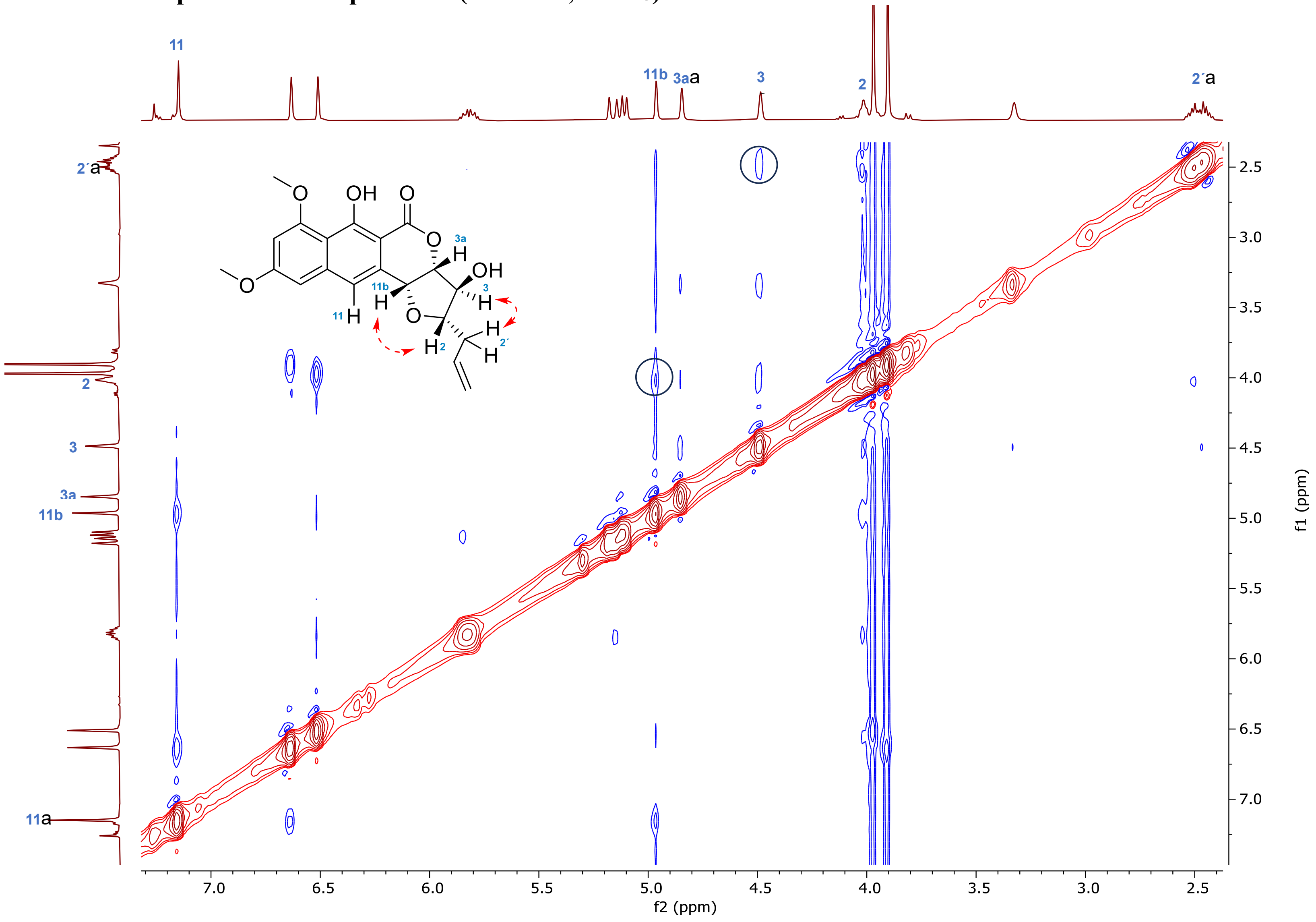

^{1}H NMR spectrum of compound *epi*-13 (500 MHz, CDCl₃):

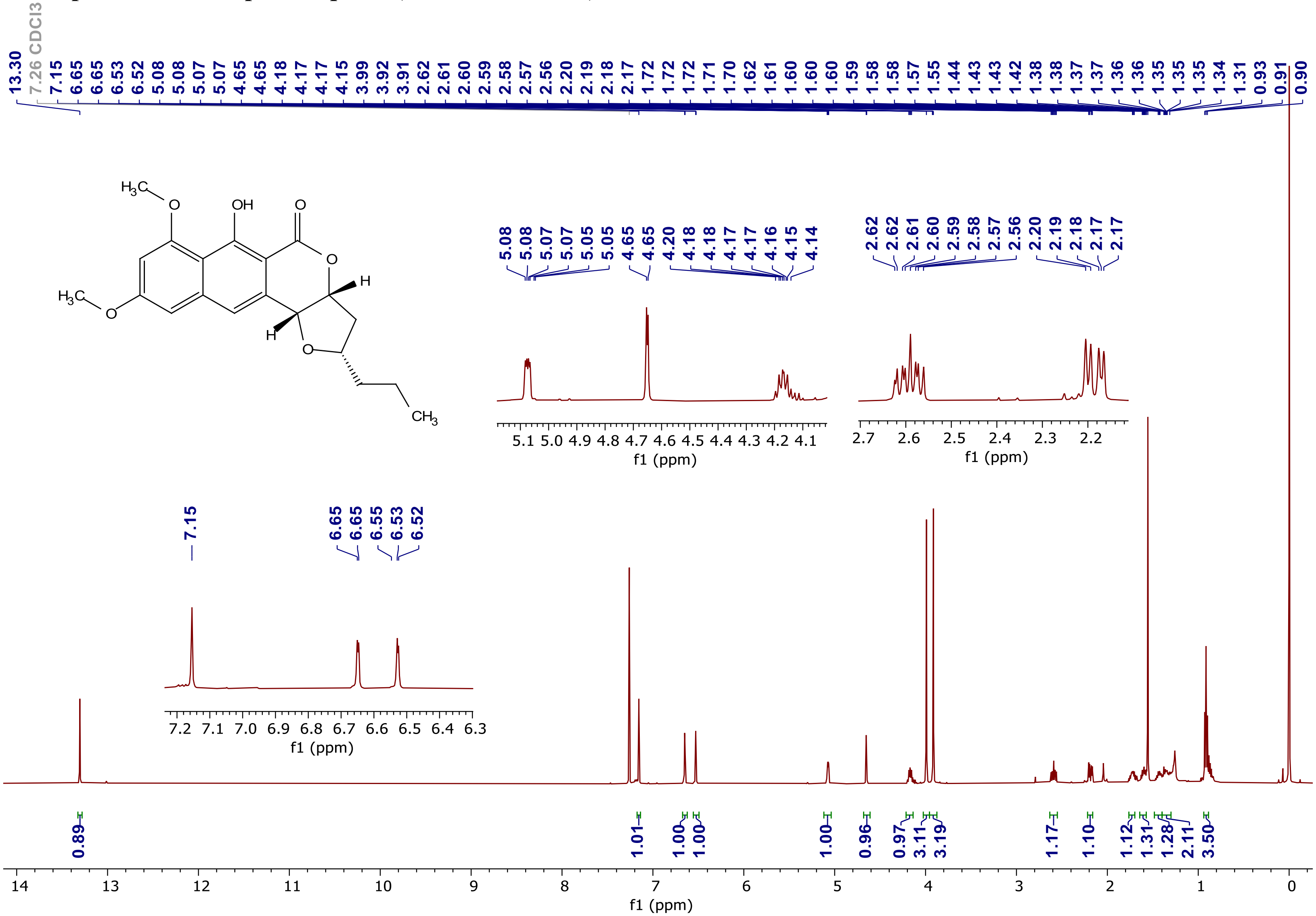

$^{13}C\{^{1}H\}$ NMR spectrum of compound *epi*-13 (125 MHz, CDCl$_3$):

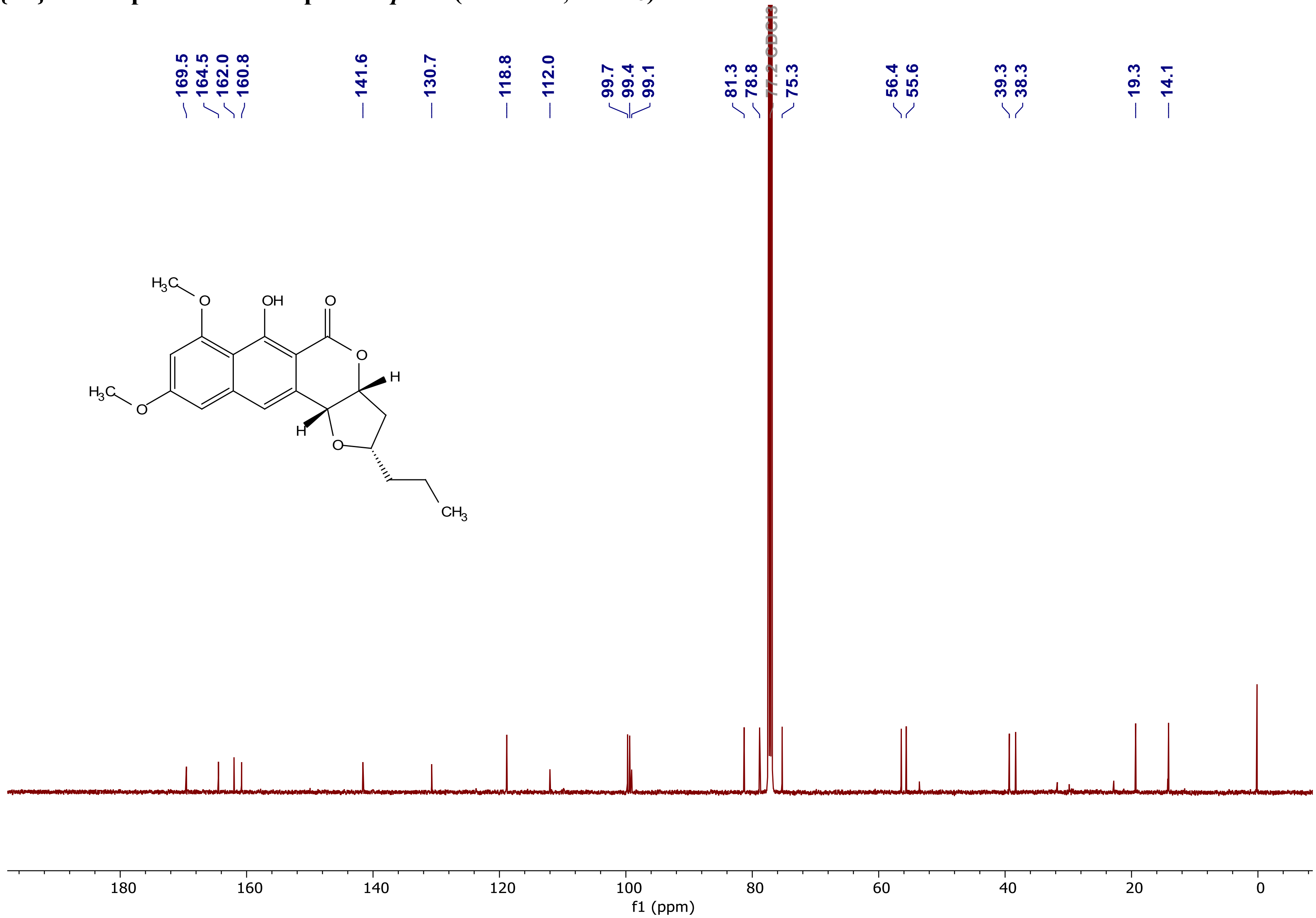

^{1}H NMR spectrum of compound 16 (500 MHz, CDCl$_3$):

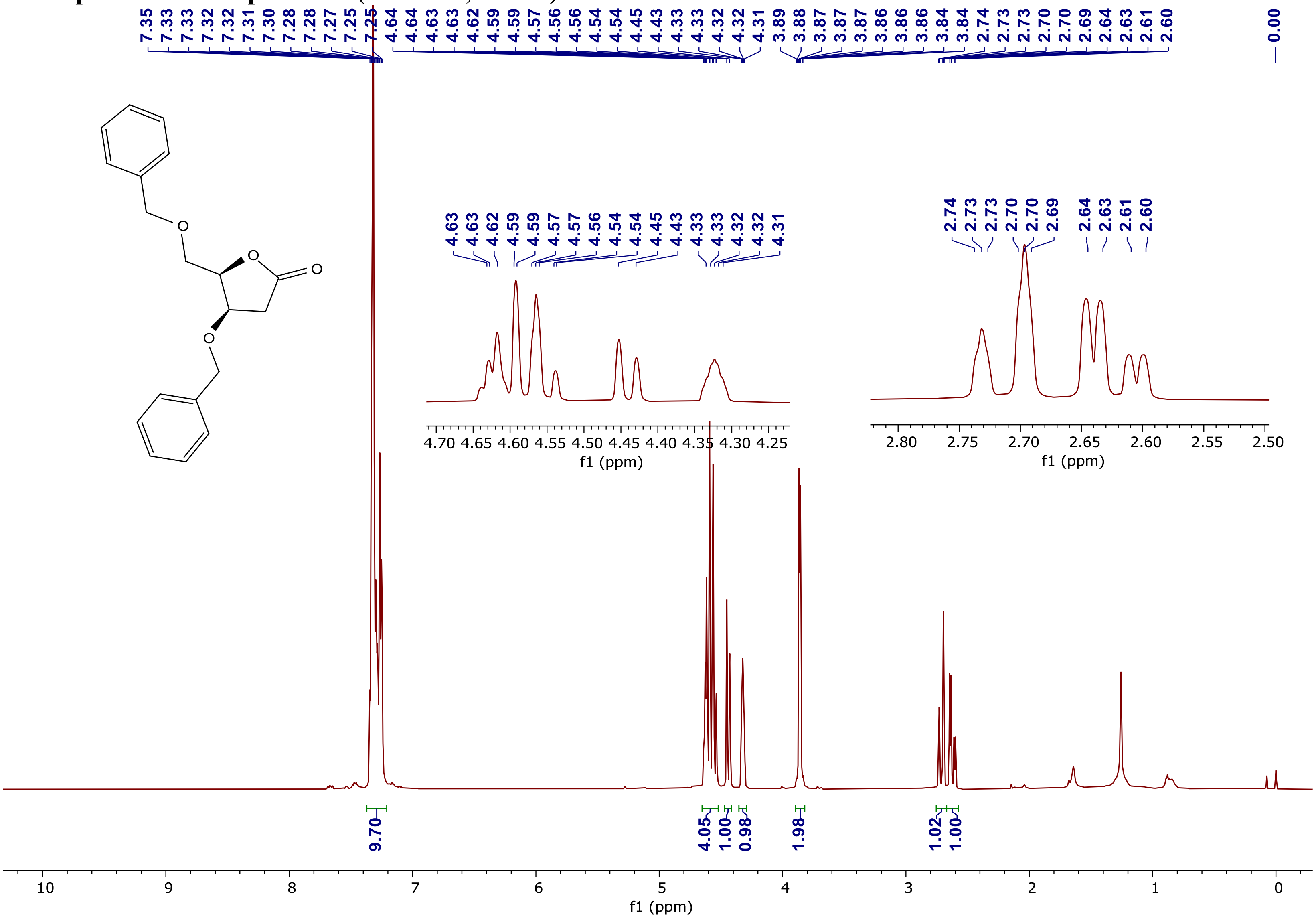

$^{13}C\{^1H\}$ NMR spectrum of compound 16 (125 MHz, $CDCl_3$):

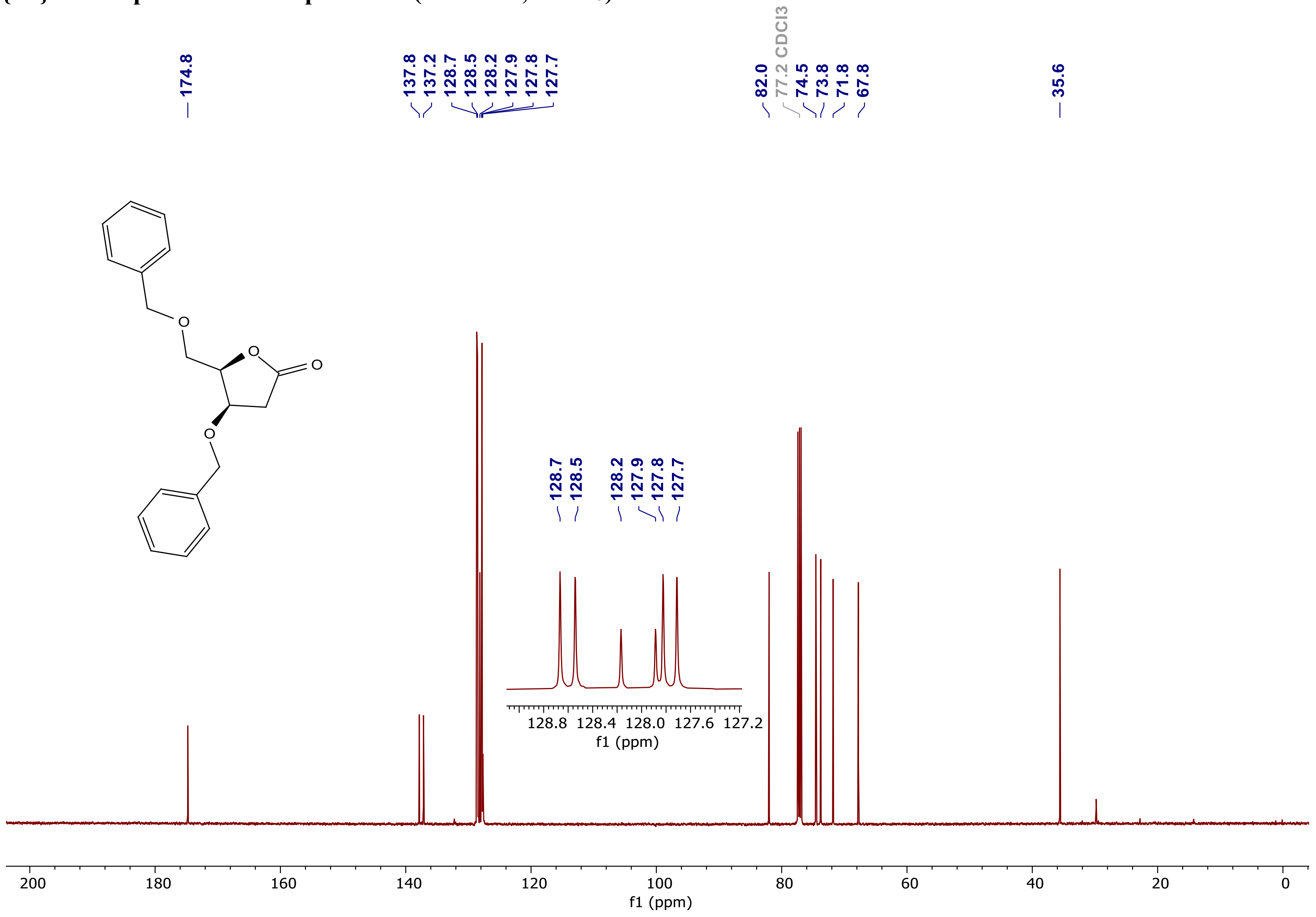

^{1}H NMR spectrum of compound 18 (500 MHz, CDCl3):

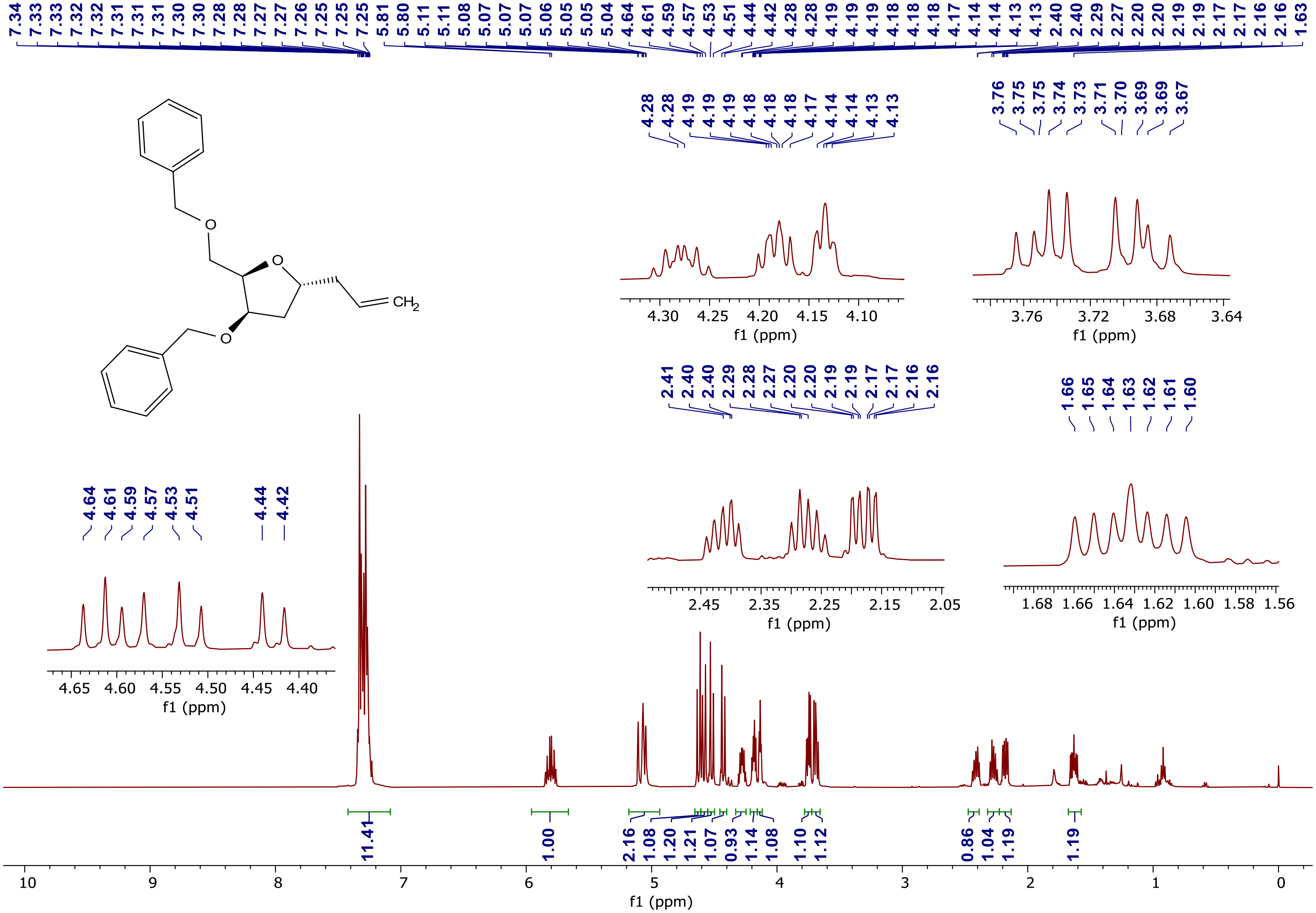

$^{13}C\{^1H\}$ NMR spectrum of compound 18 (125 MHz, $CDCl_3$):

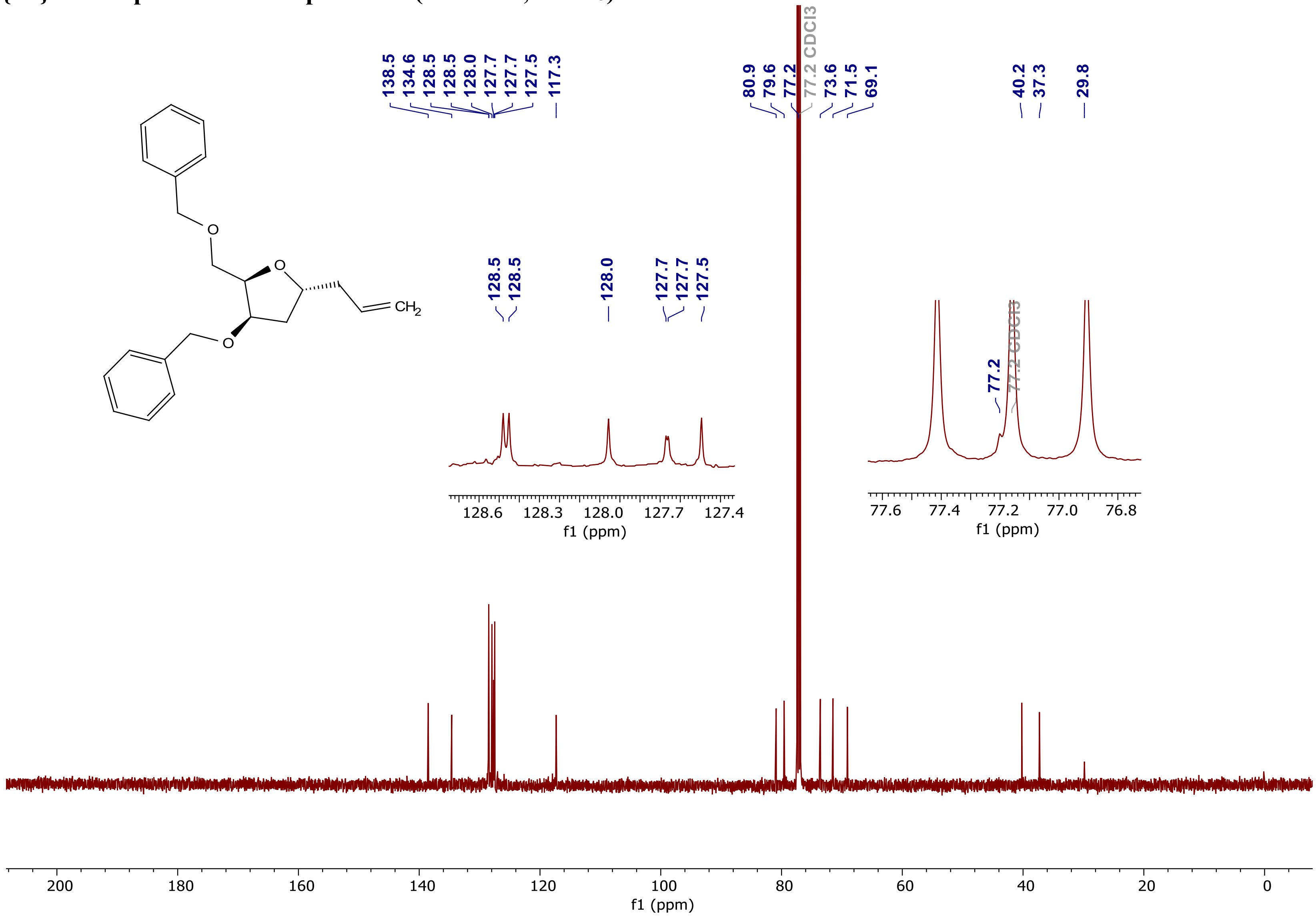

^{1}H NMR spectrum of compound 19 (500 MHz, $CDCl_3$):

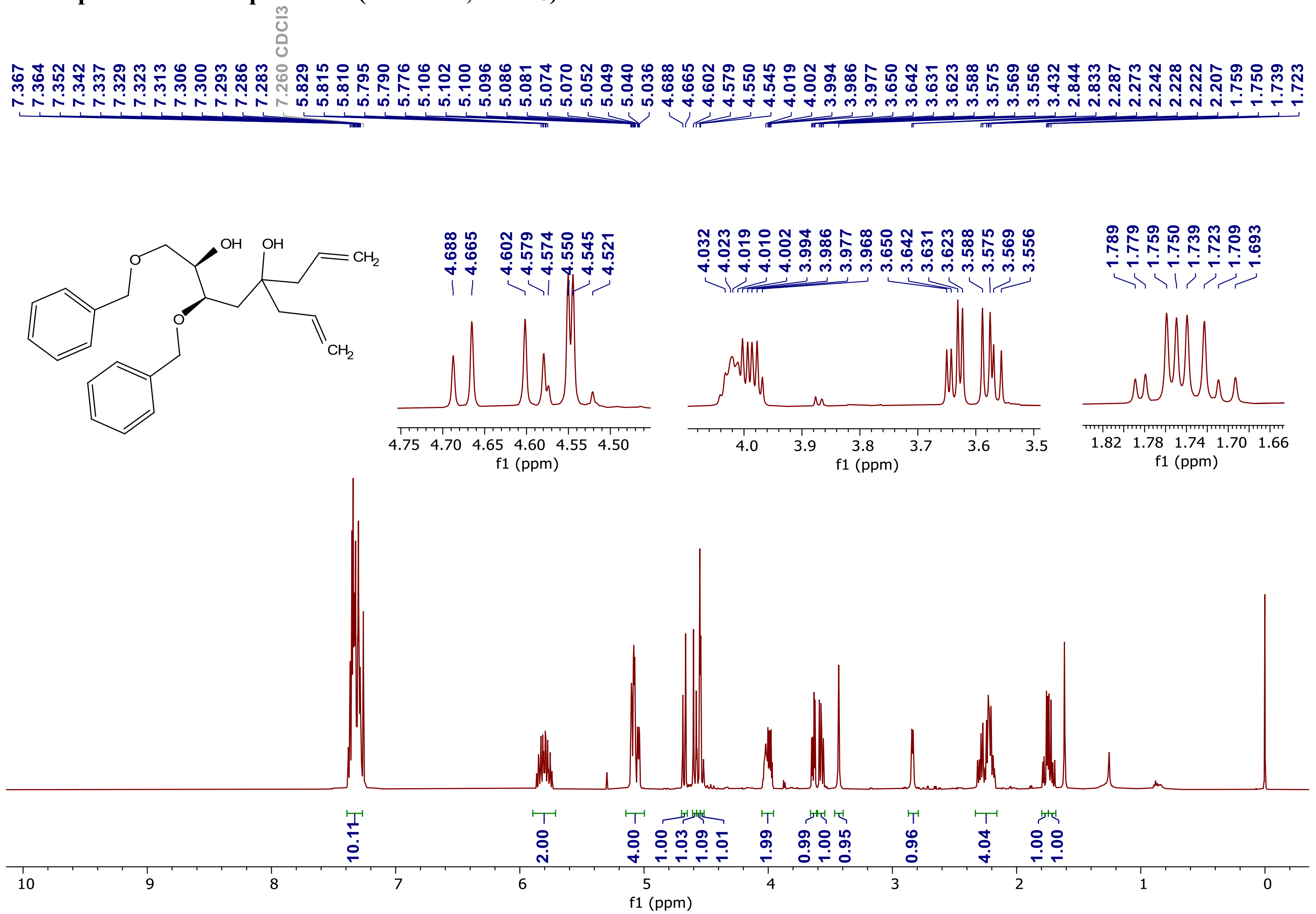

$^{13}C\{^{1}H\}$ NMR spectrum of compound 19 (125 MHz, $CDCl_3$):

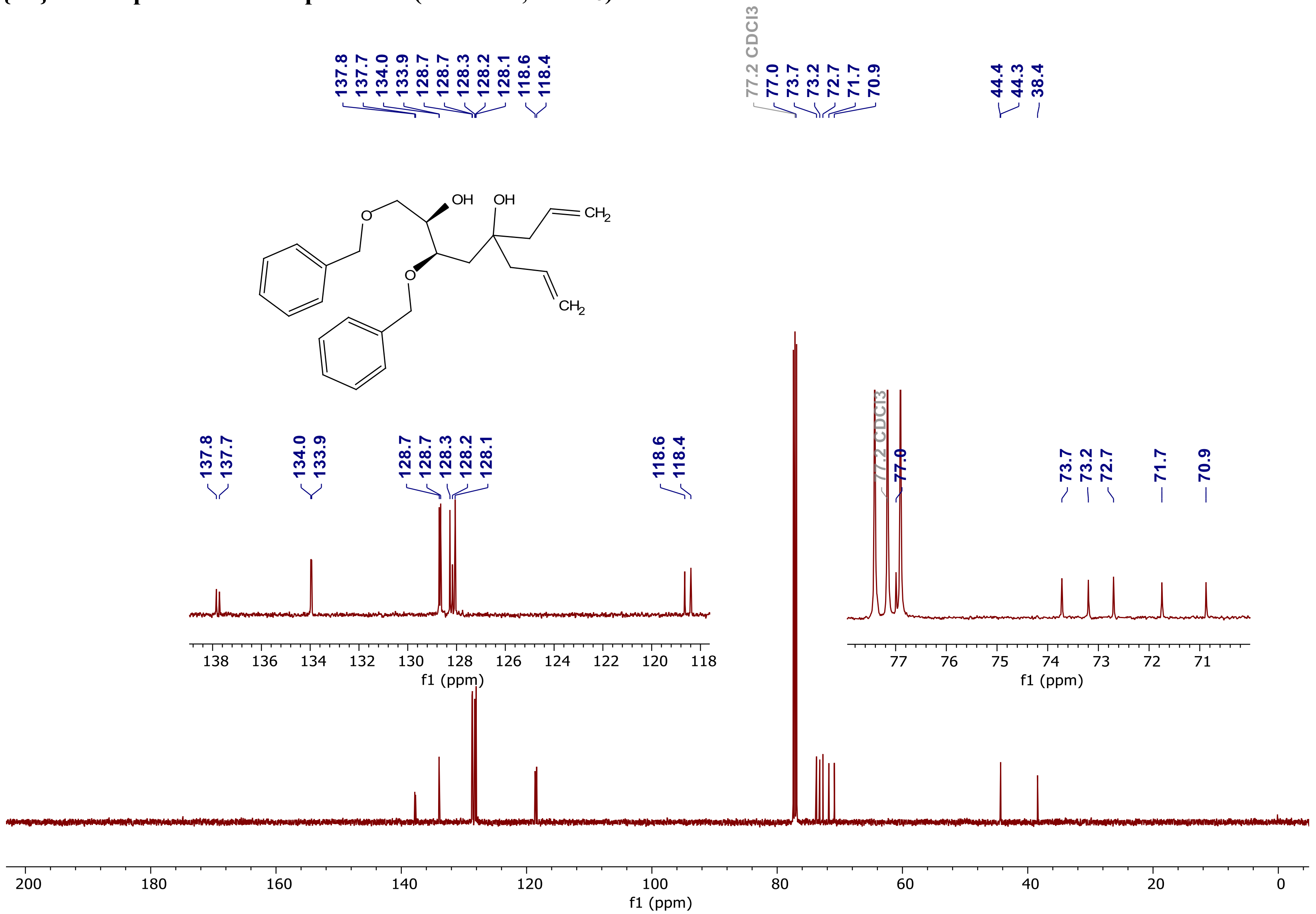

^{1}H NMR spectrum of compound 20 (500 MHz, CDCl$_3$):

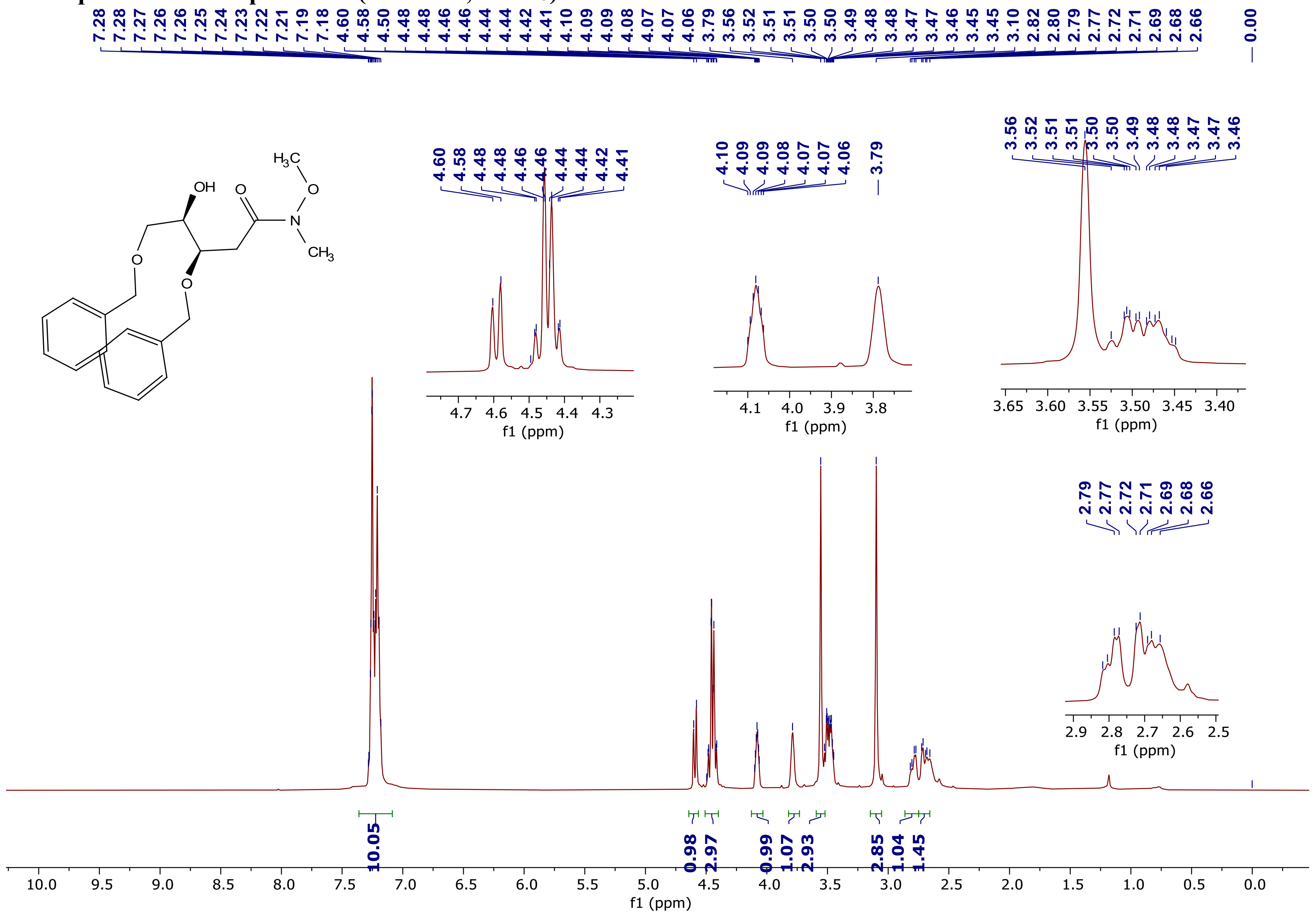

$^{13}C\{^1H\}$ NMR spectrum of compound 20 (125 MHz, $CDCl_3$):

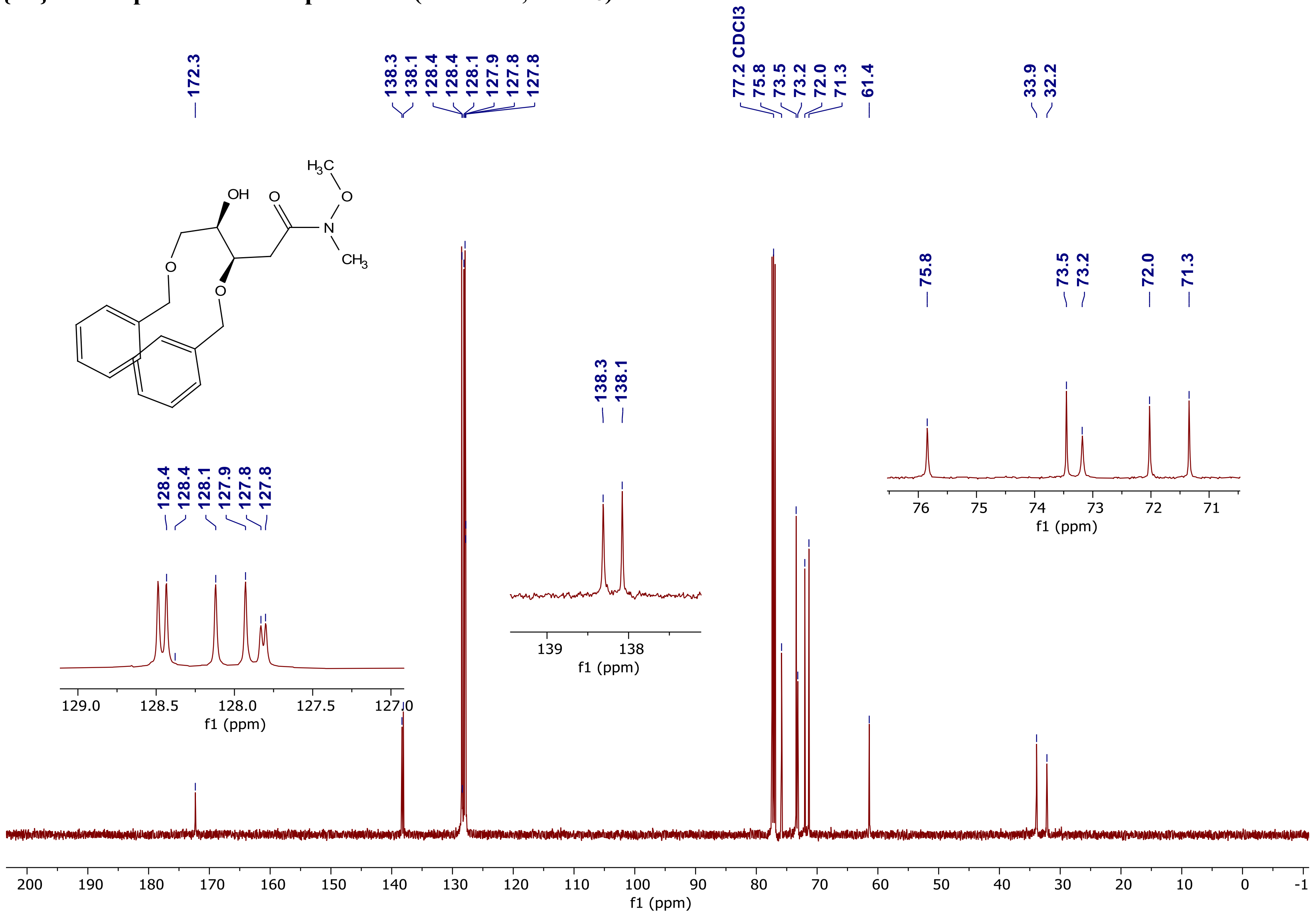

^{1}H NMR spectrum of compound 21 (500 MHz, $CDCl_3$):

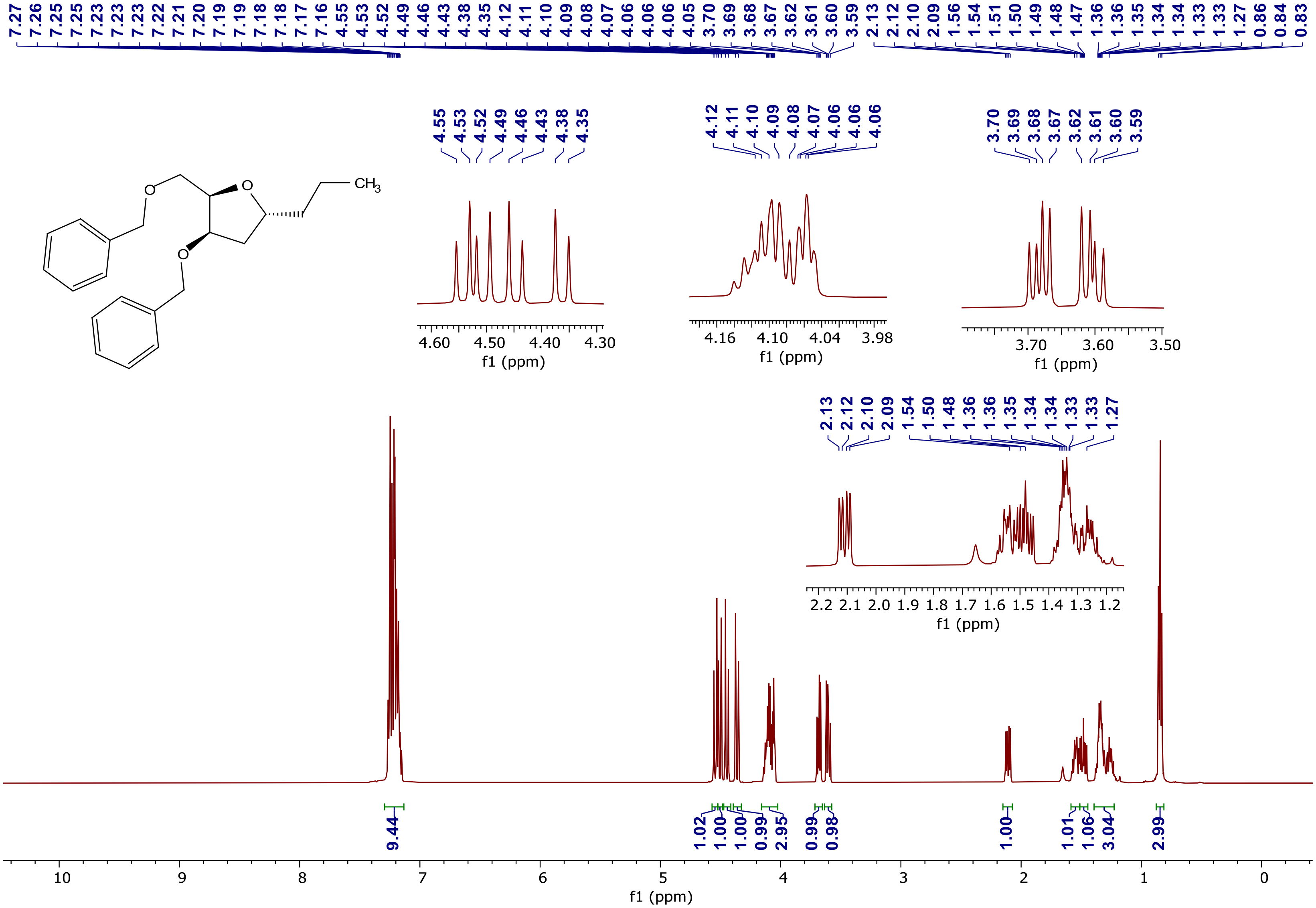

$^{13}C\{^{1}H\}$ NMR spectrum of compound 21 (125 MHz, $CDCl_3$):

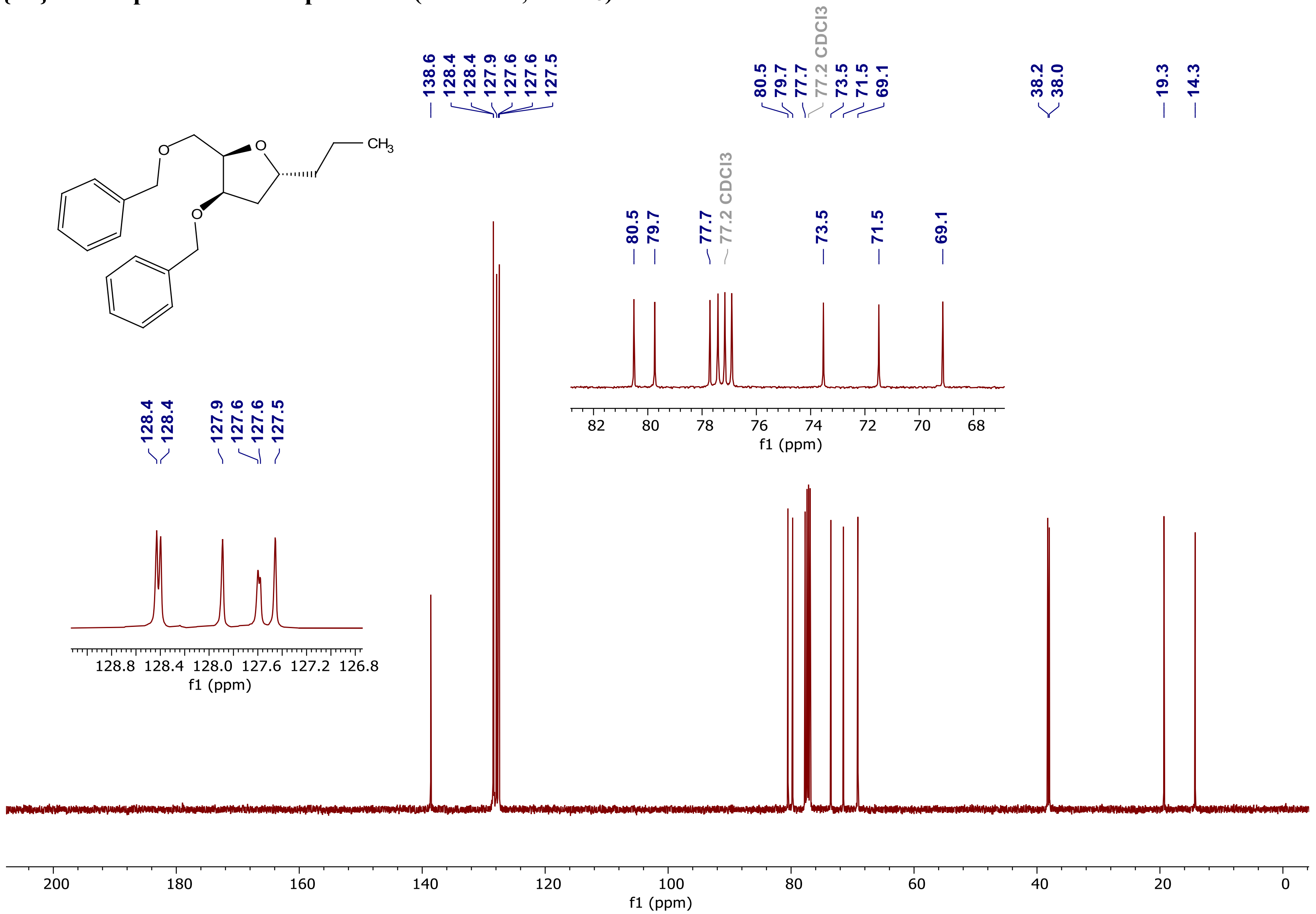

^{1}H NMR spectra of compound 15 (500 MHz, $CDCl_3$):

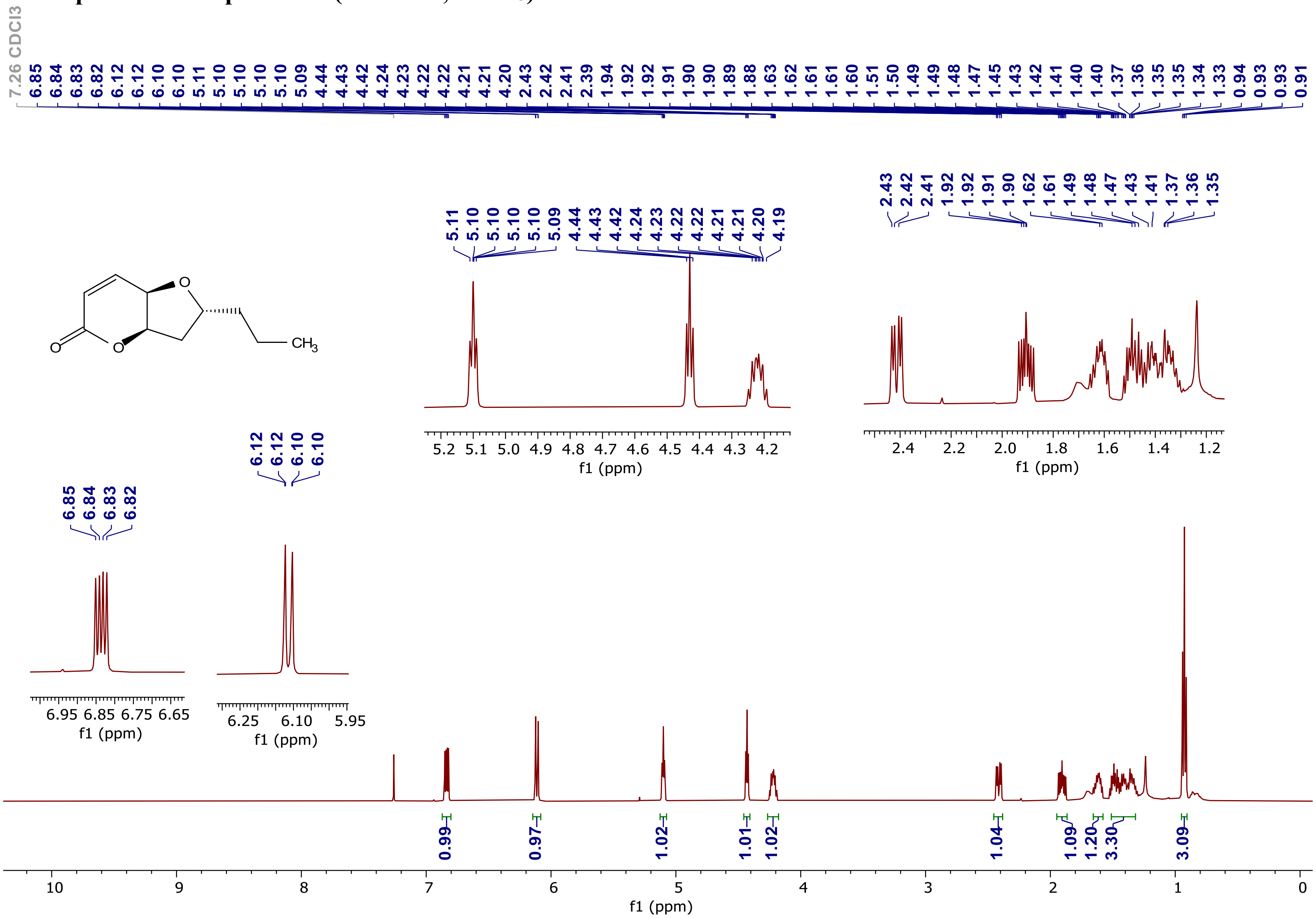

$^{13}C\{^1H\}$ NMR spectrum of compound 15 (125 MHz, $CDCl_3$):

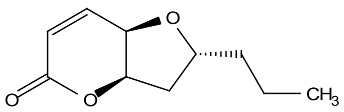

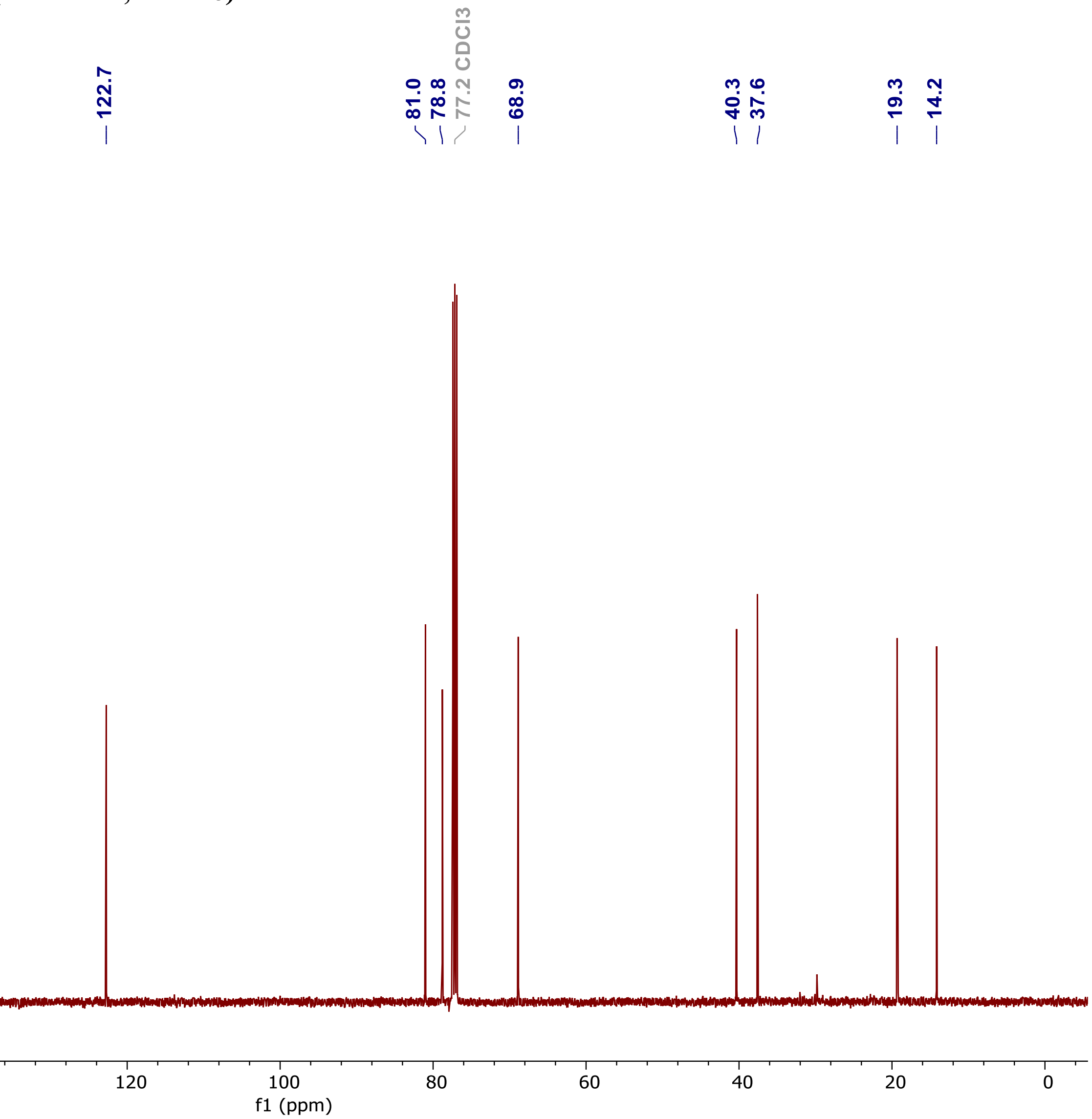

^{1}H NMR spectrum of compound 23 (500 MHz, $CDCl_3$):

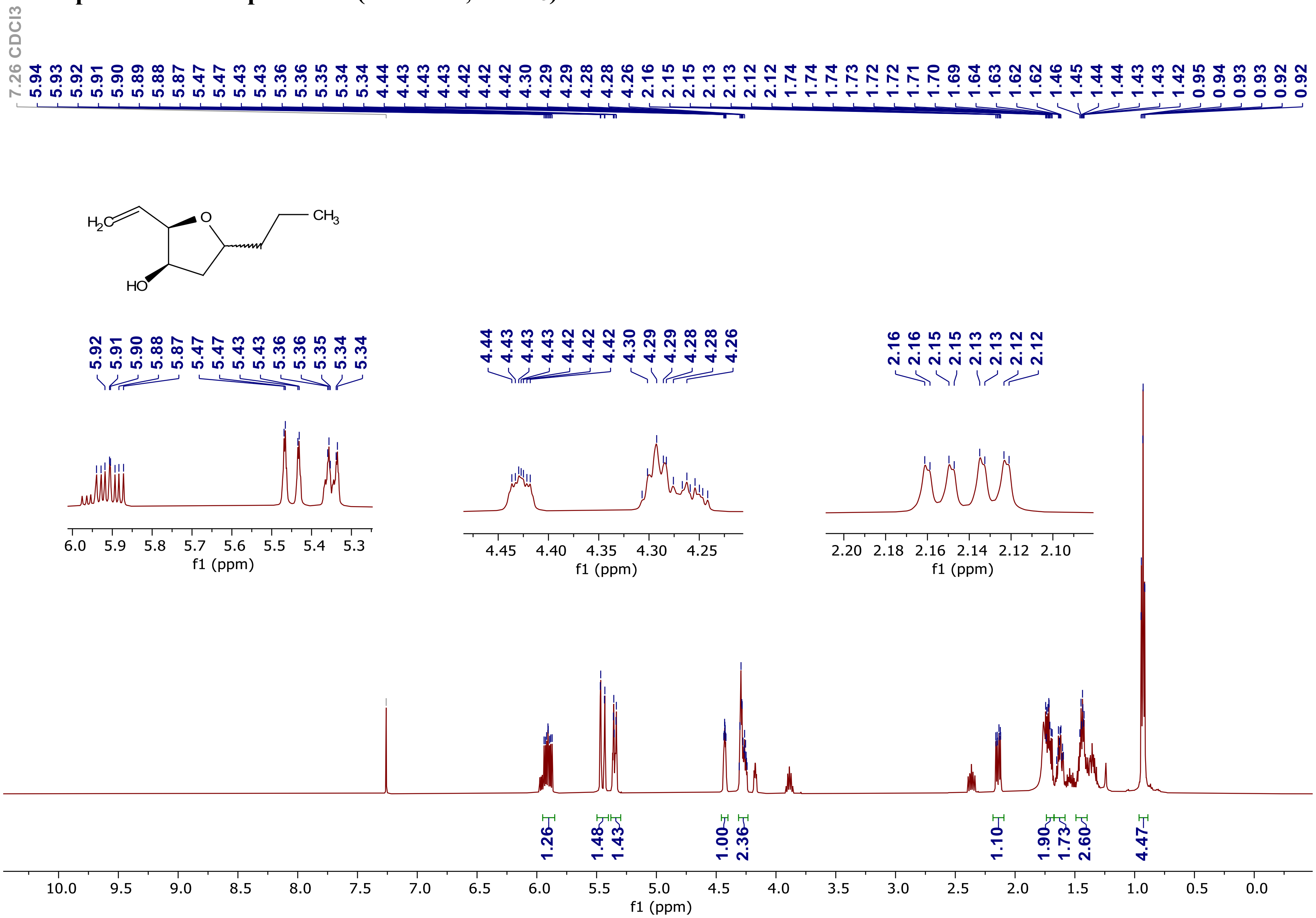

$^{13}C\{^1H\}$ NMR spectrum of compound 23 (125 MHz, $CDCl_3$):

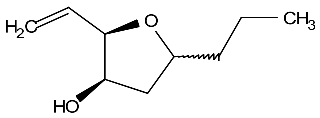

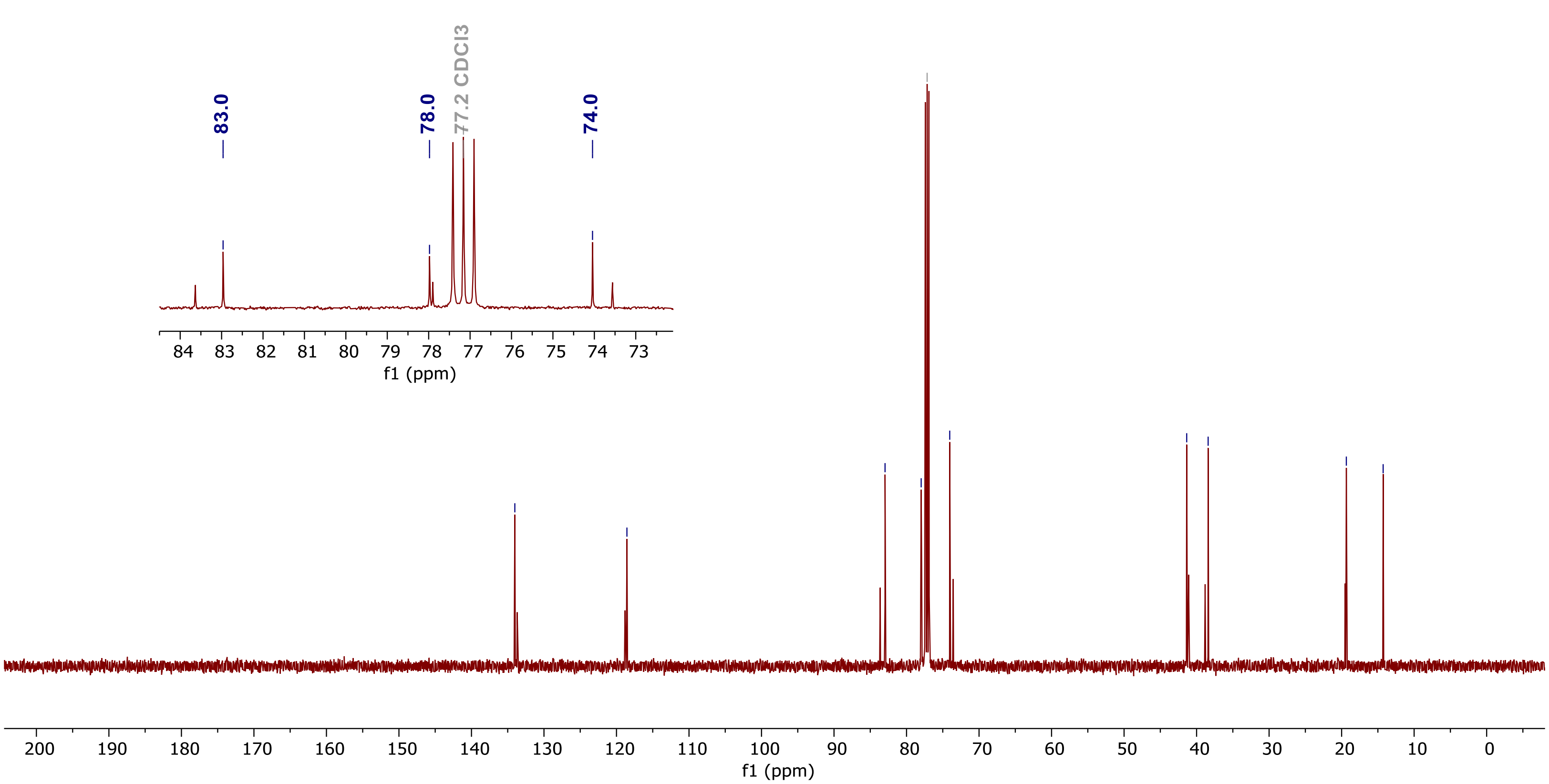

^{1}H NMR spectrum of compound 24 (500 MHz, $CDCl_3$):

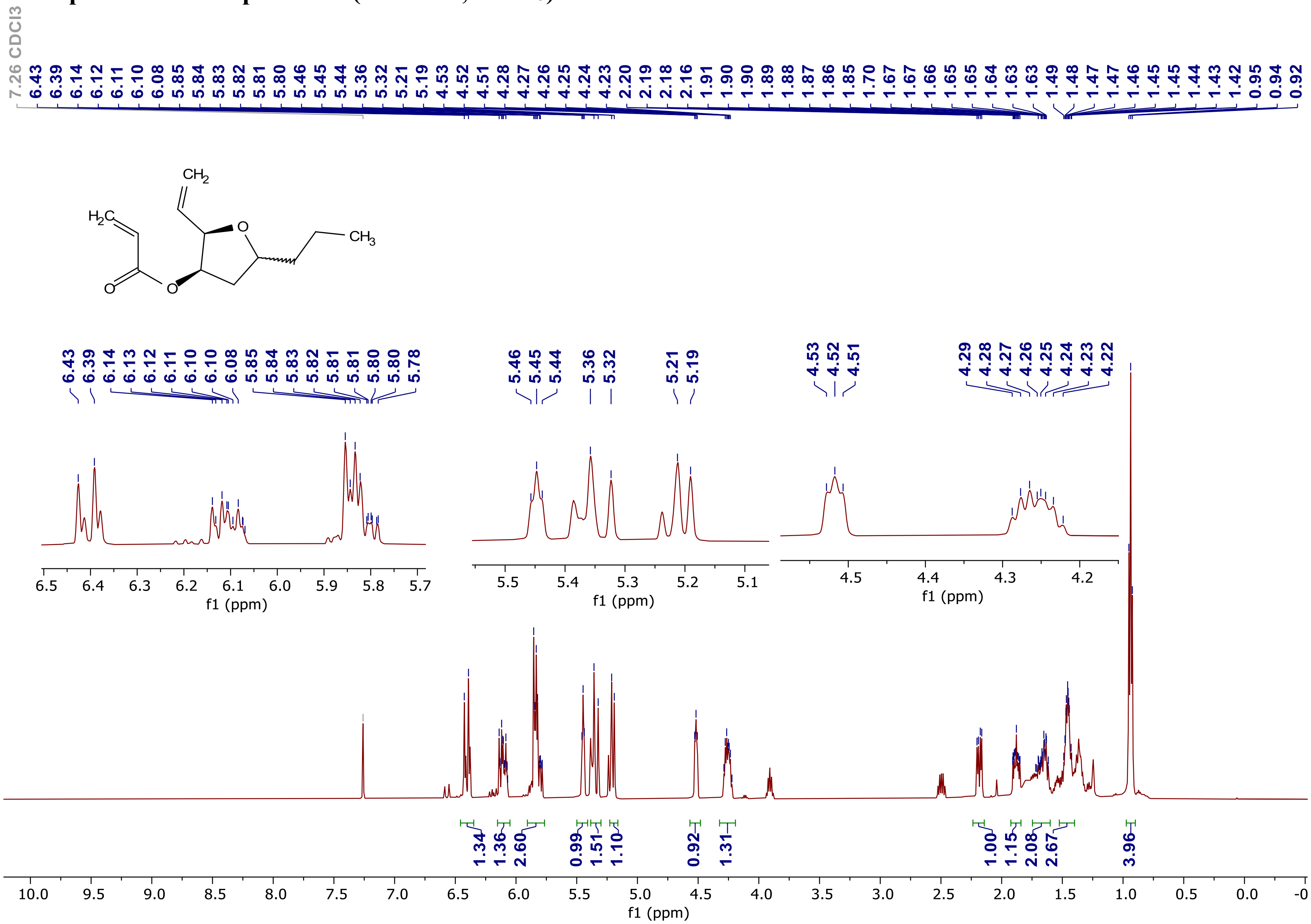

$^{13}C\{^{1}H\}$ NMR spectrum of compound 24 (125 MHz, $CDCl_3$):

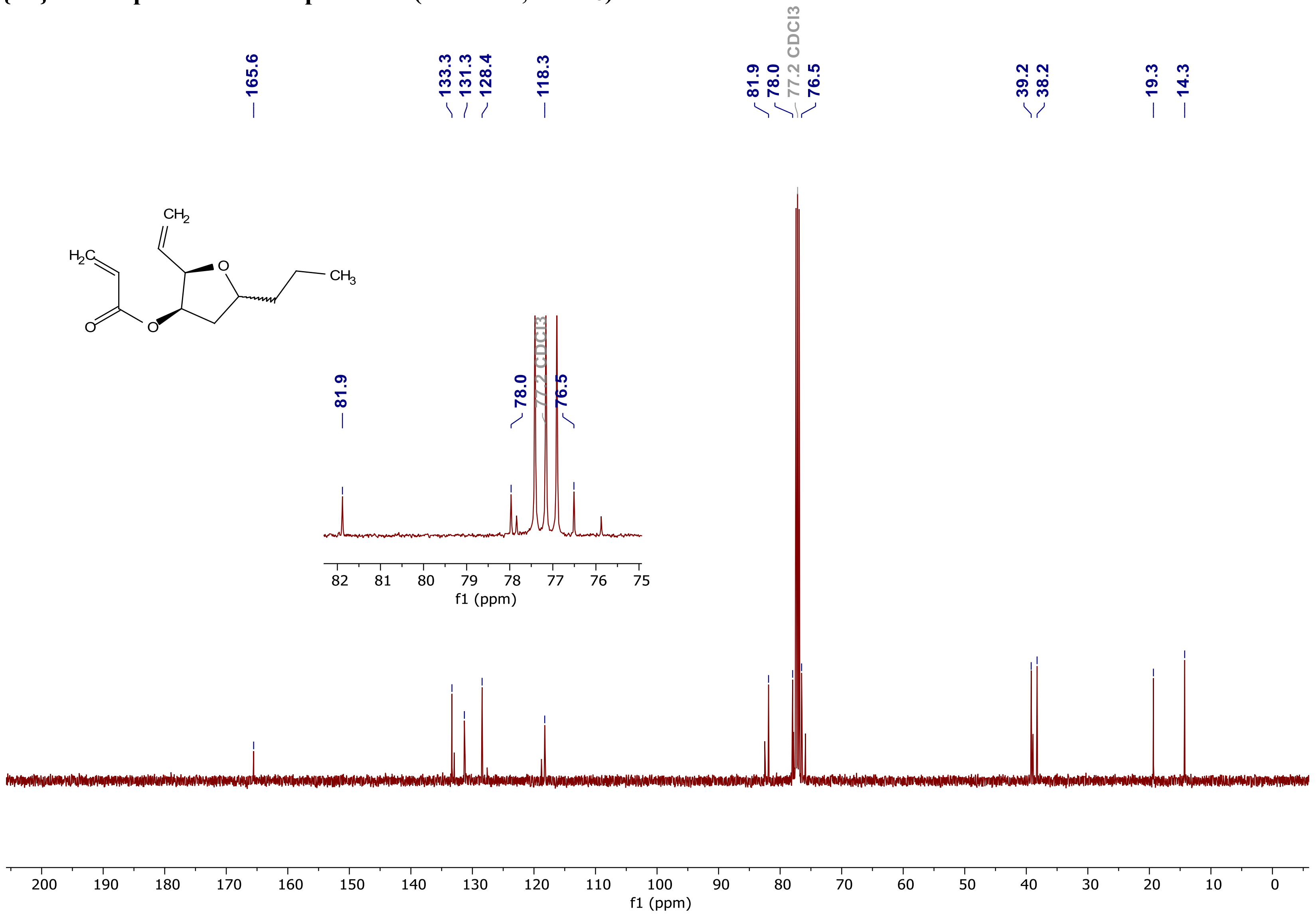

^{1}H NMR spectrum of compound 25 (500 MHz, $CDCl_3$):

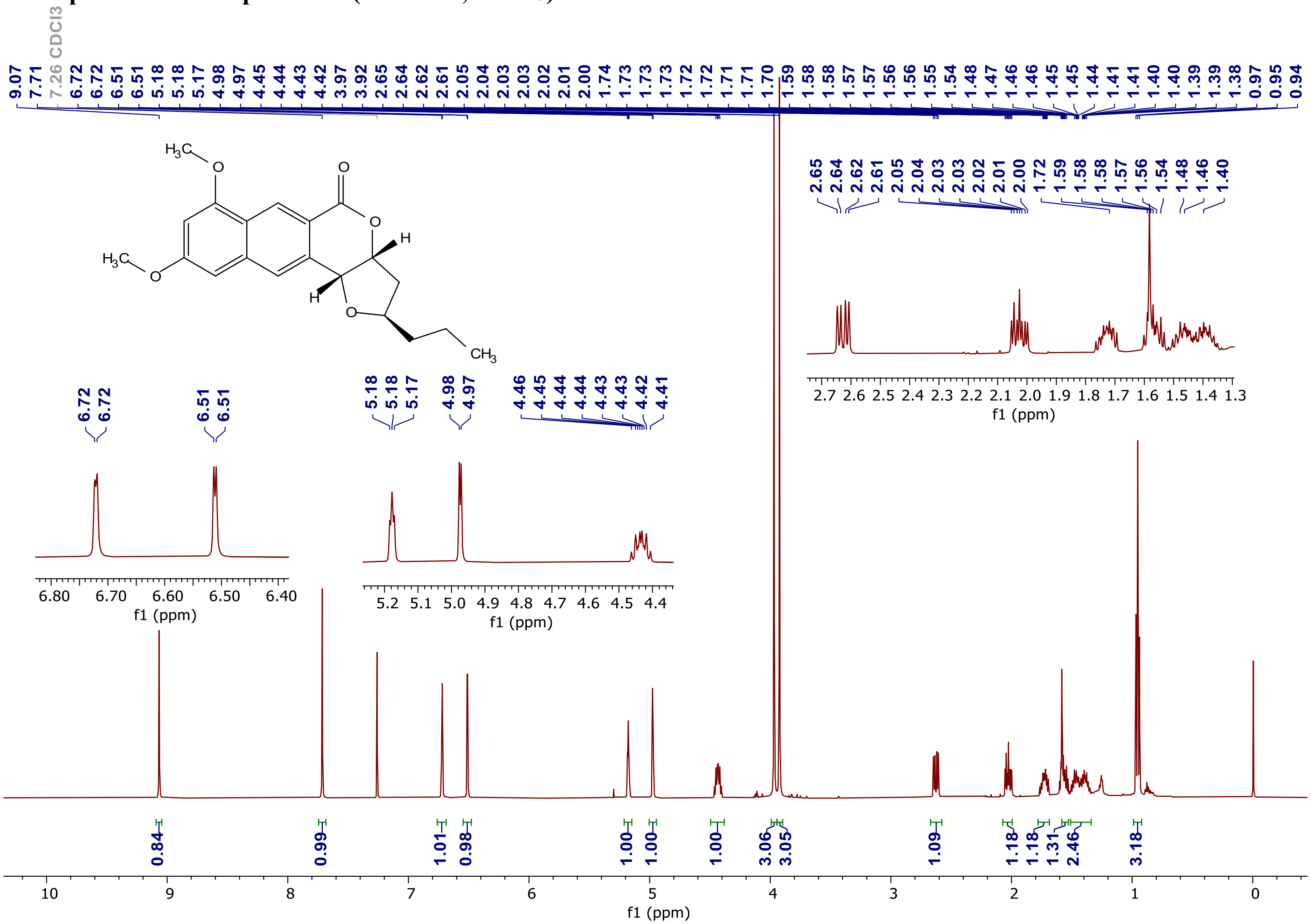

$^{13}C\{^1H\}$ NMR spectrum of 25 (125 MHz, $CDCl_3$):

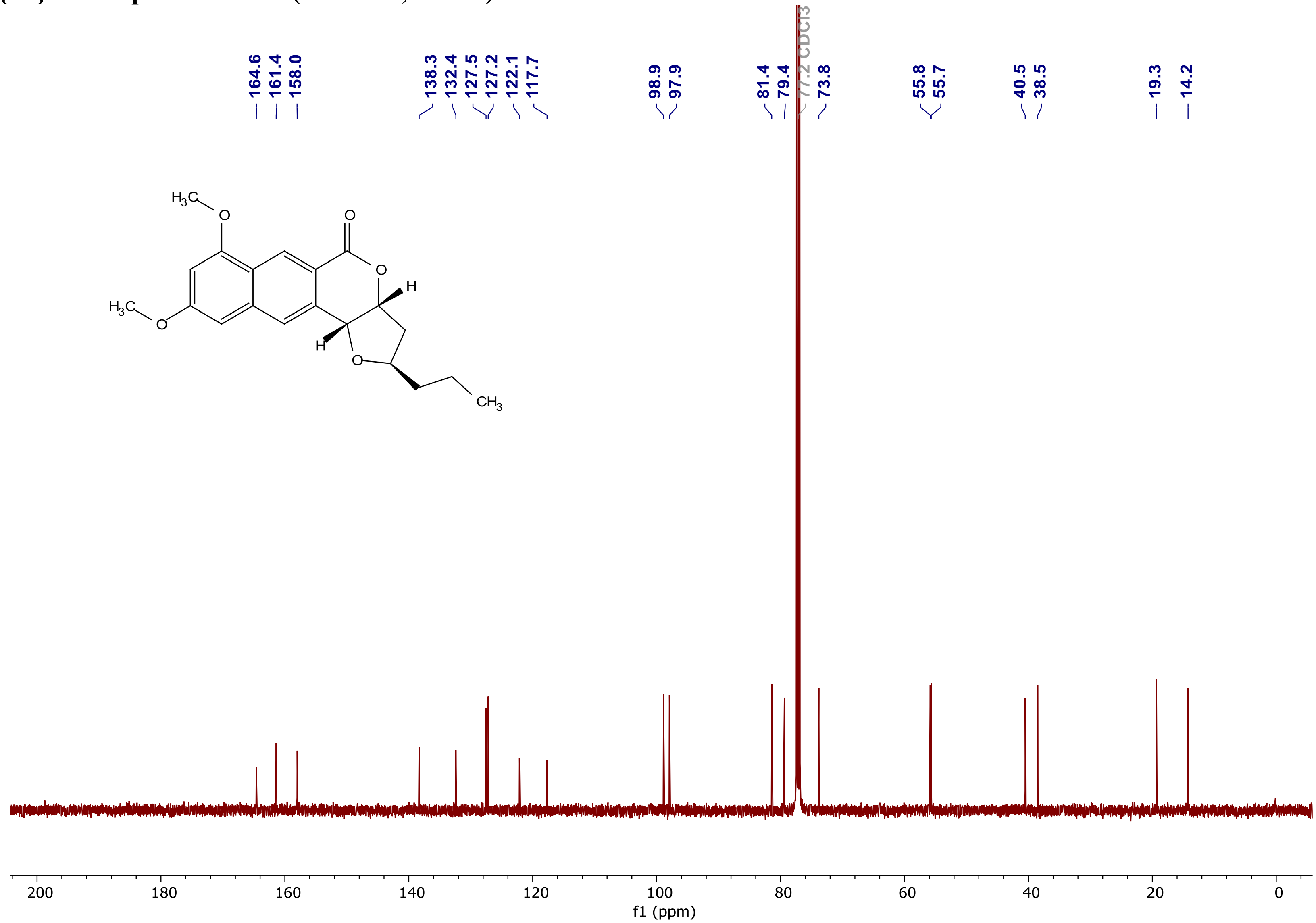

^{1}H NMR spectrum of compound 26 (500 MHz, $CDCl_3$):

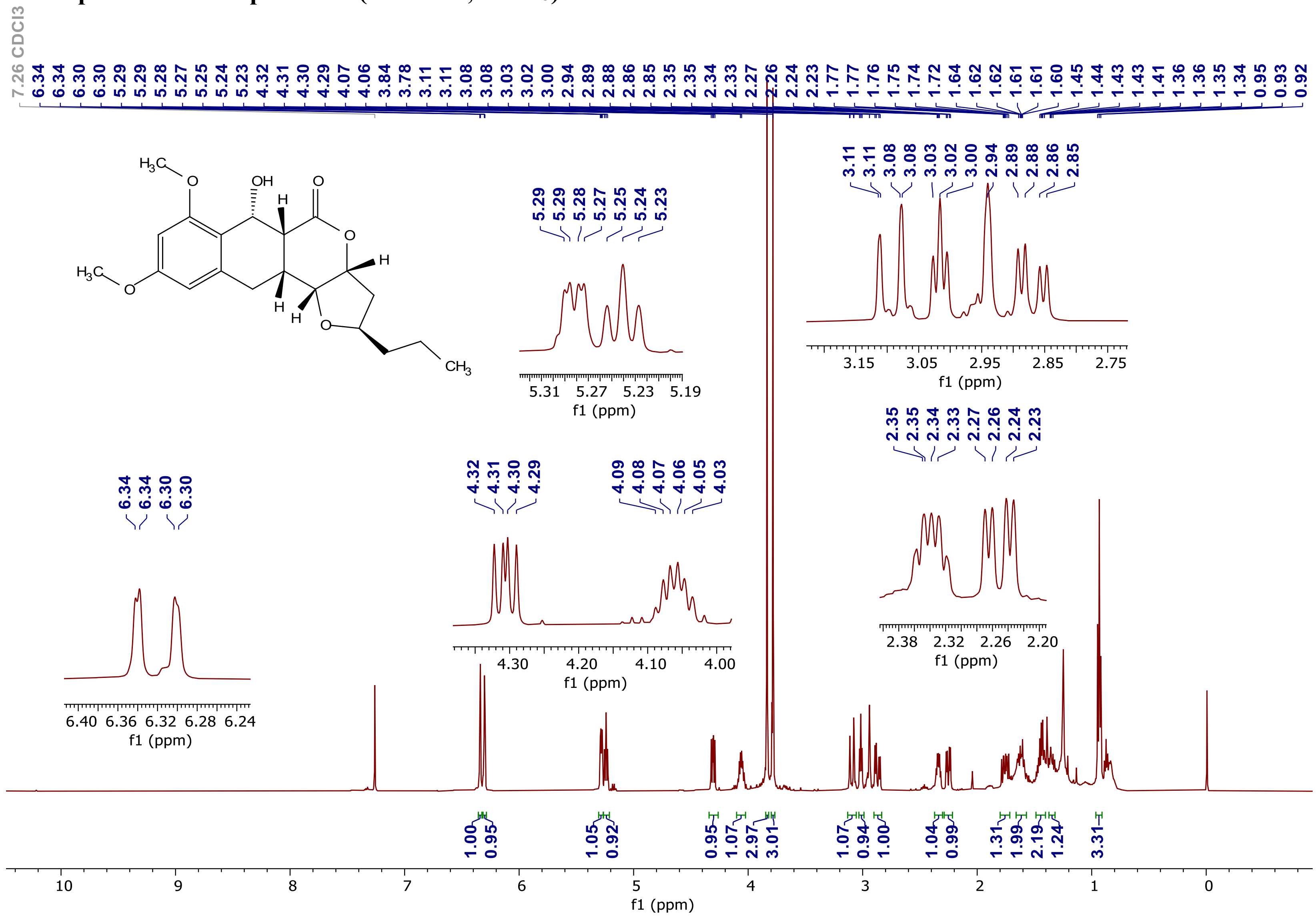

$^{13}C\{^1H\}$ NMR spectrum of compound 26 (125 MHz, $CDCl_3$):

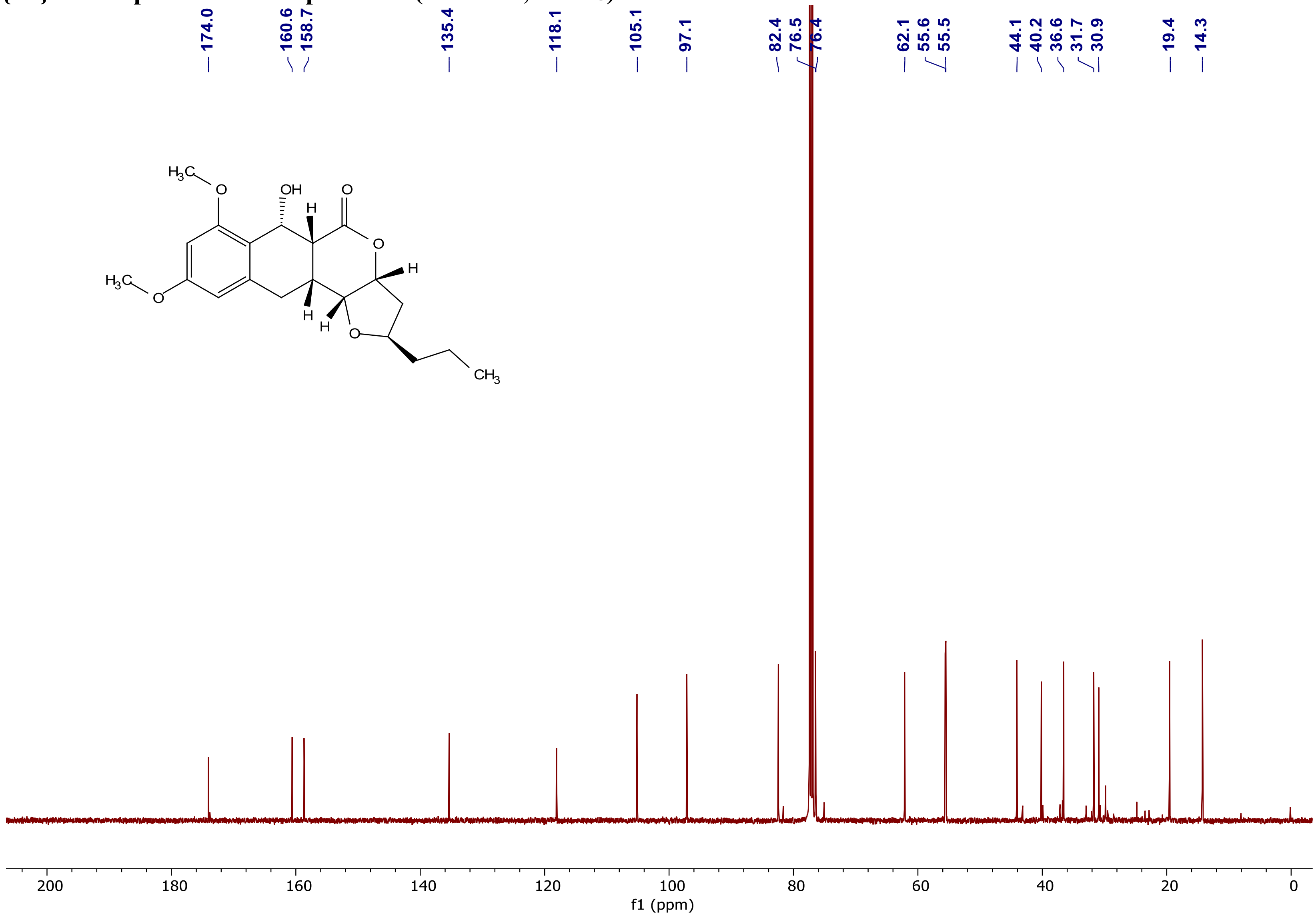

^{1}H NMR spectrum of compound 27 (500 MHz, $CDCl_3$):

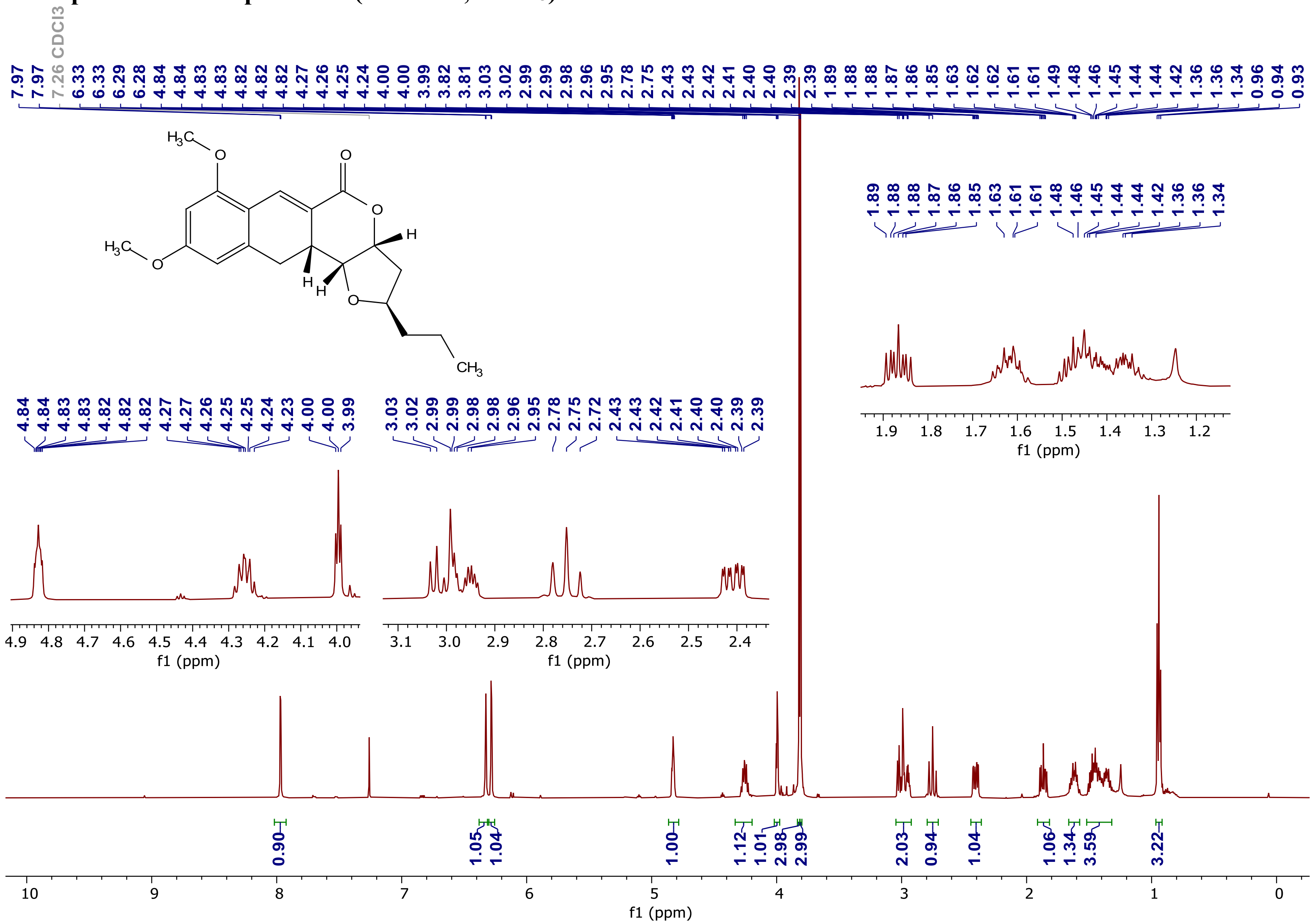

$^{13}C\{^1H\}$ **NMR spectrum of compound 27 (125 MHz, $CDCl_3$):**

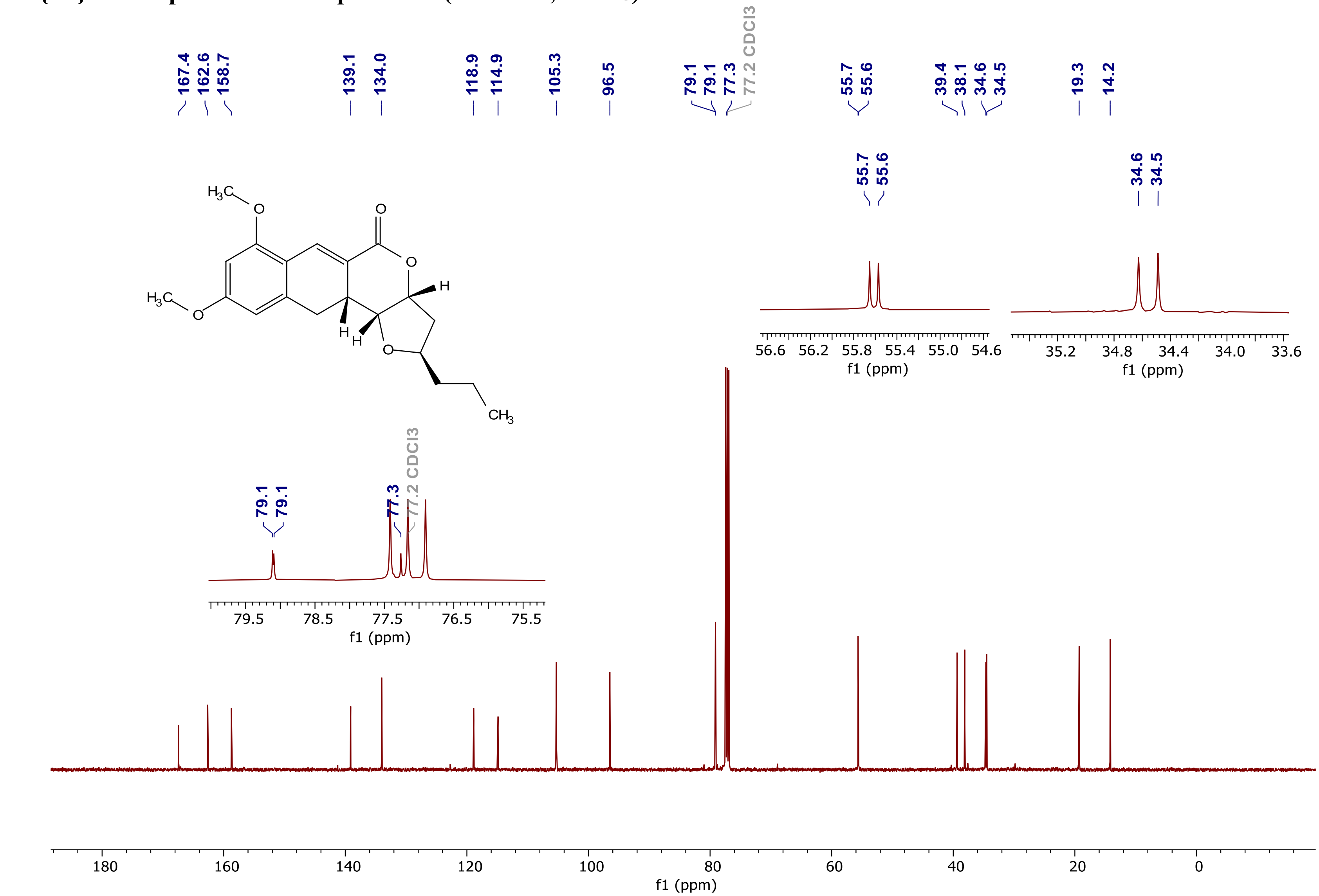

^{1}H NMR spectrum of compound 13 (500 MHz, $CDCl_3$):

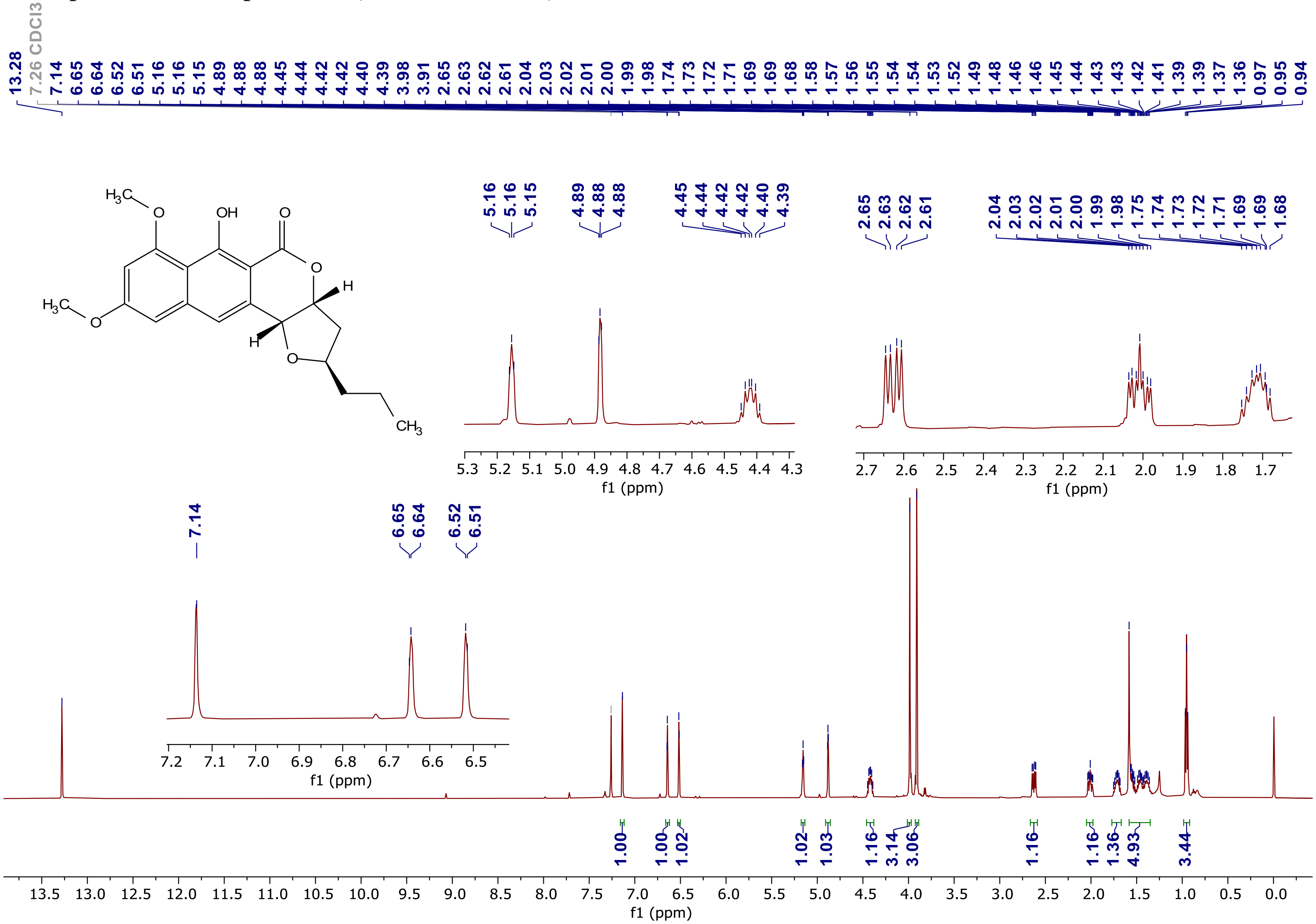

$^{13}C\{^1H\}$ NMR spectrum of compound 13 (125 MHz, $CDCl_3$):

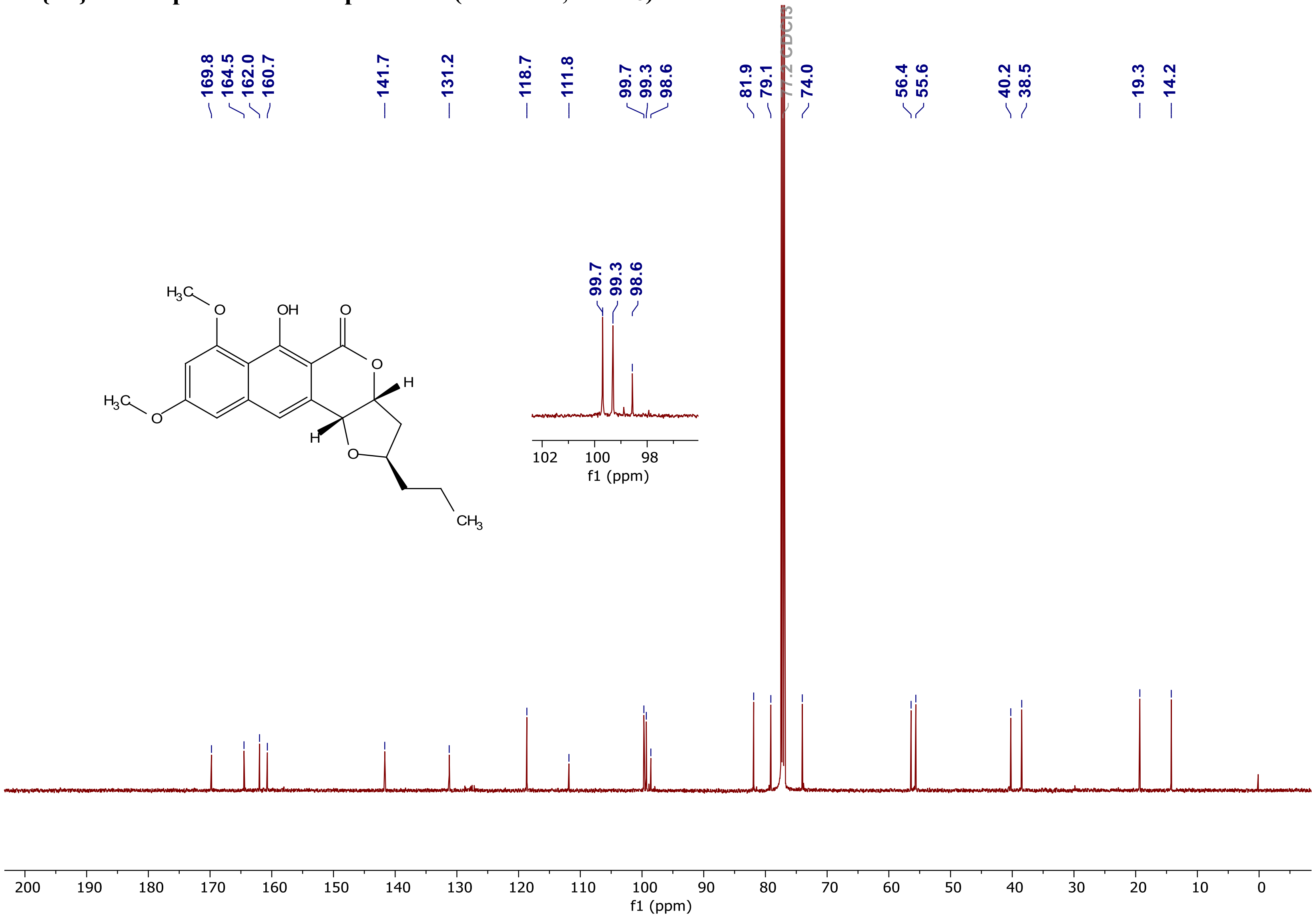

Supplement: Supplementary file 1 — jo3c02231_si_001.pdf [file jo3c02231_si_001.pdf]
